# Integrative omics analysis. A study based on *Plasmodium falciparum *mRNA and protein data

**DOI:** 10.1186/1752-0509-8-S2-S4

**Published:** 2014-03-13

**Authors:** Oana A Tomescu, Diethard Mattanovich, Gerhard G Thallinger

**Affiliations:** 1 Core Facility Bioinformatics, Austrian Centre of Industrial Biotechnology, Graz, Austria; 2 Institute of Genomics and Bioinformatics, Graz University of Technology, Graz, Austria; 3 Cell Design and Engineering, Austrian Centre of Industrial Biotechnology, Vienna, Austria; 4 Department of Biotechnology, BOKU-VIBT University of Natural Resources and Life Sciences, Vienna, Austria; 5 Omics Center Graz, Graz, Austria

**Keywords:** integrative data analysis, co-inertia analysis, generalized singular value decomposition, integrative biclustering, life cycle stages, *Plasmodium falciparum*

## Abstract

**Background:**

Technological improvements have shifted the focus from data generation to data analysis. The availability of large amounts of data from transcriptomics, protemics and metabolomics experiments raise new questions concerning suitable integrative analysis methods. We compare three integrative analysis techniques (co-inertia analysis, generalized singular value decomposition and integrative biclustering) by applying them to gene and protein abundance data from the six life cycle stages of *Plasmodium falciparum*. Co-inertia analysis is an analysis method used to visualize and explore gene and protein data. The generalized singular value decomposition has shown its potential in the analysis of two transcriptome data sets. Integrative Biclustering applies biclustering to gene and protein data.

**Results:**

Using CIA, we visualize the six life cycle stages of *Plasmodium falciparum*, as well as GO terms in a 2D plane and interpret the spatial configuration. With GSVD, we decompose the transcriptomic and proteomic data sets into matrices with biologically meaningful interpretations and explore the processes captured by the data sets. IBC identifies groups of genes, proteins, GO Terms and life cycle stages of *Plasmodium falciparum*. We show method-specific results as well as a network view of the life cycle stages based on the results common to all three methods. Additionally, by combining the results of the three methods, we create a three-fold validated network of life cycle stage specific GO terms: Sporozoites are associated with transcription and transport; merozoites with entry into host cell as well as biosynthetic and metabolic processes; rings with oxidation-reduction processes; trophozoites with glycolysis and energy production; schizonts with antigenic variation and immune response; gametocyctes with DNA packaging and mitochondrial transport. Furthermore, the network connectivity underlines the separation of the intraerythrocytic cycle from the gametocyte and sporozoite stages.

**Conclusion:**

Using integrative analysis techniques, we can integrate knowledge from different levels and obtain a wider view of the system under study. The overlap between method-specific and common results is considerable, even if the basic mathematical assumptions are very different. The three-fold validated network of life cycle stage characteristics of *Plasmodium falciparum *could identify a large amount of the known associations from literature in only one study.

## Background

Continuous technological improvements facilitate the availability of large amounts of omics data, resulting from the simultaneous characterization on different levels (genome, transcriptome, proteome and metabolome) of an organism or an experimental condition. Regulatory mechanisms captured in this way provide a complex multi-level view of the system under study. In order to exploit the measured data to the maximum, one has to integrate all available data sets into a single analysis framework. Methods that apply analysis techniques simultaneously to more than one data set are called integrative analysis methods. The data sets can characterize one organism on different levels [1], or they can be measured on the same omics level but on different organisms/platforms [2,3]. Here we focus on the first scenario.

Integrative analysis methods provide a deeper understanding of the system under study through the meaningful combination of multi-level omics data. The integrated omics data differ from study to study. There are studies that integrate, for example, gene expression and methylation data [4], somatic mutations, copy number and gene expression data [5], chromatin maps and gene expression profiles [6], genotypic variation at DNA level and gene expression data [7], CHIP-seq and RNA-seq data [8], transcriptomics and proteomics data [1,9,10]. In this study we apply integrative analysis to transcriptomics and proteomics data.

With transcriptomic and proteomic data, most analysis techniques are based on the direct correlation between transcripts and proteins. Cox and colleagues [10] present different approaches based on correlation and clustering. Other correlation-based studies have also been performed in [9,11-16]. Statistical methods based on correlations are presented in [17,18]. The premise of a direct correlation between transcripts and proteins is not valid in eucaryotic organisms, due to post-transcriptional and post-translational regulation [1,19]. Other approaches are based on network analysis [20,21] and statistical methods such as analysis of variation, clustering and gene set enrichment [22-24]. Piruzian *et al*. [25] revealed similarities in regulation at transcriptomic and proteomic levels and identified potential key transcription factors and new signaling pathways for psoriasis using a network based approach, which employed overconnection analysis, hidden node analysis and rank aggregation. Perco *et al*. [26] integrated transcriptomics and proteomics on the level of protein interaction networks. They started with the modest overlap between the data sets, which increased substantially on the level of protein interaction networks and in this way, amplified the joint functional interpretation of the omics data sets. In a study by Hahne and colleagues [22] analysis of variation, k-means clustering and functional annotation were applied to transcriptome and proteome data from salt-stressed *B. subtilis *cells. They showed a well-coordinated induction of gene expression and changes of the protein levels as the result of a severe salt shock. Verhoef *et al*. characterized the changes associated with *ρ*-hydroxybenzoate production in the engineered *P. putida *strain S12, integrating genes and proteins as well as cluster and pathway analysis [23]. In [24], Takemasa *et al*. applied gene ontology analysis (GO) to transcriptome and proteome data from human colorectal cancer samples, which led to a better understanding of functional inference at the physiological level and to potential drug targets. Other integrative approaches can be found in [27-29] for omics data in general and in [19] for transcriptome and proteome data in particular.

In this article, we focus on the comparison of three integrative analysis techniques of mRNA and protein abundance data. We selected methods meeting the following criteria: (i) they are based on a clear mathematical formulation, (ii) they are as different as possible from one another and, the most important argument, (iii) they allow the analysis of all measured data (not limited to pairs of genes and proteins). Based on these criteria we have chosen: Co-inertia analysis (CIA), which is an integrative analysis method used to visualize and explore gene and protein data [1,30], Generalized singular value decomposition (GSVD), which has shown its potential in the analysis of two transcriptome data sets [3] and Integrative biclustering (IBC), which applies biclustering to gene and protein data [31].

We compare CIA, GSVD and IBC by applying them to mRNA and protein abundance data from a study of *Plasmodium falciparum*, [9], the parasite causing malaria in humans. The data in this study was gathered from samples for the six life cycle stages: merozoite, ring, trophozoite, schizont, gametocyte and sporozoite. For the comparison, we add additional information in the form of GO [32] terms for biological processes.

Using CIA, we visualize the six life cycle stages and GO terms in a 2D plane and interpret the spatial configuration. With GSVD, we decompose the data sets into matrices with biologically meaningful interpretations and explore the processes captured by the data sets. IBC identifies groups of genes, proteins, GO terms and life cycle stages revealing functional modules of *P. falciparum*.

We compare the results of the three integrative analysis methods based on the association of GO terms to the six life cycle stages and show common as well as method-specific results. The common results are presented in form of a three-fold validated network view of the biological processes activated in each life cycle stage. To the best of our knowledge such a complete, GO terms based, characterization of *P. falciparum *was not published before.

## Methods

### Data set

We analyse a publicly available data set containing mRNA and protein abundance data from the six life cycle stages of *P. falciparum *[9,33]. Microarray [33] and proteomic analyses [9] were carried out on *P. falciparum *clone 3D7. Gene expression levels were measured with a custom oligonucleotide array and computed with the match-only integral algorithm (MOID). Proteins were detected by multidimensional protein identification technology (MudPIT), and protein abundance was estimated by the number of MS/MS spectra identified per protein. In total, 4294 genes and 2903 proteins were measured in all six life cycle stages. For the analysis, we created a matrix for each data set where the genes and proteins are represented as rows and the life cycle stages as columns.

Additionally, GO [32] information on biological processes in *P. falciparum *were employed. We used the R [34] packages *org.Pf.plasmo.db *[35] and *GO.db *[36], which provide *P. falciparum *specific mappings of genes to GO terms as well as additional information on GO terms. Based on these two annotation databases, 3283 of 4294 genes and 2491 of 2903 proteins were associated with 614 GO terms. For each data set, a GO matrix with the same number of rows as the corresponding expression data set was created. The columns of the GO matrix hold data describing the gene/protein affiliation to a certain GO term. If a gene/protein is associated with that GO term, the strength of the affiliation is computed as the ratio between 1 and the total number of genes/proteins associated with the GO term. CIA and IBC use directly the computed GO matrix. GSVD performs a GSE analysis based on *org.Pf.plasmo.db *and *GO.db*. In this way, we make sure that all three methods are applied to the same data sets. Additional file 1 contains the GO terms with their GO names used in this study.

Our study comprises the analysis of four data sets: mRNA abundance data, protein abundance data, a GO matrix of mRNAs and a GO matrix of proteins. mRNA and protein abundances were computed with different algorithms, requiring a columnar z-score normalization of each data set. The GO matrices were computed based on the number of genes/proteins belonging to a particular GO term, which resulted in equal ranges of the entries in the matrices. Afterwards, they were joined and z-transformed. Columns (GO terms) that included only entries equal to zero (none of the associated genes or proteins was measured in the data set) were deleted before the normalization. After deletion, 614 GO terms were available for further analysis.

### CIA

CIA was introduced by Dolèdec and Chessel [30] as an extension of Tucker's inter-battery method [37] for the study of species-environment relationships of ecology data. CIA was applied to genome and *agr *groups data of *S. aureus *[38] and to physico-chemical properties of amino-acids and the amino-acid composition of *E.coli *proteins [39]. Culhane *et al*. [40] applied this method to two gene expression data sets, and Fagan *et al*. [1] used it as an integrative analysis method for gene and protein data.

**Figure 1:**
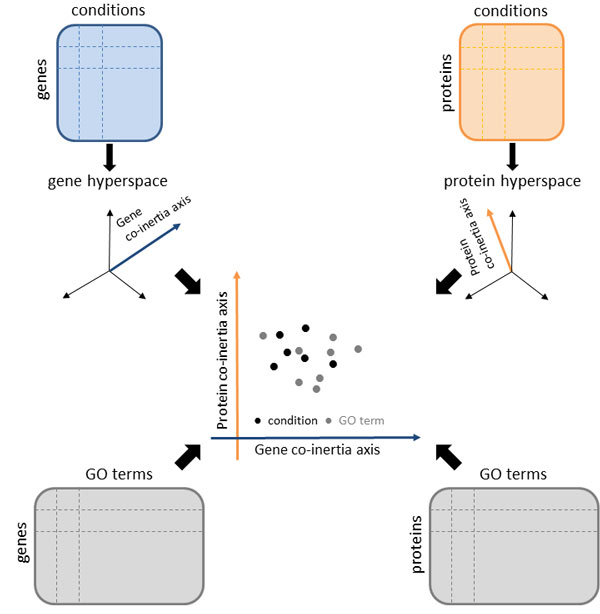
**Flowchart of CIA**. The gene expression matrix (blue) contains the genes in the rows and the conditions in the columns. The protein expression matrix (orange) contains the proteins in the rows and the conditions in the columns. The conditions in our study correspond to the life cycle stages of *P. falciparum*. Genes and proteins annotated to the considered GO terms are gathered in a separate matrix (gray). The gene and the protein expression matrices are transformed into a new hyperspace. The pair maximizing the covariance is computed from the axes spanning the two hyperspaces. These two axes (gene and proteins co-inertia axes) span a new space where the conditions (life cycle stages) can be plotted. Additionally, GO terms can be projected into the CIA space and visualized together with the life cycle stages. Figure adapted from [30].

CIA is a multivariate analysis method that identifies relationships between two data sets by maximizing the covariance between them. CIA starts by performing a multivariate analysis like principal component analysis (PCA) [41], non-symmetrical correspondence analysis (NSC) [42] or correspondence analysis (CA) [43] on each individual data set. The produced results are a set of principal axes that maximize the projected variability (inertia) of each data set independently. Each set of axes spans a new multidimensional gene and protein space. Based on the computed axes, CIA identifies one axis in each new multidimensional space on which the projected data sets present maximal covariance and simultaneously maximal standard deviations. Thereby, CIA maximizes the covariance between the two data sets. Global correlation or co-structure between the data sets is measured by the RV coefficient [44]. For mathematical details on CIA, please refer to [30]. CIA computation steps are summarized in a concise flowchart in Figure 1. CIA is available in the R packages *made4 *[45] and *ade4 *[46].

CIA has two major advantages: It can be applied to data sets with considerable more variables (genes and proteins) than samples (life cycle stages), and the variables in the two data sets do not have to match one another.

Additional information such as GO annotations can be superimposed on the CIA plots. This overlay was already done for CA [47], and is also possible for CIA [1]. GO term projections are obtained by first normalizing the two GO matrices in the same way as the expression data sets and then multiplying them by the weights of the genes/proteins resulting from the NSC, followed by CIA analyses. The projection scores computed in this way show GO term associated with the measured genes/proteins in relation to the life cycle stages.

### GSVD

The GSVD was developed as an extension of the singular value decomposition (SVD) that was already used directly as an analysis method [48,49] and indirectly as part of a PCA [50,51]. In a study by Golub *et al*. [52], GSVD was used as a comparative analysis method [3] for two gene expression data sets of cell cycle data from yeast and humans.

GSVD is based on the joint decomposition of both data sets as shown in equations (1) and (2):

(1) $$G=U_{1}\Sigma_{1}X^{-1}$$

(2) $$P=U_{2}\Sigma_{2}X^{-1}.$$

Matrices *G *and *P *contain the gene and protein abundance data. The rows of the common matrix *X*^-1 ^are named genelets. In [3] it was shown that these genelets can be regarded as processes captured by both data sets. The genelets are expressed only in the corresponding arraylets (columns of *U*_1 _and *U*_2_) with a relative significance measured by the generalized eigenvalues (*σ*_1,*i *_, *σ*_2,*i*_) from the diagonals of Σ_1 _and Σ_2_. The relative significance of a genelet in the gene data set relative to the protein data set is measured by an antisymmetric angular distance calculated as shown in equation (3):

(3) $$\theta_{i}=\arctan\left(\frac{\sigma_{1,i}}{\sigma_{2,i}}\right)-\frac{\pi }{4}.$$

An angular distance between *-π/*4 and *-π/*8 represents a high significance of the *i^th ^*genelet in the second data set relative to the first data set. If the value of the angular distance ranges between *π/*8 and *π/*4, then the *i^th ^*genelet has a high significance in the first data set relative to the second data set. The *i^th ^*genelet shows equal significance in both data sets if the angular distance ranges between *-π/*8 and *π/*8 (see equation (4)). In our study, the first matrix contains mRNA abundance data, the second matrix protein abundance data and significances are assigned as follows:

(4) $$\theta_{i}\in \left\{\begin{array}{cc} \left[-\pi /4,-\pi /8\right] & \text{protein}\;\text{space}\\ \left[-\pi /8,\pi /8\right] & \text{gene}\;\text{and}\;\text{protein}\;\text{space}\\ \left[\pi /8,\pi /4\right] & \text{gene}\;\text{space}\text{.} \end{array}\right)$$

**Figure 2:**
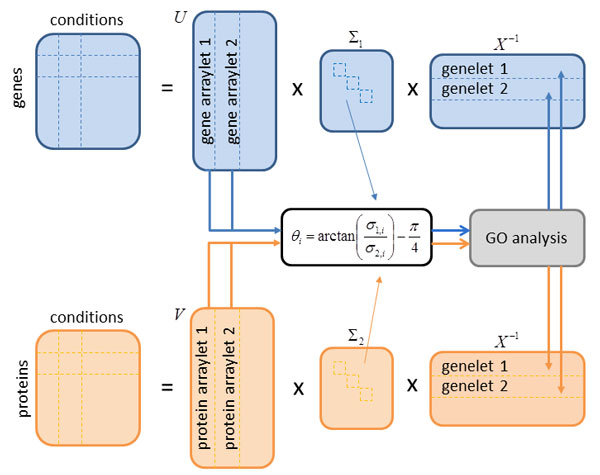
**Flowchart of GSVD**. The gene expression matrix (blue) contains the genes in the rows and the conditions in the columns. The protein expression matrix (orange) contains the proteins in the rows and the conditions in the columns. The conditions in our study correspond to the life cycle stages of *P. falciparum*. Genes and proteins annotated to the considered GO terms are gathered in a separate matrix (gray). The gene and the protein expression matrices are each decomposed in three matrices according to equations (1) and (2). The matrices *U *and *V *contain the arraylets, which encode for the expression of genes and proteins in the corresponding genelets *X*^-1^, which represent the cellular state in the measurement conditions. According to the angular distance *θ_i_*, which is computed from the generalized eigenvalues *σ*_1,*i *_and *σ*_2,*i *_, a restricted GSE analysis is performed on the genes and/or proteins with the absolute (from a mathematical point of view) highest values in the arraylets in order to assign GO terms to the corresponding genelets. Figure adapted from [3].

A summary of the computation flow is shown in a block diagram in Figure 2.

Alter *et al*. [3] used a Mathematica implementation of a numerically robust GSVD algorithm based on [52,53], which we reimplemented in R.

In order to discover the processes captured by the genelets, a restrictive gene set enrichment (GSE) analysis is performed on 50% of the genes and/or proteins showing the highest absolute values in the corresponding arraylets. The GSE analysis is performed with the R package *GOstats *[54], which computes the statistically significantly enriched GO terms based on the hypergeometrical distribution.

### IBC

**Figure 3:**
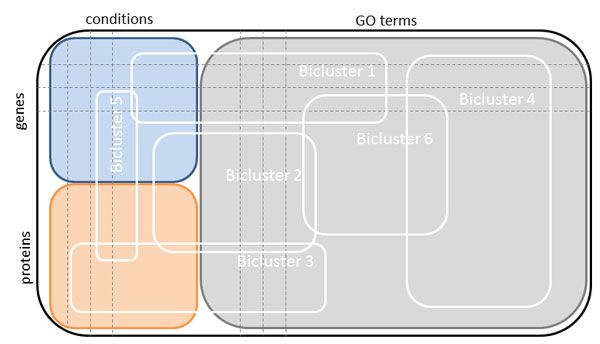
**Block diagram of IBC**. The gene expression matrix (blue) contains the genes in the rows and the conditions in the columns. The protein expression matrix (orange) contains the proteins in the rows and the conditions in the columns. The conditions in our study correspond to the life cycle stages of *P. falciparum*. Genes and proteins annotated to the considered GO terms are gathered in a separate matrix (gray). The three matrices are combined to a new matrix, which is subjected to biclustering. The resulting biclusters can include genes, proteins, conditions, GO terms or any combination of these.

The basic idea of biclustering (co-clustering or two-way clustering) was presented in [55], but it took almost thirty years until the method was applied to gene expression data [56]. In the last two decades, biclustering has become more and more popular [57-59]. In contrast to clustering, where either rows *or *columns are clustered, biclustering performs clustering of rows *and *columns simultaneously. The members of the obtained biclusters are as similar to one another and as different from the other biclusters as possible. Figure 3 presents how mRNA abundances, protein expression and GO terms are assembled to a complete data set and how the resulting biclusters could look like.

There are four types of possible biclusters as reviewed in [31,60,61]. Biclusters can have (i) equal values over rows and columns as well as (ii) equal values over rows or columns. They can also have (iii) coherent values, which means that each column or row can be computed by adding or multiplying a constant to the previous column or row. The forth type of bicluster has (iv) coherent evolutions, which means that the exact value of a matrix entry is not important, but whether the values increase or decrease over rows or columns. The biclustering algorithm used here is included in the R package *biclust *[62].

The types of computed biclusters vary. There are single biclusters where only one bicluster is found in the whole data set as well as exclusive rows and/or exclusive columns biclusters. Non-overlapping and non-exclusive biclusters can also be computed. The fifth type is the arbitrarily positioned overlapping biclusters. Graphical representations of the different categories of biclusters can be found in [31].

Most of the biclustering algorithms implemented depend on the starting point of the search and thus may lead to different results in consecutive runs. Additionally, biclustering does not result in a perfect data separation, as overlapping biclusters are possible. As a remedy, the *biclust *package provides a robust method that delivers stable and reliable results. This function includes the repeated use of one algorithm in combination with several parameter settings and/or subsamples of the data. A modified version of the Jaccard index is used for the combination of the resulting biclusters, which in case of two biclusters takes into account the fraction of row-columns combinations in both biclusters to all row-column combinations. For detailed mathematical definitions, please refer to [31].

Analogous to integrative clustering, we define integrative biclustering as the biclustering of two or more data sets. Integrative clustering was already applied to copy number and gene expression data in order to identify novel breast tumours subgroups [63]. Mo and colleagues [5] describe integrative clustering of genomic, epigenomic and transcriptomic profiling.

Integrative biclustering was applied to gene expression, protein interaction, growth phenotype and transcription factor binding data in [64] in order to reveal modularity and organization in the yeast molecular network. We apply integrative biclustering to a matrix consisting of the mRNA and protein abundance data and of the corresponding GO matrices. The genes and the proteins are represented by rows, whereas the samples and the GO terms by columns. Before biclustering can be carried out, discretization is necessary. Here the built-in function *discretize *of the R package *biclust *[31] was used. After appropriate processing, the result of IBC was loaded into Cytoscape [65] to obtain a network view of the associations.

## Results and discussion

Results of each analysis method can be divided into method-specific associations and general associations. The general associations are used to compute results common to all three methods.

### CIA

**Figure 4:**
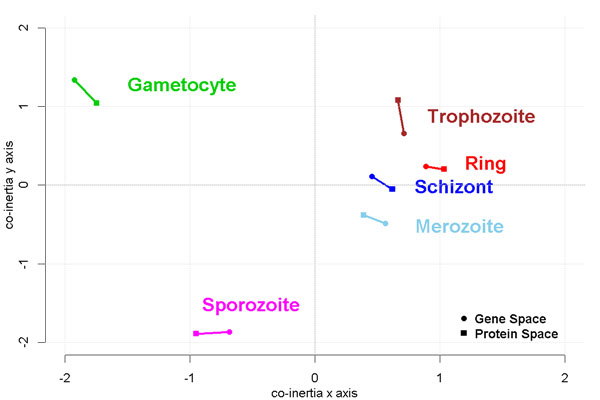
**Co-inertia analysis - results**. CIA offers the possibility to visualize the gene and protein space projections of the six life cycle stages of *P. falciparum *in one plot. The projection in gene space are represented by circles and in the protein space by squares. For each life cycle stage, the two corresponding projections are connected through a line. We observe that the y axis separates the intraerythrocytic cycle from the stages gametocyte and sporozoite.

**Figure 5:**
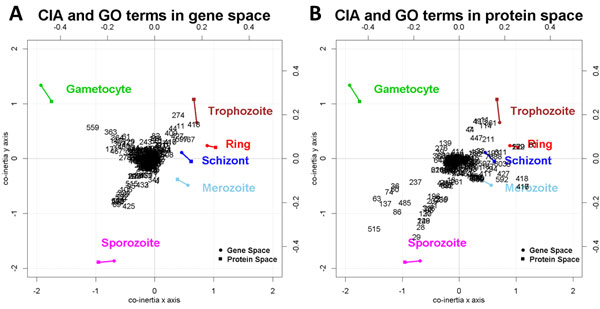
**Co-inertia analysis and GO terms - results**. In addition to the life cycle stages, GO terms can also be projected into the CIA plot. A) projections of the GO terms in gene space and B) projections of the GO terms in the protein space. Each GO term is represented by a number. Please note that the life cycle stages and the GO terms are plotted on different scales. The lower and left axes represent the life cycle stages and the upper and right axes represent for the GO terms. In gene space we observe a clear projection of the GO terms in the direction of gametocytes and sporozoites. In protein space, GO terms are projected clearly in direction of sporozoites and the intraerythrocytic cycle.

With CIA we visualize the six life cycle stages in the gene and protein space (Figure 4). We observe that the co-inertia × axis separates the intraerythrocytic cycle stages (trophozoite, ring, schizont, merozoite) from gametocytes and sporozoites. In the erythrocytes, the cycle begins with the ring stage, followed by the trophozoite stage. Trophozoites mature into schizonts, which cause the rupture of blood cells resulting in the release of merozoites. In Figure 1, this exact sequence within the intraerythrocytic cycle can be observed. The sporozoites are the sexual stage of the mosquito and will be released in the blood stream of the infected organism. The ring stage can develop into a gametocyte and can be ingested by a mosquito. In addition to the life cycle stages, GO terms can also be represented through projections in the CIA plot (Figure 5).

A mapping between numbers and GO terms can be found in Additional file 1. Detailed representations of the division limits for the specific and general associations are shown in Additional file 4.

#### General associations

**Figure 6:**
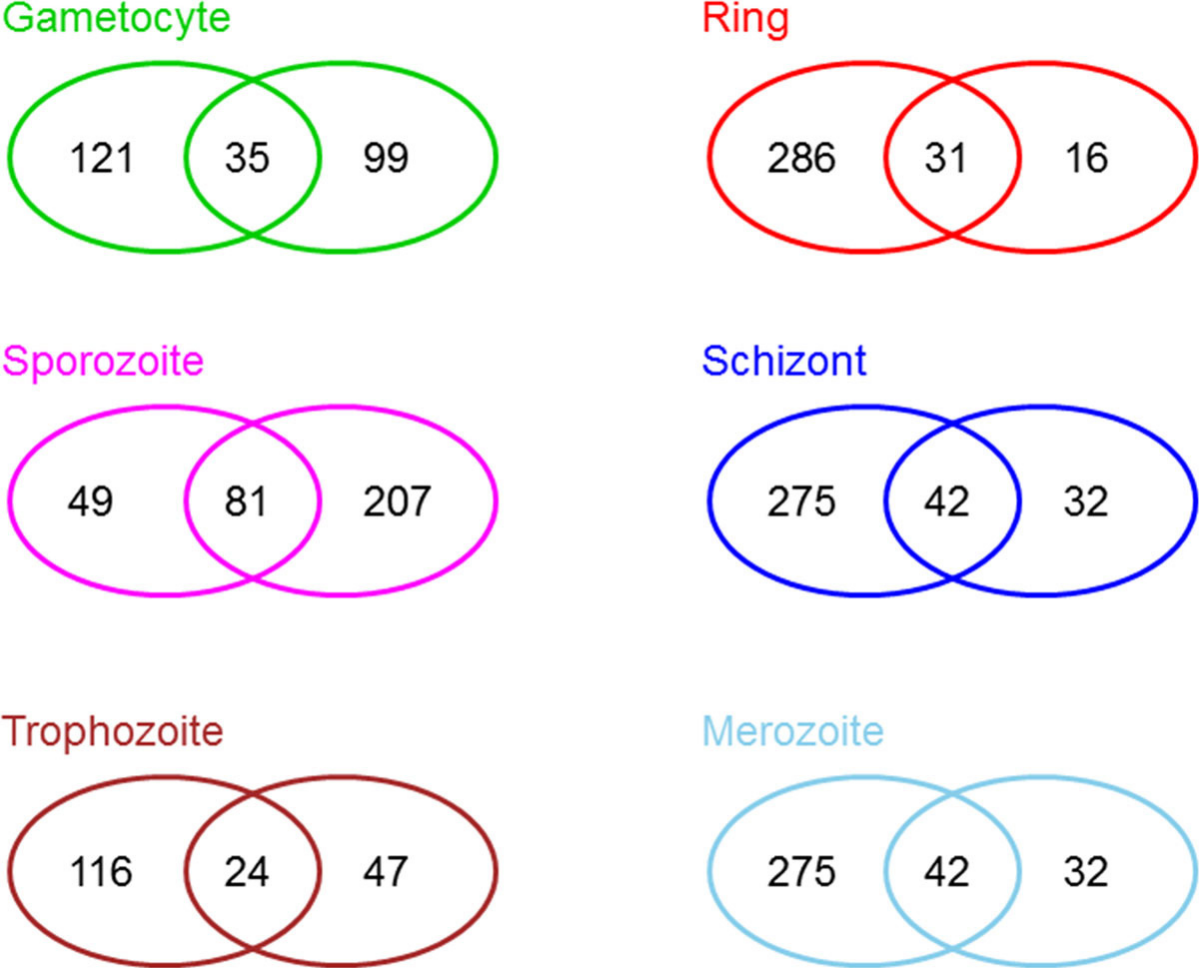
**CIA general associations - overlap between gene and protein space**. For each life cycle stage, the left ellipse shows the number of general GO term associations in gene space whereas the right ellipse shows the number of general GO term associations in protein space. The amount of identical GO terms is shown in the overlapping region of the ellipses. In general, more GO term associations emerge form gene space than from protein space. Two exceptions can be observed: the sporozoite stage, where more associations are found in protein space and the gametocyte stage, where a similar number of associations is found in each space. These tendencies can be also observed in Figure 7.

General associations resulting from CIA are distributed as follows: In gene space, GO terms in the first trigonometric quadrant are associated with trophozoites, GO terms in the second quadrant with gametocytes and GO terms in the third quadrant with sporozoites. GO terms in the first and forth quadrant, which were not identified as specific for trophozoits are associated with rings, schizonts and merozoits. Due to the proximity of stages in the CIA gene space, a more specific distribution to each stage is not possible. In protein space the associations are produced as follows: For gametocytes and sporozoites, we follow the same criteria as in gene space. For the distribution of GO terms to trophozoits, rings, schizonts and merozoits, we divide the first and forth quadrant in three sectors. GO terms that form angles of at least 30 degrees with the positive co-inertia × axis are associated with trophzoits. GO terms with an angle between -10 and 30 degrees are associated with rings and schizonts. GO terms with an angle wider than -10 degrees are associated with merozoits. Since GSVD and IBC discover associations only in common space (gene and protein space), the CIA associations for each life cycle are computed as the set union of the associations in gene and the associations in protein space. These general associations are shown in Additional files 2 and 3. The overlap between the general association in gene and protein space are shown in Figure 6.

#### Method-specific associations

In addition to the general results, method-specific associations of GO terms with life cycle stages are observed. For these associations, the direction of the projected GO terms is considered. From the general associations, we take those GO terms that have a distance of at least 0.1 to the origin of the coordinate systems. An exception is made for gametocytes in protein space. A threshold of 0.05 is more appropriate here due to the spacial distribution of GO terms relative to the origin. These considerations result in GO term associations with gametocyte, trophozoite and sporozoite in gene space. Details are presented in Table 1 and Additional file 5. In the protein space, clear GO term associations with gametocyte, sporozoite, trophozoite and merozoite stages are found (Table 2 and Additional file 6). Additional file 6 also includes associations with the stages ring and schizont.

**Table 1:** CIA specific GO term association in gene space to the gametocyte stage.

CIA: Gametocyte in gene space	
61	GO:0006334: nucleosome assembly

145	GO:0006072: glycerol-3-phosphate metabolic process

171	GO:0006465: signal peptide processing

362	GO:0007131: reciprocal meiotic recombination

363	GO:0002720: positive regulation of cytokine production involved in immune response

364	GO:0006359: regulation of transcription from RNA polymerase III promoter

467	GO:0051604: protein maturation

480	GO:0001819: positive regulation of cytokine production

559	GO:0006071: glycerol metabolic process

In this table GO term association in gene space to the life cycle stage gametocyte are presented. The numbers in the left column correspond to the numbers in graphic A of Figure 5

**Table 2:** CIA specific GO term association in protein space to the gametocyte stage.

CIA: Gametocyte in protein space	
1	GO:0009405: pathogenesis

39	GO:0007165: signal transduction

64	GO:0007155: cell adhesion

139	GO:0045454: cell redox homeostasis

276	GO:0044262: cellular carbohydrate metabolic process

366	GO:0006103: 2-oxoglutarate metabolic process

In this table GO term association in protein space to the life cycle stage gametocyte are presented. The numbers in the left column correspond to the numbers in graphic B of Figure 5

According to Figure 5, where for readability reasons GO terms are represented by numbers from 1 to 614, some of the most remarkable associations in gene space are: GO:0006071 *glycerol metabolic process *(559) and GO:0002720 *positive regulation of cytokine production *(363) for gametocytes; GO:0006101 *citrate metabolic process *(425) and GO:0016255 *attachment of GPI anchor to protein *(89) for sporozoites; GO:0006591 *ornithine metabolic process *(274) and GO:0006094 *gluconeogenesis *(418) for trophozoites. In protein space we observe: GO:0045454 *cell redox homeostasis *(139) and GO:0044262 *cellular carbohydrate metabolic process *(276) for gametocytes; GO:0006928 *cellular component movement *(551) and GO:0015991 *ATP hydrolysis coupled proton transport *(29) for sporozoites; GO:0006412 *translation *(11) and GO:0019538: *protein metabolic process *(361) for trophozoites; GO:0006334 *nucleosome assembly *(61) and GO:0050776 *regulation of immune response *(8) for ring and schizonts; GO:0042594 *response to starvation *(592), GO:0000045 *autophagic vacuole assembly *(416) and GO:0002253 *activation of immune response *(417) for merozoites.

The overlap between the projections in gene and protein space is modest. Three GO terms were projected in the direction of trophozoites in gene and in protein space: GO:0006412 *translation *(11), GO:0006414 *translational elongation *(44) and GO:0044257 *cellular protein metabolic process *(114).

### GSVD

**Figure 7:**
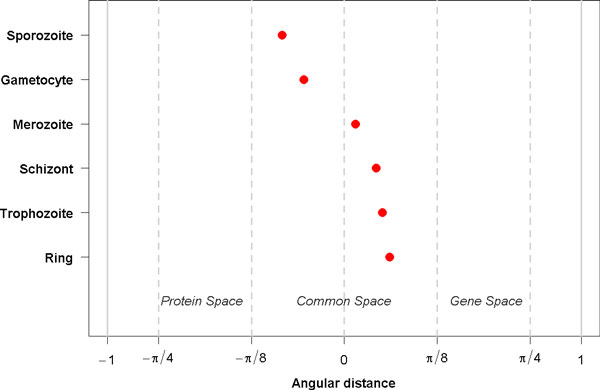
**Generalized singular value decomposition - angular distances**. GSVD computes angular distances between gene and protein space. In general, the angular distances map to the common space, for which restricted GSE analysis is performed on the gene and on the proteins arraylets. Nevertheless, while angular distances belonging to the intraerythrocytic cycle stages have positive values and show a tendency to the gene space, the angular distances of gametocytes and sporozoites have negative values and thus a tendency towards protein space. These preferences are also reflected by the amount of GO term associations emerging from the gene and from the protein space (see also Figure 6).

As the final step of the GSVD, a restrictive gene set enrichment analysis (GSE) is performed. The type of performed GSE analysis is based on the angular distance that encodes for each life cycle stage the significance of the gene set relative to the protein set. If the angular distances are between $-\frac{\pi }{8}$ and $\frac{\pi }{8}$, then the gene and protein data sets are of equal significance, and the GSE is conducted in the common space. The common space is defined by the gene and the protein data set. This is the case for all life cycle stages (Figure 7). If we compare the angular distance with zero, we obtain a separation of the intraerythrocytic cycle (angular distances bigger than zero) from other stages (angular distances smaller than zero). The restrictive GSE performs a GSE for each life cycle stage on 50% of the genes and proteins that present the highest absolute values in the corresponding arraylets.

#### General associations

All resulting GO terms having a p value smaller than 0.05 are considered to be general associations. These GO terms are shown in Additional file 7.

#### Method-specific associations

The method-specific GO terms are a subset of the general associations consisting of the top 15 GO terms, with the smallest p values. The method-specific associations are presented in Tables 3 and 4 and in Additional file 8. Biologically relevant associations include: GO:0051805/GO:0051807 *evasion or tolerance if immune/defense response of other organism involved in symbiotic interaction*, GO:0051832 *avoidance or defenses of other organism involved in symbiotic interaction*, and GO:0052173 *response to defenses (immune response) of other organism involved in symbiotic interaction *for trophozoites and schizonts. The other stages are associated with more general GO terms such as GO:0044237 *cellular metabolic process*, GO:0019538 *protein metabolic process *and GO:0046474 *glycerophospholipid biosynthetic process*.

**Table 3:** GSVD specific GO term association to gametocyte stage in common space.

GSVD: Gametocyte in common space	
GO:0044238	primary metabolic process

GO:0008152	metabolic process

GO:0044237	cellular metabolic process

GO:0045017	glycerolipid biosynthetic process

GO:0043170	macromolecule metabolic process

GO:0034645	cellular macromolecule biosynthetic process

GO:0046474	glycerophospholipid biosynthetic process

GO:0009059	macromolecule biosynthetic process

GO:0022613	ribonucleoprotein complex biogenesis

GO:0044260	cellular macromolecule metabolic process

GO:0019538	protein metabolic process

GO:0046486	glycerolipid metabolic process

GO:0042254	ribosome biogenesis

GO:0006839	mitochondrial transport

GO:0009987	cellular process

In this table GSVD based GO term association in common space to the life cycle stage gametocyte are presented.

**Table 4:** GSVD specific GO term association to trophozoite stage in common space.

GSVD: Trophozoite in common space	
GO:0044403	symbiosis, encompassing mutualism through parasitism

GO:0044419	interspecies interaction between organisms

GO:0051704	multi-organism process

GO:0009607	response to biotic stimulus

GO:0006952	defense response

GO:0051707	response to other organism

GO:0051805	evasion or tolerance of immune response of other organism involved in symbiotic interaction

GO:0051807	evasion or tolerance of defense response of other organism involved in symbiotic interaction

GO:0051832	avoidance of defenses of other organism involved in symbiotic interaction

GO:0051834	evasion or tolerance of defenses of other organism involved in symbiotic interaction

GO:0052173	response to defenses of other organism involved in symbiotic interaction

GO:0052564	response to immune response of other organism involved in symbiotic interaction

GO:0020033	antigenic variation

GO:0051809	passive evasion of immune response of other organism involved in symbiotic interaction

GO:0006091	generation of precursor metabolites and energy

In this table GSVD based GO term association in common space to the life cycle stage trophozoite are presented.

### IBC

**Figure 8:**
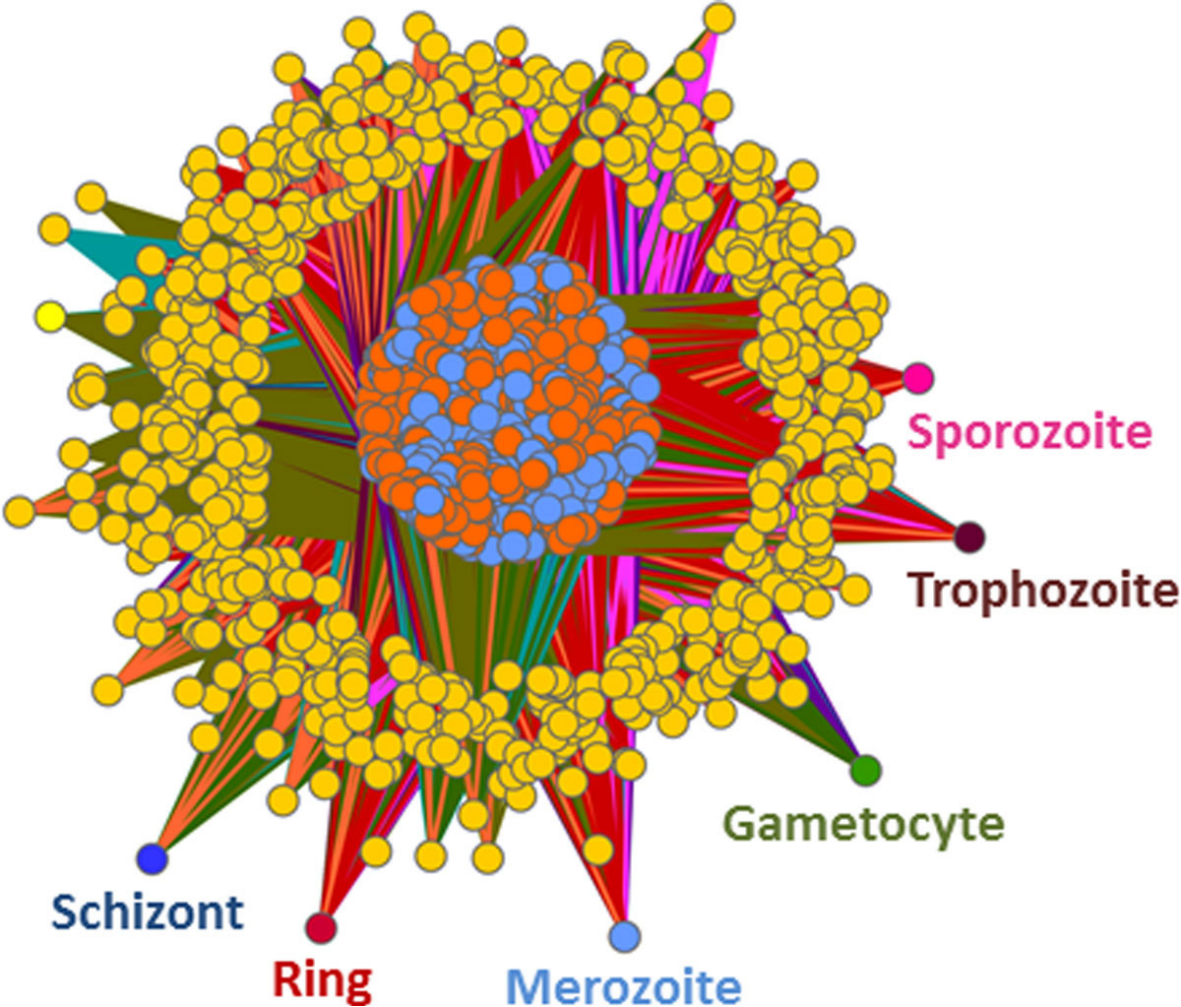
**Integrative biclustering - network view of the results**. The results of IBC were inspected and only biclusters including life cycle stages were considered for further analysis. IBC discovered 20 clusters where 9 of them contained life cycle stages and GO terms. These 9 biclusters were processed and fed into Cytoscape. An association between a life cycle stage and a GO term is represented by an edge. Different biclusters are represented by different edge colours. The life cycle stages are shown in the same colours as those used for CIA. The genes are coloured in orange, the proteins in light blue and the GO terms in yellow.

The IBC results include two types of biclusters: (i) biclusters containing genes, proteins, GO terms and life cycle conditions and (ii) biclusters containing genes, proteins and GO terms. Since we are interested in GO terms associations with life cycle stages, we will use only the first type of biclusters for further analysis. If a GO term is in the same bicluster as a life cycle stage, this GO term is associated with that life cycle stage. If there are more life cycle stages in a bicluster, the GO terms are associated with all these life cycle stages. If a life cycle stage is included in more that one bicluster, GO terms from all biclusters are associated with that life cycle stage. IBC discovered 20 biclusters and 9 of them contained life cycle stages and GO terms. A network view of the results is shown in Figure 8.

#### General and method-specific associations

Since a life cycle stage is either included in a bicluster or not and as a consequence is either associated to a GO term or not, it is not possible to distinguish between general and method-specific associations. Figure 8 shows a vast amount of genes (in orange), proteins (in light blue), GO terms (in yellow) and the six life cycle stages: gametocyte (in green), sporozoite (in pink), trophozoite (in brown), ring (in red), schizont (in dark blue) and merozoite (in light blue). The different biclusters resulting from the analysis can be identified through the colour of their edges. The exact associations with the life cycle stages are shown in Additional file 9.

### Common results

**Figure 9:**
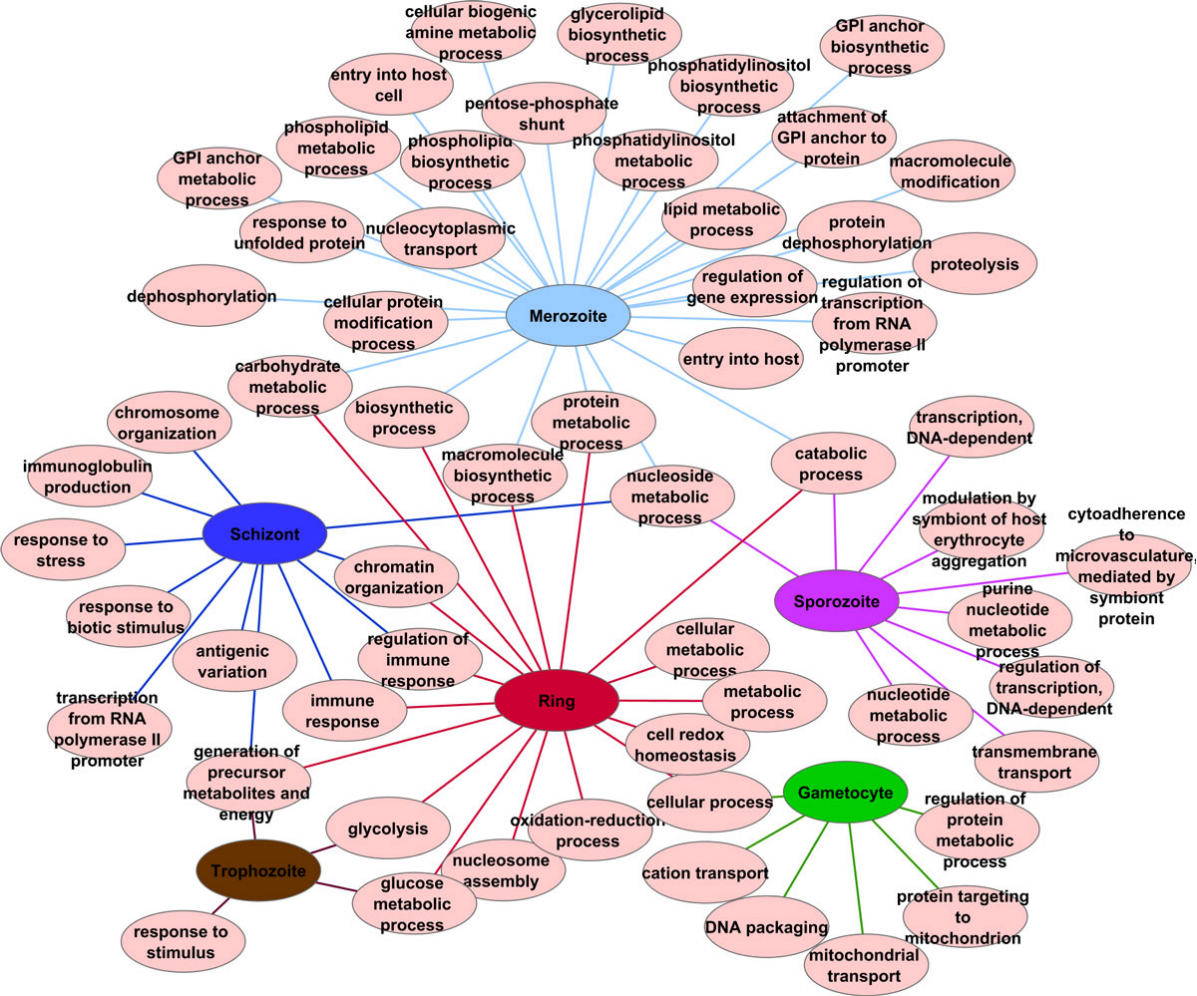
**GO terms to life cycle associations discovered by all three methods**. Network view of the GO term to life cycle stage associations discovered by all three integrative analysis methods: CIA, GSVD and IBC. We observe that gametocytes and sporozoites are loosely connected to the rest of the network underlining the separation of these stages from the intraerythrocytic cycle. Merozoites possess the largest amount of GO term associations, while trophozoites show the lowest amount of associations. Further details concerning individual stage-to-GO-term mappings are addressed in the discussion.

In this section, we present GO associations observed in all three methods. The common associations are shown in Figure 9. These associations are based on gene as well as protein information and are therefore considered to be in the common space. The GO associations computed with R were converted into a compatible format and loaded into Cytoscape. We observe here that the gametocytes are linked to the rest of the network through only one general GO term, GO:0009987 *cellular process*. The sporozoite stage is also loosely connected to the network through two GO terms, GO:0009056 *catabolic process *and GO:0009116 *nucleoside metabolic process*. The intraerythrocytic cycle, composed of trophozite, ring, schizont and merozoite are highly interconnected. The merozoite stage presents a high number of associations with specific GO terms such as GO:0030260 *entry into host cell *and GO:0044409 *entry into host*. Trophozoites are associated with a small number of GO terms, including GO:0050896 *response to stimulus*, GO:0006096 *glycolysis*, GO:0006006 *glucose metabolic process *and GO:0006091 *generation of precursor metabolites and energy*. The stages schizont and ring are connected through the GO terms GO:0006955 *immune response*, GO:0050776 *regulation of immune response*, GO:0006325 *chromatin organization *and GO:0006091 *generation of precursor metabolites and energy*. It is also interesting to see that merozoits and schizonts are linked only through the GO term GO:0009116 *nucleoside metabolic process*.

#### Relative proportions of common and methods-specific results

In the case of CIA, one can observe a high overlap between the common results and the CIA specific GO terms associations: 8 GO terms (GO:0005975 *carbohydrate metabolic process*, GO:0006644 *phospholipid metabolic process*, GO:0008654 *phospholipid biosynthetic process*, GO:0045017 *glycerolipid biosynthetic process*, GO:0006661 *phosphatidylinositol biosynthetic process*, GO:0046488 *phosphatidylinositol metabolic process*, GO:0006506 *GPI anchor biosynthetic process*, GO:0016255 *attachment of GPI anchor to protein*) associated by CIA with merozoites in protein space, 8 GO terms (GO:0009058 *biosynthetic process*, GO:0051276 *chromosome organization*, GO:0006325 *chromatin organization*, GO:0050776 *regulation of immune response*, GO:0006955 *immune response*, GO:0006096 *glycolysis*, GO:0006334 *nucleosome assembly*, GO:0044237 *cellular metabolic process*) associated by CIA with rings and schizonts in protein space and 3 GO terms (GO:0009117 *nucleotide metabolic process*, GO:0006163 *purine nucleotide metabolic process*, GO:0009116 *nucleoside metabolic process*) associated by CIA with sporozoites in protein space. Only two GO terms (GO:0006096 *glycolysis *and GO:0006006 *glucose metabolic process associated with trophozoites*) from gene space, coincide with GO terms from the common results. Protein activity characteristics derived from CIA show considerable similarities to the other two methods.

Six specific results of GSVD for the life cycle stage ring coincide with the common GO terms associations with this stage (GO:0009058 *biosynthetic process*, GO:0019538 *protein metabolic process*, GO:0044237 *cellular metabolic process*, GO:0008152 *metabolic process*, GO:0055114 *oxidation-reduction process*, GO:0006091 *generation of precursor metabolites and energy*). There are three identical associations for the stage merozoite (GO:0019538 *protein metabolic process*, GO:0016311 *dephosphorylation *and GO:0006470 *protein dephosphorylation*). For each of the other stages, only one GO term from the common associations coincides with the method-specific associations (GO:0009987 *cellular process *for gametocytes, GO:0006091 *generation of precursor metabolites and energy *for trophozoites, GO:0020033 *antigenic variation *for schizonts and GO:0009056 *catabolic process *for sporozoites). In conclusion, the ring stage is very well characterized by the GSVD, which is almost in complete agreement with the other methods. The properties of the other stages do not coincide with the common results but should definitely be considered for further analysis as they are highly significant.

In this study, we have applied three integrative analysis methods to a data set containing mRNA and protein abundances from the six life cycle stages of *P. falciparum*. The use of integrative analysis methods allows to consider all annotated and measured genes (3283) and proteins (2491), not limited by the 2230 pairs of genes and proteins as when it was first published in [9]. The integration of knowledge on different levels allows the linking of the data sets based on samples and not on variables (genes, protein).

We presented three different integrative analysis methods, each with its own justification: CIA discovers biological processes on the basis of maximal covariance. GSVD decomposes the data sets into genelets and arraylets and conducts a modified GSE analysis on them. IBC computes biclusters according to the distance between genes, proteins and GO terms.

We have shown method-specific results as well as results common to all three analysis methods. In the case of CIA, the associations in protein space presented a high overlap with the common results. This was not the case for the associations in gene space. In case of the sporozoite stage, GSVD associations are very simmilar to the common results. For the other stages, GSVD yielded different mappings compared to the common results. As a GO term is associated or not with a life cycle stage, only general but no method-specific results were computed for IBC.

For CIA, it is important to consider that GO term associations are done through projection, whereas GSVD maps GO terms to individual stages through restricted GSE analysis and IBC assigns GO terms to life cycle stages through the distance to the corresponding life cycle stage. Another important aspect is that with CIA it is not possible to associate one GO term to more than one life cycle stage, while this is possible with GSVD and IBC. Due to the heterogeneous computational methods, we proposed taking the intersect of the three obtained results.

In the three-fold validated network view of the biological processes (Figure 9), we observe the separation of the intraerytrocytic cycle (merozite, ring, trophozoite and schizont) from sporozoites and gametocytes. While the stages of the intraerytrocytic cycle are tightly connected to one another, sporozoites share two biological processes and gametocytes share only one biological process with the rest. Gametocytes and sporozoites do not possess any common processes, reflecting the differences between these stages. Gametocytes are released into the blood stream, from where they travel to the liver, while sporozoites represent the sexual stage and lie dormant in cell cycle arrest until ingestion by a mosquito.

The data used here was gathered in order to investigate the role of post-transcriptional regulation in *P. falciparum *[9]. For this, only pairs of mRNA and the corresponding protein were considered, resulting in the exploitation of 89% of the proteins and 60% of the genes that were experimentally measured. By employing integrative analysis methods we were able to take all measured data into account.

LeRoch and coworkers [9] mention that there is a "bias in proteomic analysis of whole-cell lysates, in that such methods may fail to detect secreted or membrane proteins present in low abundance" such as GPI anchors. Due to the integrative approach, our analysis associates several GO terms related to GPI anchors proteins (GO:0006506 *GPI anchor biosynthetic process*, GO:0016255 *attachment of GPI anchor to protein*, GO:0006661 *phosphatidylinositol biosynthetic process*, GO:0046488 *phosphatidylinositol metabolic process*) with the merozoite stage, prevailing over this shortcoming. These associations are in agreement with [66], where distinct protein classes, with a focus on merozoite surface antigens, are discussed. The importance of GPI anchor proteins in the merozoite stage is well known and very important in immune evasion [67,68].

Other biological processes mentioned in [9] such as *glycolysis *and *cell invasion*, without any life cycle mapping, were also found in our network: GO:0044409 *entry into host *and GO:0030260 *entry into host cell*, both associated with the merozoite stage. Our network assigns GO:0006096 *glycolysis *to the stage trophozoite, in concordance to [69] where the transcriptome of *P. falciparum *was characterized.

Simmilar to our findings, cell invasion was associated with merozoites in [67], where a proteomic view of the *P. falciparum *life cycle was presented. Other concordances with [67] include the assignment of GO:0006508 *proteolysis *to the merozoite stage. During trophozoite stage, digestion of haemoglobin takes place. Our network maps GO:0006091 *generation of precursor metabolites and energy *to trophozoites, confirming the importance of energy production during this stage. As mentioned by Florens *et al*. [67], sporozoites are injected into the blood stream where they have to survive in a hostile environment. Based on our combined results, sporozoites are associated with GO:0020013 *modulation by symbiont of host erythrocyte aggregation *and GO:0020035 *cytoadherence to microvasculature, mediated by symbiont protein*, which reflects the process of survival. Additionally, sporozoites are associated with metabolism and transcription, as was shown in Figure 5 of [67]. Our results reflect these findings by mapping GO:0006163 *purine nucleotide metabolic process*, GO:0009117 *nucleotide metabolic process*, GO:0006351 *transcription, DNA dependent *and GO:0006355 *regulation of transcription, DNA dependent *to the sporozoite stage.

During gametocyte stage, DNA processing and energy production is highly regulated, as mentioned in [67]. In agreement, our results assign GO:0006323 *DNA packaging*, GO:0006839 *mitochondrial transport *and GO:0006626 *protein targeting to mitochondrion *to the gametocytes.

The analysis of the *P. falciparum *proteome by LaCount and colleagues [70] associated the intraerythrocytic cycle with chromatin modification, transcriptional regulation, mRNA stability/processing, ubiquitination, nucleic acid metabolism and invasion of host cells. Since our analysis corresponds to individual life cycle stages, we can associate biological processes to a certain stage of the intraerythrocytic cycle, providing a more detailed description of *P. falciparum*. According to our findings, chromatin modification takes place during schizont stage (GO:0006325 *chromatin organization*, GO:0051276 *chromosome organization*); merozoites are associated with GO:0006357 *regulation of transcription from RNA polymerase II promotor *and schizonts with GO:0042795 *transcription from RNA polymerase II promotor *; merozoites are associated with GO:0009116 *nucleoside metabolic process*; invasion of host cells can be observed during merozoite stage (GO:0044409 *entry into host *and GO:0030260 *entry into host cell*). Ubiquitination was only detected through its parent term GO:0044267 *cellular protein metabolic process*, which was associated with merozoites.

Fagan *et al*. [1] conducted CIA on a slightly different data set which took *P. berghei *orthologues into account and showed that GO:0006412 *biosynthesis *is associated to the intraerythrocytic cycle. In our network, several more specialized biosynthetic processes are associated with the merozoite stage: GO:0009059 *macromolecule biosynthetic process*, GO:0008654 *phospholipid biosynthetic process*, GO:0045017 *glycerolipid biosynthetic process*, GO:0006661 *phosphatidylinositol biosynthetic process*, GO:0006506 *GPI anchor biosynthetic process*, as well as the GO term GO:0006412 *biosynthetic process *itself.

The importance of immune evasion through antigenic variation was highlighted by Winzeler [71]. Our results show that this process is related to the schizont stage, as our analysis associates GO:0020033 *antigenic variation*, GO:0006955 *immune response*, GO:0050776 *regulation of immune response*, GO:0002377 *immunoglobulin production*, GO:0006950 *response to stress *and GO:0009607 *response to biotic stimulus *with this stage.

The role of lipids during merozoite stage was already shown in 1988 by Mikkelsen *et al*. [72]. Our computed network associates merozoites with GO:0006644 *phospholipid metabolic process*, GO:0008654 *phospholipid biosynthetic process*, GO:0046486 *glycerolipid metabolic process *and GO:0006629 *lipid metabolic process*, reflecting this early finding.

Phosphorilation and dephosphoryliation processes play an important role in the internalization step of meroziotes [73], a fact that is also reflected by our results. Merozoites are associated with GO:0016311 *dephosphorylation *and GO:0006470 *protein dephosphorylation*.

The role of the pentose phosphate pathway in *P. falciparum *was disscused in [74], without a clear life cycle stage assignment. Our computed network view maps GO:0006098 *pentose-phosphate shunt *to merozoites.

As shown in [75], REDOX complexes play an important role during ring stage, which is in agreement with our results that associate ring stage with GO:0045454 *cell redox homeostasis *and GO:0055114 *oxidation-reduction process*.

Roth [76] showed that carbohydrate metabolism is a key metabolic process connecting the host cells with *P*. *falciparum*. Our findings assign GO:0005975 *carbohydrate metabolic process *to merozoite and ring stages.

Most of our network associations are in concordance with several publications dealing with the characterization of *P. falciparum*, based on transcriptome [68,69] and proteome [67,70] characterization data. A considerable amount of the findings in the above publications are concentrated in our results of the used integrative analysis methods. Our findings are more detailed through the association with a specific life cycle stage rather than, e.g. the whole intraerythrocytic cycle as well as through the association of a child GO term instead of a parent GO term to the corresponding stage. Our study unifies individual findings from several publications of the past 25 years of research. Not all results from the publications mentioned above are present in our network. This could be due to the fact that none of the cited publications, except [9], used the same data sets as we did. Llinas *et al*. [68] compared the three *P. falciparum *strains 3D7, Dd2 and HB3 through the measurement of the gene expression profiles of 6287, 5294 and 6415 genes during the intraerythrocytic cycle. Bozdech *et al*. [69] considered in their analysis of the intraerythrocytic cycle transcriptome the expression of 5508 genes. LaCount and colleagues [70] analysed 1267 proteins for their protein interaction network of *P. falciparum*. In [67], Florens *et al*. use approximately 2400 proteins in order to create a proteomic view of the *P. falciparum *life cycle. The other studies are based on lab experiments on smaller groups of genes or proteins [66,71-73,75].

Additionally, our combined network view of life cycle stage dependent GO term association provides a new overview for the vaccine research and offers new insight in the interdependencies between life cycle stages. Possibly it could even identify key biological processes on which vaccine researchers could concentrate their work.

## Conclusion

In this study we have shown the power of integrative analysis methods. We presented three very different approaches that showed significant overlap of results. We compared our findings against the past 25 years of *P. falciparum *research and showed that the obtained network unifies, on the life cycle level, results from analyses done separately on transcriptome and proteome data, as well as results from the lab, which were performed on small groups of genes or proteins. Further investigations are needed to obtain a complete map of the biological processes activated during the life cycle of *P. falciparum*. Measurement of the transcriptome and proteome of *P. falciparum*, exploiting the advantages of current high throughput technologies, would complement the spectrum of biological process presented here. An increase of our understanding of *P. falciparum *could be achieved by performing the integrative analysis methods on the molecular function and/or cellular compartment level of gene ontology. Further work could also cover the identfication of genes and proteins that play key roles during the life cycle of *P. falciparum *through integrative analysis on gene and protein level, not only on GO term level.

## Competing interests

The authors declare that they have no competing interests.

## Authors' contributions

GGT and DM conceived the original idea. OAT performed the analysis, summarized and compared the results and drafted the manuscript under the supervision of GGT. All the authors have read and approved the final manuscript.

## Supplementary Material

Additional file 1 **GO term mapping**. PDF file containing the mapping between the numbers used in the CIA plots, GO terms and GO names. Click here for file

Additional file 2 **CIA general GO term associations in gene space**. PDF file containing the CIA general GO term associations in gene space. Click here for file

Additional file 3 **CIA general GO term associations in gene space**. PDF file containing the CIA general GO term associations in protein space. Click here for file

Additional file 4 **CIA division limits**. PDF file containing the CIA division limits for general (left) and specific (right) associations. The colours of the areas correspond to the colours of the stages they are associated with. Click here for file

Additional file 5 **CIA specific GO term associations in gene space**. PDF file containing the CIA specific GO term associations in gene space. Click here for file

Additional file 6 **CIA specific GO term associations in protein space**. PDF file containing the CIA specific GO term associations in protein space. Click here for file

Additional file 7 **GSVD general GO term associations**. PDF file containing the GSVD based general associations of GO terms to life cycle stages in common space. Click here for file

Additional file 8 **GSVD specific GO term associations**. PDF file containing the GSVD based specific associations of GO terms to life cycle stages in common space. Click here for file

Additional file 9 **IBC general GO term associations**. PDF file containing the IBC based general associations of GO terms to life cycle stages in common space. Click here for file

## References

[B1] Fagan A Culhane AC Higgins DG A multivariate analysis approach to the integration of proteomic and gene expression data Proteomics 2007 7 2162 2171 10.1002/pmic.200600898 17549791

[B2] Lazar C Taminau J Meganck S Steenhoff D Coletta A Weiss Solis DY Molter C Duque R Bersini H Nowe A GENESHIFT: a Non-Parametric Approach for Integrating Microarray Gene Expression Data Based on the Inner Product as a Distance Measure Between the Distributions of Genes IEEE/ACM Trans Comput Biol Bioinf 2013 2 383 292 23929862 10.1109/TCBB.2013.12

[B3] Alter O Brown PO Botstein D Generalized singular value decomposition for comparative analysis of genome-scale expression data sets of two different organisms Proc Natl Acad Sci USA 2003 100 3351 3356 10.1073/pnas.0530258100 12631705 PMC152296

[B4] Wang KS Liu X Integrative Analysis of Genome-wide Expression and Methylation Data J Biom Biostat 2013 4 4 6

[B5] Mo Q Wang S Seshan VE Olshen AB Schultz N Sander C Powers RS Ladanyi M Shen R Pattern discovery and cancer gene identification in integrated cancer genomic data Proc Natl Acad Sci USA 2013 110 4245 4250 10.1073/pnas.1208949110 23431203 PMC3600490

[B6] Kockmann T Gerstung M Schlumpf T Xhinzhou Z Hess D Beerenwinkel N Beisel C Paro R The BET protein FSH functionally interacts with ASH1 to orchestrate global gene activity in Drosophila Genome Biol 2013 14 R18 10.1186/gb-2013-14-2-r18 23442797 PMC4053998

[B7] Chen Z Zhang W Integrative Analysis Using Module-Guided Random Forests Reveals Correlated Genetic Factors Related to Mouse Weight PLoS Comput Biol 2013 9 e1002956 10.1371/journal.pcbi.1002956 23505362 PMC3591263

[B8] Gerstein MB Kundaje A Hariharan M Landt SG Yan KK Cheng C Mu XJ Khurana E Rozowsky J Alexander R Min R Alves P Abyzov A Addleman N Bhardwaj N Boyle AP Cayting P Charos A Chen DZ Cheng Y Clarke D Eastman C Euskirchen G Frietze S Fu Y Gertz J Grubert F Harmanci A Jain P Kasowski M Lacroute P Leng J Lian J Monahan H O'Geen H Ouyang Z Partridge EC Patacsil D Pauli F Raha D Ramirez L Reddy TE Reed B Shi M Slifer T Wang J Wu L Yang X Yip KY Zilberman-Schapira G Batzoglou S Sidow A Farnham PJ Myers RM Weissman SM Snyder M Architecture of the human regulatory network derived from ENCODE data Nature 2012 489 91 100 10.1038/nature11245 22955619 PMC4154057

[B9] Le Roch KG Johnson JR Florens L Zhou Y Santrosyan A Grainger M Yan SF Williamson KC Holder A Carucci DJ Yates JR Winzeler E Global analysis of transcript and protein levels across the Plasmodium falciparum life cycle Genome Res 2004 14 2308 2318 10.1101/gr.2523904 15520293 PMC525690

[B10] Cox B Kislinger T Emili A Integrating gene and protein expression data: pattern analysis and profile mining Methods 2005 35 303 314 10.1016/j.ymeth.2004.08.021 15722226

[B11] Cagney G Park S Chung C Tong B Dushlaine CO Shields DC Emili A Human Tissue Profiling with Multidimensional Protein Identification Technology J Proteome Res 2005 4 1757 1767 10.1021/pr0500354 16212430

[B12] Corbin RW Paliy O Yang F Shabanowitz J Platt M Lyons CE Root K McAuliffe J Jordan MI Kustu S Soupene E Hunt DF Toward a protein profile of Escherichia coli: comparison to its transcription profile Proc Natl Acad Sci USA 2003 100 16 9232 9237 10.1073/pnas.1533294100 12878731 PMC170901

[B13] Chen Yr Juan Hf Huang Hc Huang Hh Lee Yj Liao My Tseng Cw Lin Ll Chen Jy Wang Mj Chen Jh Chen Yj Quantitative Proteomic and Genomic Profiling Reveals Metastasis-Related Protein Expressio Patterns in Gastric Cancer Cells research articles J Proteome Res 2006 5 2727 2742 10.1021/pr060212g 17022644

[B14] Griffin TJ Complementary Profiling of Gene Expression at the Transcriptome and Proteome Levels in Saccharomyces cerevisiae Mol Cell Proteomics 2002 1 323 333 10.1074/mcp.M200001-MCP200 12096114

[B15] Mootha VK Bunkenborg J Olsen JV Hjerrild M Wisniewski JR Stahl E Bolouri MS Ray HN Sihag S Kamal M Patterson N Lander ES Mann M Integrated Analysis of Protein Composition, Tissue Diversity, and Gene Regulation in Mouse Mitochondria Cell 2003 115 629 640 10.1016/S0092-8674(03)00926-7 14651853

[B16] Washburn MP Koller A Oshiro G Ulaszek RR Plouffe D Deciu C Winzeler E Yates JR Protein pathway and complex clustering of correlated mRNA and protein expression analyses in Saccharomyces cerevisiae Proc Natl Acad Sci USA 2003 100 3107 3112 10.1073/pnas.0634629100 12626741 PMC152254

[B17] Kislinger T Cox B Kannan A Chung C Hu P Ignatchenko A Scott MS Gramolini AO Morris Q Hallett MT Rossant J Hughes TR Frey B Emili A Global Survey of Organ and Organelle Protein Expression in Mouse: Combined Proteomic and Transcriptomic Profiling Cell 2006 125 173 186 10.1016/j.cell.2006.01.044 16615898

[B18] Nie L Wu G Brockman FJ Zhang W Integrated analysis of transcriptomic and proteomic data of Desulfovibrio vulgaris: zero-inflated Poisson regression models to predict abundance of undetected proteins Bioinformatics 2006 22 1641 1647 10.1093/bioinformatics/btl134 16675466

[B19] Haider S Pal R Integrated Analysis of Transcriptomic and Proteomic Data Curr Genomics 2013 14 91 110 10.2174/1389202911314020003 24082820 PMC3637682

[B20] Hwang D Smith JJ Leslie DM Weston AD Rust AG Ramsey S de Atauri P Siegel AF Bolouri H Aitchison JD Hood L A data integration methodology for systems biology: Experimental verification Proc Natl Acad Sci USA 2005 102 17302 17307 10.1073/pnas.0508649102 16301536 PMC1297683

[B21] Hwang D Rust AG Ramsey S Smith JJ Leslie DM Weston AD de Atauri P Aitchison JD Hood L Siegel AF Bolouri H A data integration methodology for systems biology Proc Natl Acad Sci USA 2005 102 17296 17301 10.1073/pnas.0508647102 16301537 PMC1297682

[B22] Hahne H Mäder U Otto A Bonn F Steil L Bremer E Hecker M Becher D A comprehensive proteomics and transcriptomics analysis of Bacillus subtilis salt stress adaptation J Bacteriol 2010 192 870 882 10.1128/JB.01106-09 19948795 PMC2812467

[B23] Verhoef S Ballerstedt H Volkers RJM de Winde JH Ruijssenaars HJ Comparative transcriptomics and proteomics of p-hydroxybenzoate producing Pseudomonas putida S12: novel responses and implications for strain improvement Appl Microbiol Biotechnol 2010 87 679 690 10.1007/s00253-010-2626-z 20449741 PMC2874742

[B24] Takemasa I Kittaka N Hitora T Watanabe M Matsuo EI Mizushima T Ikeda M Yamamoto H Sekimoto M Nishimura O Doki Y Mori M Potential biological insights revealed by an integrated assessment of proteomic and transcriptomic data in human colorectal cancer Int J Oncol 2012 40 551 559 22025299 10.3892/ijo.2011.1244

[B25] Piruzian E Bruskin S Ishkin A Abdeev R Moshkovskii S Melnik S Nikolsky Y Nikolskaya T Integrated network analysis of transcriptomic and proteomic data in psoriasis BMC Syst Biol 2010 4 41 53 10.1186/1752-0509-4-41 20377895 PMC2873316

[B26] Perco P Mühlberger I Mayer G Oberbauer R Lukas A Mayer B Linking transcriptomics and proteomic data on the level of protein interaction networks Electrophoresis 2010 31 1780 1789 10.1002/elps.200900775 20432478

[B27] Joyce AR Palsson BO The model organism as a system: integrating 'omics' data sets Nat Rev Mol Cell Biol 2006 7 198 210 10.1038/nrm1857 16496022

[B28] Hecker M Lambeck S Toepfer S van Someren E Guthke R Gene regulatory network inference: data integration in dynamic models-a review BioSystems 2009 96 86 103 10.1016/j.biosystems.2008.12.004 19150482

[B29] Zhang W Li F Nie L Integrating multiple 'omics' analysis for microbial biology: application and methodologies Microbiology 2010 156 287 301 10.1099/mic.0.034793-0 19910409

[B30] Dolèdec S Chessel D Co-inertia analysis: an alternative method for studying species-environment relationships Freshw Biol 1994 31 277 294 10.1111/j.1365-2427.1994.tb01741.x

[B31] Kaiser S Biclustering: Methods, Software and Application PhD thesis 2011 Ludwig-Maximilians-University Munich, Department of Statistics

[B32] Ashburner M Ball CA Blake JA Botstein D Butler H Cherry JM Davis AP Dolinski K Dwight SS Eppig JT Harris MA Hill DP Issel-tarver L Kasarskis A Lewis S Matese JC Richardson JE Rubin GM Sherlock G Gene Ontology: tool for the unification of biology Nat Genet 2000 25 25 29 10.1038/75556 10802651 PMC3037419

[B33] Le Roch KG Zhou Y Blair PL Grainger M Moch JK Haynes JD De La Vega P Holder A Batalov S Carucci DJ Winzeler E Discovery of gene function by expression profiling of the malaria parasite life cycle Science 2003 301 1503 1508 10.1126/science.1087025 12893887

[B34] R Development Core Team R: A Language and Environment for Statistical Computing 2013 R Foundation for Statistical Computing, Vienna, Austria http://www.R-project.org [ISBN 3-900051-07-0]

[B35] Carlson M org.Pf.plasmo.db: Genome wide annotation for Malaria [R package version 2.8.1]

[B36] Carlson M GO.db: A set of annotation maps describing the entire Gene Ontology [R package version 2.8.0]

[B37] Tucker LR An inter-battery method for factor analysis Psychometrika 1958 23 111 136 10.1007/BF02289009

[B38] Jarraud S Mougel C Thioulouse J Lina G Meugnier H Forey F Etienne J Vandenesch F Jarraud S Mougel C Thioulouse J Lina G Nesme X Etienne J Relationships between Staphylococcus aureus Genetic Background, Virulence Factors, agr Groups (Alleles), and Human Disease Infect Immun 2002 70 631 641 10.1128/IAI.70.2.631-641.2002 11796592 PMC127674

[B39] Thioulouse J Lobry J Co-inertia analysis of amino-acid physico-chemical properties and protein composition with the ADE package Comput Appl Biosci 1995 11 321 329 7583702 10.1093/bioinformatics/11.3.321

[B40] Culhane AC Perrière G Higgins DG Cross-platform comparison and visualisation of gene expression data using co-inertia analysis BMC Bioinformatics 2003 4 59 10.1186/1471-2105-4-59 14633289 PMC317282

[B41] Jolliffe IT Principal Component Analysis 2002 New York Berlin Heidelberg: Springer-Verlag

[B42] Gimaret-Carpentier C Chessel D Pascal J Non-symmetric correspondence analysis: an alternative for species occurrences data Plant Ecol 1998 138 97 112 10.1023/A:1009708824434

[B43] Greenacre M Theory and Applications of Correspondence Analysis 1983 London: Academic Press

[B44] Robert P Escoufier Y A Unifying Tool for Linear Multivariate Statistical Methods: The RV-Coefficient Appl Statist 1976 25 257 265 10.2307/2347233

[B45] Culhane AC Thioulouse J Perrière G Higgins DG MADE4: an R package for multivariate analysis of gene expression data Bioinformatics 2005 21 2789 2790 10.1093/bioinformatics/bti394 15797915

[B46] Chessel D Dufour AB Thioulouse J The ade4 package - I: One table methods R News 2004 5 5 10

[B47] Busold CH Winter S Hauser N Bauer A Dippon J Hoheisel JD Fellenberg K Integration of GO annotations in Correspondence Analysis: facilitating the interpretation of microarray data Bioinformatics 2005 21 2424 2429 10.1093/bioinformatics/bti367 15746280

[B48] Alter O Brown PO Botstein D Singular value decomposition for genome-wide expression data processing and modeling Proc Natl Acad Sci USA 2000 97 10101 10106 10.1073/pnas.97.18.10101 10963673 PMC27718

[B49] Nielsen TO West RB Linn SC Alter O Knowling MA Connell JXO Zhu S Fero M Sherlock G Pollack JR Brown PO Botstein D Rijn MVD Mechanisms of disease Molecular characterisation of soft tissue tumours: a gene expression study The Lancet 2002 359 1301 1307 10.1016/S0140-6736(02)08270-3 11965276

[B50] Wen X Fuhrman S Michaels GS Carr DB Smith S Barker JL Somogy R Large-scale temporal gene expression mapping of central nevous system development Proc Natl Acad Sci USA 1998 95 334 339 10.1073/pnas.95.1.334 9419376 PMC18216

[B51] Hilsenbeck SG William E Schiff R Connell O Hansen RK Osborne K Fuqua SAW Statistical Analysis of Array Expression Data as Applied to the Problem of Tamoxifen Resistance J Natl Cancer Inst 1999 91 453 459 10.1093/jnci/91.5.453 10070945

[B52] Golub GH Van Loan CF Matrix Computation 1996 Baltimore and London: Johns Hopkins University Press

[B53] Paige CC Saunders MA Towards a Generalized Singular Value Decomposition SIAM J Number Anal 1981 18 398 405 10.1137/0718026

[B54] Falcon S Gentleman R Using GOstats to test gene lists for GO term association Bioinformatics 2007 23 257 258 10.1093/bioinformatics/btl567 17098774

[B55] Hartigan JA Direct Clustering of a Data Matrix J Am Stat Assoc 1972 67 123 129 10.1080/01621459.1972.10481214

[B56] Cheng Y Church M Biclustering of Expression Data Proc Int Conf Intell Syst Mol Biol 2000 8 93 103 10977070

[B57] Getz G Levine E Domany E Coupled two-way clustering analysis of gene microarray data Proc Natl Acad Sci USA 2000 97 12079 12084 10.1073/pnas.210134797 11035779 PMC17297

[B58] Ben-Dor A Chor B Karp R Yukhini Z Descovering local structure in gene expression data: The order preserving submatrix problem J Comput Biol 2003 10 373 384 10.1089/10665270360688075 12935334

[B59] Murali T Kasif S Extracting conserved gene expression motifs from gene expression data Pac Symp Biocomput 2003 8 77 88 12603019

[B60] Madeira SC Oliveira AL Biclustering algorithms for biological data analysis: a survey IEEE/ACM Trans Comput Biol Bioinf 2004 1 24 45 10.1109/TCBB.2004.2 17048406

[B61] Prelić A Bleuler S Zimmermann P Wille A Bühlmann P Gruissem W Hennig L Thiele L Zitzler E A systematic comparison and evaluation of biclustering methods for gene expression data Bioinformatics 2006 22 1122 1129 10.1093/bioinformatics/btl060 16500941

[B62] Kaiser S Santamaria R Tatsiana Khamiakova Sill M Theron R Quintales L Leisch Fbiclust: BiCluster Algorithms 2013 http://CRAN.R-project.org/package=biclust [R package version 1.0.2]

[B63] Curtis C Shah SP Chin SF Turashvili G Rueda OM Dunning MJ Speed D Lynch AG Samarajiwa S Yuan Y Gräf S Ha G Haffari G Bashashati A Russell R McKinney S Langerød A Green A Provenzano E Wishart G Pinder S Watson P Markowetz F Murphy L Ellis I Purushotham A Børresen-Dale AL Brenton JD Tavaré S Caldas C Aparicio S The genomic and transcriptomic architecture of 2,000 breast tumours reveals novel subgroups Nature 2012 486 346 352 22522925 10.1038/nature10983 PMC3440846

[B64] Tanay A Sharan R Kupiec M Shamir R Revealing modularity and organization in the yeast molecular network by integrated analysis of highly heterogeneous genomewide data Proc Natl Acad Sci USA 2004 101 2981 2986 10.1073/pnas.0308661100 14973197 PMC365731

[B65] Smoot M Ono K Ruscheinski J Wang PL Ideker T Cytoscape 2.8: new features for data integration and network visualization Bioinformatics 2011 27 431 432 10.1093/bioinformatics/btq675 21149340 PMC3031041

[B66] Sanders PR Gilson PR Cantin GT Greenbaum DC Nebl T Carucci DJ McConville MJ Schofield L Hodder AN Yates JR Crabb BS Distinct protein classes including novel merozoite surface antigens in Raftlike membranes of Plasmodium falciparum J Biol Chem 2005 280 40169 40176 10.1074/jbc.M509631200 16203726

[B67] Florens L Washburn MP Raine JD Anthony RM Grainger M Haynes JD Moch JK Muster N Sacci JB Tabb DL Witney Aa Wolters D Wu Y Gardner MJ Holder Aa Sinden RE Yates JR Carucci DJ A proteomic view of the Plasmodium falciparum life cycle Nature 2002 419 520 526 10.1038/nature01107 12368866

[B68] Llinás M Bozdech Z Wong ED Adai AT DeRisi JL Comparative whole genome transcriptome analysis of three Plasmodium falciparum strains Nucleic Acids Res 2006 34 1166 1173 10.1093/nar/gkj517 16493140 PMC1380255

[B69] Bozdech Z Llinás M Pulliam BL Wong ED Zhu J DeRisi JL The transcriptome of the intraerythrocytic developmental cycle of Plasmodium falciparum PLoS Biol 2003 1 E5 12929205 10.1371/journal.pbio.0000005 PMC176545

[B70] LaCount DJ Vignali M Chettier R Phansalkar A Bell R Hesselberth JR Schoenfeld LW Ota I Sahasrabudhe S Kurschner C Fields S Hughes RE A protein interaction network of the malaria parasite Plasmodium falciparum Nature 2005 438 103 107 10.1038/nature04104 16267556

[B71] Winzeler EA Malaria research in the post-genomic era Nature 2008 455 751 756 10.1038/nature07361 18843360 PMC2705782

[B72] Mikkelsen RB Kamber M Wadwa KS Lin PS Schmidt-Ullrich R The role of lipids in Plasmodium falciparum invasion of erythrocytes: a coordinated biochemical and microscopic analysis Proc Natl Acad Sci USA 1988 85 5956 5960 10.1073/pnas.85.16.5956 3045809 PMC281884

[B73] Ward G Fujioka H Aikawa M Miller L Staurosporine Inhibits Invasion of Erythrocytes by Malarial Merozoites Exp Parasitol 1994 79 480 487 10.1006/expr.1994.1109 7957765

[B74] Bozdech Z Ginsburg H Data mining of the transcriptome of Plasmodium falciparum: the pentose phosphate pathway and ancillary processes Malaria J 2005 4 17 10.1186/1475-2875-4-17 15774020 PMC1084361

[B75] Mok S Imwong M Mackinnon MJ Sim J Ramadoss R Yi P Mayxay M Chotivanich K Liong KY Russell B Socheat D Newton PN Day NPJ White NJ Preiser PR Nosten F Dondorp AM Bozdech Z Artemisinin resistance in Plasmodium falciparum is associated with an altered temporal pattern of transcription BMC Genomics 2011 12 391 10.1186/1471-2164-12-391 21810278 PMC3163569

[B76] Roth EJ Plasmodium falciparum carbohydrate metabolism: a connection between host cell and parasite Blood Cells 1990 16 453 466 2257322

