# Supplementary material for: Integrative omics analysis. A study based on Plasmodium falciparum mRNA and protein data
Source: BMC Syst Biol. 2014 Mar 13;8(Suppl 2):S4. doi: 10.1186/1752-0509-8-S2-S4 (PMC4101701; doi:10.1186/1752-0509-8-S2-S4)
Supplement: Additional file 1 — GO term mapping. PDF file containing the mapping between the numbers used in the CIA plots, GO terms and GO names. [file 1752-0509-8-S2-S4-S1.pdf]

PDF file containing the mapping between the numbers used in the CIA plots, GO terms and GO names.

Table 1: Mapping between GO terms, GO names and the numbers used in CIA.

Number	GO term	GO name
1	GO:0009405	pathogenesis
2	GO:0016337	cell-cell adhesion
3	GO:0020013	modulation by symbiont of host erythrocyte aggregation
4	GO:0020033	antigenic variation
5	GO:0020035	cytoadherence to microvasculature, mediated by symbiont protein
6	GO:0042000	translocation of peptides or proteins into host
7	GO:0006457	protein folding
8	GO:0050776	regulation of immune response
9	GO:0030260	entry into host cell
10	GO:0006468	protein phosphorylation
11	GO:0006412	translation
12	GO:0006418	tRNA aminoacylation for protein translation
13	GO:0006422	aspartyl-tRNA aminoacylation
14	GO:0006887	exocytosis
15	GO:0015031	protein transport
16	GO:0006508	proteolysis
17	GO:0006817	phosphate ion transport
18	GO:0006810	transport
19	GO:0006511	ubiquitin-dependent protein catabolic process
20	GO:0019288	isopentenyl diphosphate biosynthetic process, mevalonate-independent pathway
21	GO:0055114	oxidation-reduction process
22	GO:0055085	transmembrane transport
23	GO:0007010	cytoskeleton organization
24	GO:0006364	rRNA processing
25	GO:0032259	methylation
26	GO:0007049	cell cycle
27	GO:0006139	nucleobase-containing compound metabolic process
28	GO:0015986	ATP synthesis coupled proton transport
29	GO:0015991	ATP hydrolysis coupled proton transport
30	GO:0006754	ATP biosynthetic process
31	GO:0006812	cation transport
32	GO:0006816	calcium ion transport
33	GO:0008152	metabolic process
34	GO:0006413	translational initiation
35	GO:0042254	ribosome biogenesis
36	GO:0006184	GTP catabolic process
37	GO:0006886	intracellular protein transport
38	GO:0006913	nucleocytoplasmic transport
39	GO:0007165	signal transduction
40	GO:0007264	small GTPase mediated signal transduction
41	GO:0060627	regulation of vesicle-mediated transport
42	GO:0008299	isoprenoid biosynthetic process
43	GO:0007067	mitosis
44	GO:0006414	translational elongation
45	GO:0006281	DNA repair
46	GO:0006302	double-strand break repair

Continued on next page

Table 1 – continued from previous page

Number	GO term	GO name
47	GO:0051603	proteolysis involved in cellular protein catabolic process
48	GO:0006260	DNA replication
49	GO:0009408	response to heat
50	GO:0006461	protein complex assembly
51	GO:0007021	tubulin complex assembly
52	GO:0006474	N-terminal protein amino acid acetylation
53	GO:0006355	regulation of transcription, DNA-dependent
54	GO:0035556	intracellular signal transduction
55	GO:0006432	phenylalanyl-tRNA aminoacylation
56	GO:0043039	tRNA aminoacylation
57	GO:0008283	cell proliferation
58	GO:0006366	transcription from RNA polymerase II promoter
59	GO:0046488	phosphatidylinositol metabolic process
60	GO:0048015	phosphatidylinositol-mediated signaling
61	GO:0006334	nucleosome assembly
62	GO:0006367	transcription initiation from RNA polymerase II promoter
63	GO:0007018	microtubule-based movement
64	GO:0007155	cell adhesion
65	GO:0006221	pyrimidine nucleotide biosynthetic process
66	GO:0006428	isoleucyl-tRNA aminoacylation
67	GO:0006417	regulation of translation
68	GO:0006259	DNA metabolic process
69	GO:0019835	cytolysis
70	GO:0019836	hemolysis by symbiont of host erythrocytes
71	GO:0006464	cellular protein modification process
72	GO:0016117	carotenoid biosynthetic process
73	GO:0006289	nucleotide-excision repair
74	GO:0007017	microtubule-based process
75	GO:0006520	cellular amino acid metabolic process
76	GO:0009058	biosynthetic process
77	GO:0006351	transcription, DNA-dependent
78	GO:0006353	DNA-dependent transcription, termination
79	GO:0006397	mRNA processing
80	GO:0008646	high-affinity hexose transport
81	GO:0045426	quinone cofactor biosynthetic process
82	GO:0044237	cellular metabolic process
83	GO:0042176	regulation of protein catabolic process
84	GO:0009073	aromatic amino acid family biosynthetic process
85	GO:0006383	transcription from RNA polymerase III promoter
86	GO:0006163	purine nucleotide metabolic process
87	GO:0006188	IMP biosynthetic process
88	GO:0009152	purine ribonucleotide biosynthetic process
89	GO:0016255	attachment of GPI anchor to protein
90	GO:0016226	iron-sulfur cluster assembly
91	GO:0002377	immunoglobulin production
92	GO:0009790	embryo development
93	GO:0030216	keratinocyte differentiation
94	GO:0006633	fatty acid biosynthetic process
95	GO:0006629	lipid metabolic process
96	GO:0006396	RNA processing

Continued on next page

Table 1 – continued from previous page

Number	GO term	GO name
97	GO:0016114	terpenoid biosynthetic process
98	GO:0006836	neurotransmitter transport
99	GO:0006605	protein targeting
100	GO:0043952	protein transport by the Sec complex
101	GO:0015718	monocarboxylic acid transport
102	GO:0016192	vesicle-mediated transport
103	GO:0031338	regulation of vesicle fusion
104	GO:0032889	regulation of vacuole fusion, non-autophagic
105	GO:0008610	lipid biosynthetic process
106	GO:0006421	asparaginyl-tRNA aminoacylation
107	GO:0001522	pseudouridine synthesis
108	GO:0009451	RNA modification
109	GO:0006415	translational termination
110	GO:0006449	regulation of translational termination
111	GO:0006855	drug transmembrane transport
112	GO:0030163	protein catabolic process
113	GO:0006986	response to unfolded protein
114	GO:0044267	cellular protein metabolic process
115	GO:0006888	ER to Golgi vesicle-mediated transport
116	GO:0044053	translocation of peptides or proteins into host cell cytoplasm
117	GO:0044409	entry into host
118	GO:0006631	fatty acid metabolic process
119	GO:0015909	long-chain fatty acid transport
120	GO:0006267	pre-replicative complex assembly
121	GO:0006338	chromatin remodeling
122	GO:0006904	vesicle docking involved in exocytosis
123	GO:0006869	lipid transport
124	GO:0006897	endocytosis
125	GO:0008203	cholesterol metabolic process
126	GO:0015992	proton transport
127	GO:0046034	ATP metabolic process
128	GO:0006974	response to DNA damage stimulus
129	GO:0000027	ribosomal large subunit assembly
130	GO:0000375	RNA splicing, via transesterification reactions
131	GO:0008380	RNA splicing
132	GO:0016042	lipid catabolic process
133	GO:0000398	nuclear mRNA splicing, via spliceosome
134	GO:0000154	rRNA modification
135	GO:0000059	protein import into nucleus, docking
136	GO:0018345	protein palmitoylation
137	GO:0000226	microtubule cytoskeleton organization
138	GO:0006467	protein thiol-disulfide exchange
139	GO:0045454	cell redox homeostasis
140	GO:0043687	post-translational protein modification
141	GO:0051246	regulation of protein metabolic process
142	GO:0017038	protein import
143	GO:0065002	intracellular protein transmembrane transport
144	GO:0006979	response to oxidative stress
145	GO:0006072	glycerol-3-phosphate metabolic process
146	GO:0006127	glycerophosphate shuttle

Continued on next page

Table 1 – continued from previous page

Number	GO term	GO name
147	GO:0006950	response to stress
148	GO:0016567	protein ubiquitination
149	GO:0006470	protein dephosphorylation
150	GO:0016311	dephosphorylation
151	GO:0046823	negative regulation of nucleocytoplasmic transport
152	GO:0060284	regulation of cell development
153	GO:0006529	asparagine biosynthetic process
154	GO:0006438	valyl-tRNA aminoacylation
155	GO:0006486	protein glycosylation
156	GO:0006487	protein N-linked glycosylation
157	GO:0018105	peptidyl-serine phosphorylation
158	GO:0010564	regulation of cell cycle process
159	GO:0030433	ER-associated protein catabolic process
160	GO:0032780	negative regulation of ATPase activity
161	GO:0051131	chaperone-mediated protein complex assembly
162	GO:0002312	B cell activation involved in immune response
163	GO:0006796	phosphate-containing compound metabolic process
164	GO:0051301	cell division
165	GO:0006378	mRNA polyadenylation
166	GO:0006379	mRNA cleavage
167	GO:0006096	glycolysis
168	GO:0015914	phospholipid transport
169	GO:0009966	regulation of signal transduction
170	GO:0043666	regulation of phosphoprotein phosphatase activity
171	GO:0006465	signal peptide processing
172	GO:0006333	chromatin assembly or disassembly
173	GO:0006488	dolichol-linked oligosaccharide biosynthetic process
174	GO:0007275	multicellular organismal development
175	GO:0006370	7-methylguanosine mRNA capping
176	GO:0019432	triglyceride biosynthetic process
177	GO:0032313	regulation of Rab GTPase activity
178	GO:0015908	fatty acid transport
179	GO:0016579	protein deubiquitination
180	GO:0000245	spliceosomal complex assembly
181	GO:0046907	intracellular transport
182	GO:0016070	RNA metabolic process
183	GO:0006554	lysine catabolic process
184	GO:0007035	vacuolar acidification
185	GO:0009438	methylglyoxal metabolic process
186	GO:0006744	ubiquinone biosynthetic process
187	GO:0009234	menaquinone biosynthetic process
188	GO:0006354	DNA-dependent transcription, elongation
189	GO:0008295	spermidine biosynthetic process
190	GO:0006261	DNA-dependent DNA replication
191	GO:0051052	regulation of DNA metabolic process
192	GO:0006400	tRNA modification
193	GO:0051276	chromosome organization
194	GO:0015684	ferrous iron transport
195	GO:0006606	protein import into nucleus
196	GO:0006811	ion transport

Continued on next page

Table 1 – continued from previous page

Number	GO term	GO name
197	GO:0006875	cellular metal ion homeostasis
198	GO:0046685	response to arsenic-containing substance
199	GO:0046939	nucleotide phosphorylation
200	GO:0006270	DNA-dependent DNA replication initiation
201	GO:0016568	chromatin modification
202	GO:0016480	negative regulation of transcription from RNA polymerase III promoter
203	GO:0017148	negative regulation of translation
204	GO:0006231	dTMP biosynthetic process
205	GO:0006545	glycine biosynthetic process
206	GO:0006730	one-carbon metabolic process
207	GO:0009165	nucleotide biosynthetic process
208	GO:0043248	proteasome assembly
209	GO:0006182	cGMP biosynthetic process
210	GO:0018144	RNA-protein covalent cross-linking
211	GO:0009059	macromolecule biosynthetic process
212	GO:0051258	protein polymerization
213	GO:0006446	regulation of translational initiation
214	GO:0006429	leucyl-tRNA aminoacylation
215	GO:0006626	protein targeting to mitochondrion
216	GO:0045039	protein import into mitochondrial inner membrane
217	GO:0016310	phosphorylation
218	GO:0030036	actin cytoskeleton organization
219	GO:0060327	cytoplasmic actin-based contraction involved in cell motility
220	GO:0006099	tricarboxylic acid cycle
221	GO:0008643	carbohydrate transport
222	GO:0006298	mismatch repair
223	GO:0019941	modification-dependent protein catabolic process
224	GO:0042787	protein ubiquitination involved in ubiquitin-dependent protein catabolic process
225	GO:0006437	tyrosyl-tRNA aminoacylation
226	GO:0000122	negative regulation of transcription from RNA polymerase II promoter
227	GO:0030154	cell differentiation
228	GO:0046323	glucose import
229	GO:0006323	DNA packaging
230	GO:0006360	transcription from RNA polymerase I promoter
231	GO:0006310	DNA recombination
232	GO:0046854	phosphatidylinositol phosphorylation
233	GO:0006265	DNA topological change
234	GO:0006021	inositol biosynthetic process
235	GO:0008654	phospholipid biosynthetic process
236	GO:0006750	glutathione biosynthetic process
237	GO:0009116	nucleoside metabolic process
238	GO:0000917	barrier septum assembly
239	GO:0009052	pentose-phosphate shunt, non-oxidative branch
240	GO:0008033	tRNA processing
241	GO:0015904	tetracycline transport
242	GO:0046677	response to antibiotic
243	GO:0009987	cellular process
244	GO:0051016	barbed-end actin filament capping
245	GO:0000079	regulation of cyclin-dependent protein kinase activity
246	GO:0045736	negative regulation of cyclin-dependent protein kinase activity

Continued on next page

Table 1 – continued from previous page

Number	GO term	GO name
247	GO:0051726	regulation of cell cycle
248	GO:0043487	regulation of RNA stability
249	GO:0006818	hydrogen transport
250	GO:0048034	heme O biosynthetic process
251	GO:0009228	thiamine biosynthetic process
252	GO:0008104	protein localization
253	GO:0042493	response to drug
254	GO:0007186	G-protein coupled receptor signaling pathway
255	GO:0006607	NLS-bearing substrate import into nucleus
256	GO:0006777	Mo-molybdopterin cofactor biosynthetic process
257	GO:0032312	regulation of ARF GTPase activity
258	GO:0043087	regulation of GTPase activity
259	GO:0009245	lipid A biosynthetic process
260	GO:0007169	transmembrane receptor protein tyrosine kinase signaling pathway
261	GO:0016043	cellular component organization
262	GO:0018344	protein geranylgeranylation
263	GO:0006207	'de novo' pyrimidine nucleobase biosynthetic process
264	GO:0006222	UMP biosynthetic process
265	GO:0051302	regulation of cell division
266	GO:0006165	nucleoside diphosphate phosphorylation
267	GO:0006183	GTP biosynthetic process
268	GO:0006228	UTP biosynthetic process
269	GO:0006241	CTP biosynthetic process
270	GO:0019368	fatty acid elongation, unsaturated fatty acid
271	GO:0006779	porphyrin-containing compound biosynthetic process
272	GO:0006783	heme biosynthetic process
273	GO:0006743	ubiquinone metabolic process
274	GO:0006591	ornithine metabolic process
275	GO:0030001	metal ion transport
276	GO:0044262	cellular carbohydrate metabolic process
277	GO:0006098	pentose-phosphate shunt
278	GO:0006357	regulation of transcription from RNA polymerase II promoter
279	GO:0034227	tRNA thio-modification
280	GO:0009306	protein secretion
281	GO:0006284	base-excision repair
282	GO:0006506	GPI anchor biosynthetic process
283	GO:0000304	response to singlet oxygen
284	GO:0008614	pyridoxine metabolic process
285	GO:0042819	vitamin B6 biosynthetic process
286	GO:0048870	cell motility
287	GO:0006090	pyruvate metabolic process
288	GO:0006820	anion transport
289	GO:0044070	regulation of anion transport
290	GO:0042128	nitrate assimilation
291	GO:0005975	carbohydrate metabolic process
292	GO:0009432	SOS response
293	GO:0006097	glyoxylate cycle
294	GO:0006352	DNA-dependent transcription, initiation
295	GO:0008615	pyridoxine biosynthetic process
296	GO:0042823	pyridoxal phosphate biosynthetic process

Continued on next page

Table 1 – continued from previous page

Number	GO term	GO name
297	GO:0043412	macromolecule modification
298	GO:0007034	vacuolar transport
299	GO:0042144	vacuole fusion, non-autophagic
300	GO:0006122	mitochondrial electron transport, ubiquinol to cytochrome c
301	GO:0045836	positive regulation of meiosis
302	GO:0006801	superoxide metabolic process
303	GO:0019430	removal of superoxide radicals
304	GO:0006401	RNA catabolic process
305	GO:0031123	RNA 3'-end processing
306	GO:0043631	RNA polyadenylation
307	GO:0051289	protein homotetramerization
308	GO:0006091	generation of precursor metabolites and energy
309	GO:0001682	tRNA 5'-leader removal
310	GO:0006729	tetrahydrobiopterin biosynthetic process
311	GO:0006424	glutamyl-tRNA aminoacylation
312	GO:0009435	NAD biosynthetic process
313	GO:0019357	nicotinate nucleotide biosynthetic process
314	GO:0019358	nicotinate nucleotide salvage
315	GO:0006865	amino acid transport
316	GO:0000082	G1/S transition of mitotic cell cycle
317	GO:0000105	histidine biosynthetic process
318	GO:0006164	purine nucleotide biosynthetic process
319	GO:0009086	methionine biosynthetic process
320	GO:0009396	folic acid-containing compound biosynthetic process
321	GO:0010501	RNA secondary structure unwinding
322	GO:0032508	DNA duplex unwinding
323	GO:0006814	sodium ion transport
324	GO:0006644	phospholipid metabolic process
325	GO:0007154	cell communication
326	GO:0015937	coenzyme A biosynthetic process
327	GO:0045173	O-sialoglycoprotein catabolic process
328	GO:0006825	copper ion transport
329	GO:0006878	cellular copper ion homeostasis
330	GO:0008535	respiratory chain complex IV assembly
331	GO:0030261	chromosome condensation
332	GO:0006829	zinc ion transport
333	GO:0006534	cysteine metabolic process
334	GO:0008616	queuosine biosynthetic process
335	GO:0006434	seryl-tRNA aminoacylation
336	GO:0030522	intracellular receptor mediated signaling pathway
337	GO:0006458	'de novo' protein folding
338	GO:0000280	nuclear division
339	GO:0042779	tRNA 3'-trailer cleavage
340	GO:0051028	mRNA transport
341	GO:0009607	response to biotic stimulus
342	GO:0042113	B cell activation
343	GO:0007530	sex determination
344	GO:0006306	DNA methylation
345	GO:0006614	SRP-dependent cotranslational protein targeting to membrane
346	GO:0045900	negative regulation of translational elongation

Continued on next page

Table 1 – continued from previous page

Number	GO term	GO name
347	GO:0000256	allantoin catabolic process
348	GO:0006144	purine nucleobase metabolic process
349	GO:0000055	ribosomal large subunit export from nucleus
350	GO:0042273	ribosomal large subunit biogenesis
351	GO:0006536	glutamate metabolic process
352	GO:0009190	cyclic nucleotide biosynthetic process
353	GO:0000724	double-strand break repair via homologous recombination
354	GO:0030259	lipid glycosylation
355	GO:0006890	retrograde vesicle-mediated transport, Golgi to ER
356	GO:0006761	dihydrofolate biosynthetic process
357	GO:0042558	pteridine-containing compound metabolic process
358	GO:0006004	fucose metabolic process
359	GO:0019673	GDP-mannose metabolic process
360	GO:0006086	acetyl-CoA biosynthetic process from pyruvate
361	GO:0019538	protein metabolic process
362	GO:0007131	reciprocal meiotic recombination
363	GO:0002720	positive regulation of cytokine production involved in immune response
364	GO:0006359	regulation of transcription from RNA polymerase III promoter
365	GO:0006655	phosphatidylglycerol biosynthetic process
366	GO:0006103	2-oxoglutarate metabolic process
367	GO:0009107	lipoate biosynthetic process
368	GO:0006835	dicarboxylic acid transport
369	GO:0006839	mitochondrial transport
370	GO:0015742	alpha-ketoglutarate transport
371	GO:0015743	malate transport
372	GO:0015858	nucleoside transport
373	GO:0007059	chromosome segregation
374	GO:0051205	protein insertion into membrane
375	GO:0042127	regulation of cell proliferation
376	GO:0051262	protein tetramerization
377	GO:0006525	arginine metabolic process
378	GO:0031365	N-terminal protein amino acid modification
379	GO:0043686	co-translational protein modification
380	GO:0006308	DNA catabolic process
381	GO:0031167	rRNA methylation
382	GO:0007219	Notch signaling pathway
383	GO:0018342	protein prenylation
384	GO:0006269	DNA replication, synthesis of RNA primer
385	GO:0006420	arginyl-tRNA aminoacylation
386	GO:0046836	glycolipid transport
387	GO:0018279	protein N-linked glycosylation via asparagine
388	GO:0006556	S-adenosylmethionine biosynthetic process
389	GO:0006541	glutamine metabolic process
390	GO:0008153	para-aminobenzoic acid biosynthetic process
391	GO:0006542	glutamine biosynthetic process
392	GO:0006807	nitrogen compound metabolic process
393	GO:0006772	thiamine metabolic process
394	GO:0009229	thiamine diphosphate biosynthetic process
395	GO:0016575	histone deacetylation
396	GO:0007205	protein kinase C-activating G-protein coupled receptor signaling pathway

Continued on next page

Table 1 – continued from previous page

Number	GO term	GO name
397	GO:0007020	microtubule nucleation
398	GO:0015074	DNA integration
399	GO:0032196	transposition
400	GO:0006427	histidyl-tRNA aminoacylation
401	GO:0006431	methionyl-tRNA aminoacylation
402	GO:0000184	nuclear-transcribed mRNA catabolic process, nonsense-mediated decay
403	GO:0007266	Rho protein signal transduction
404	GO:0017183	peptidyl-diphthamide biosynthetic process from peptidyl-histidine
405	GO:0006493	protein O-linked glycosylation
406	GO:0006665	sphingolipid metabolic process
407	GO:0043161	proteasomal ubiquitin-dependent protein catabolic process
408	GO:0006788	heme oxidation
409	GO:0006166	purine ribonucleoside salvage
410	GO:0006177	GMP biosynthetic process
411	GO:0006914	autophagy
412	GO:0009225	nucleotide-sugar metabolic process
413	GO:0006423	cysteinyl-tRNA aminoacylation
414	GO:0042026	protein refolding
415	GO:0009186	deoxyribonucleoside diphosphate metabolic process
416	GO:0000045	autophagic vacuole assembly
417	GO:0002253	activation of immune response
418	GO:0006094	gluconeogenesis
419	GO:0007030	Golgi organization
420	GO:0009298	GDP-mannose biosynthetic process
421	GO:0019307	mannose biosynthetic process
422	GO:0006471	protein ADP-ribosylation
423	GO:0009264	deoxyribonucleotide catabolic process
424	GO:0042773	ATP synthesis coupled electron transport
425	GO:0006101	citrate metabolic process
426	GO:0006041	glucosamine metabolic process
427	GO:0016051	carbohydrate biosynthetic process
428	GO:0019353	protoporphyrinogen IX biosynthetic process from glutamate
429	GO:0009168	purine ribonucleoside monophosphate biosynthetic process
430	GO:0045893	positive regulation of transcription, DNA-dependent
431	GO:0006596	polyamine biosynthetic process
432	GO:0006597	spermine biosynthetic process
433	GO:0009445	putrescine metabolic process
434	GO:0022900	electron transport chain
435	GO:0006898	receptor-mediated endocytosis
436	GO:0007276	gamete generation
437	GO:0022904	respiratory electron transport chain
438	GO:0016539	intein-mediated protein splicing
439	GO:0019478	D-amino acid catabolic process
440	GO:0046777	protein autophosphorylation
441	GO:0006104	succinyl-CoA metabolic process
442	GO:0042147	retrograde transport, endosome to Golgi
443	GO:0006271	DNA strand elongation involved in DNA replication
444	GO:0015785	UDP-galactose transport
445	GO:0000165	MAPK cascade
446	GO:0046168	glycerol-3-phosphate catabolic process

Continued on next page

Table 1 – continued from previous page

Number	GO term	GO name
447	GO:0042540	hemoglobin catabolic process
448	GO:0034214	protein hexamerization
449	GO:0000338	protein deneddylation
450	GO:0006405	RNA export from nucleus
451	GO:0007076	mitotic chromosome condensation
452	GO:0006646	phosphatidylethanolamine biosynthetic process
453	GO:0006656	phosphatidylcholine biosynthetic process
454	GO:0030150	protein import into mitochondrial matrix
455	GO:0006435	threonyl-tRNA aminoacylation
456	GO:0006399	tRNA metabolic process
457	GO:0046080	dUTP metabolic process
458	GO:0000070	mitotic sister chromatid segregation
459	GO:0008202	steroid metabolic process
460	GO:0006833	water transport
461	GO:0009247	glycolipid biosynthetic process
462	GO:0015791	polyol transport
463	GO:0051475	glucosylglycerol transport
464	GO:0006546	glycine catabolic process
465	GO:0006813	potassium ion transport
466	GO:0006505	GPI anchor metabolic process
467	GO:0051604	protein maturation
468	GO:0030048	actin filament-based movement
469	GO:0042777	plasma membrane ATP synthesis coupled proton transport
470	GO:0000902	cell morphogenesis
471	GO:0008360	regulation of cell shape
472	GO:0009103	lipopolysaccharide biosynthetic process
473	GO:0009252	peptidoglycan biosynthetic process
474	GO:0006891	intra-Golgi vesicle-mediated transport
475	GO:0010468	regulation of gene expression
476	GO:0018343	protein farnesylation
477	GO:0042776	mitochondrial ATP synthesis coupled proton transport
478	GO:0001510	RNA methylation
479	GO:0009452	7-methylguanosine RNA capping
480	GO:0001819	positive regulation of cytokine production
481	GO:0006452	translational frameshifting
482	GO:0008612	peptidyl-lysine modification to hypusine
483	GO:0045901	positive regulation of translational elongation
484	GO:0045905	positive regulation of translational termination
485	GO:0009117	nucleotide metabolic process
486	GO:0046069	cGMP catabolic process
487	GO:0033014	tetrapyrrole biosynthetic process
488	GO:0006433	prolyl-tRNA aminoacylation
489	GO:0005978	glycogen biosynthetic process
490	GO:0006108	malate metabolic process
491	GO:0006617	SRP-dependent cotranslational protein targeting to membrane, signal sequence recognition
492	GO:0006268	DNA unwinding involved in replication
493	GO:0030071	regulation of mitotic metaphase/anaphase transition
494	GO:0015717	triose phosphate transport
495	GO:0000910	cytokinesis
496	GO:0001932	regulation of protein phosphorylation

Continued on next page

Table 1 – continued from previous page

Number	GO term	GO name
497	GO:0006406	mRNA export from nucleus
498	GO:0006611	protein export from nucleus
499	GO:0006998	nuclear envelope organization
500	GO:0015917	aminophospholipid transport
501	GO:0046654	tetrahydrofolate biosynthetic process
502	GO:0016458	gene silencing
503	GO:0018063	cytochrome c-heme linkage
504	GO:0006275	regulation of DNA replication
505	GO:0051259	protein oligomerization
506	GO:0018055	peptidyl-lysine lipoylation
507	GO:0020012	evasion or tolerance of host immune response
508	GO:0007015	actin filament organization
509	GO:0006544	glycine metabolic process
510	GO:0006563	L-serine metabolic process
511	GO:0006481	C-terminal protein methylation
512	GO:0032513	negative regulation of protein phosphatase type 2B activity
513	GO:0006388	tRNA splicing, via endonucleolytic cleavage and ligation
514	GO:0010038	response to metal ion
515	GO:0006928	cellular component movement
516	GO:0006233	dTDP biosynthetic process
517	GO:0006235	dTTP biosynthetic process
518	GO:0006436	tryptophanyl-tRNA aminoacylation
519	GO:0006032	chitin catabolic process
520	GO:0000278	mitotic cell cycle
521	GO:0006266	DNA ligation
522	GO:0006273	lagging strand elongation
523	GO:0006402	mRNA catabolic process
524	GO:0006538	glutamate catabolic process
525	GO:0001906	cell killing
526	GO:0031120	snRNA pseudouridine synthesis
527	GO:0006278	RNA-dependent DNA replication
528	GO:0009249	protein lipoylation
529	GO:0006661	phosphatidylinositol biosynthetic process
530	GO:0030488	tRNA methylation
531	GO:0051298	centrosome duplication
532	GO:0045017	glycerolipid biosynthetic process
533	GO:0045047	protein targeting to ER
534	GO:0009056	catabolic process
535	GO:0006089	lactate metabolic process
536	GO:0000162	tryptophan biosynthetic process
537	GO:0006342	chromatin silencing
538	GO:0006476	protein deacetylation
539	GO:0016233	telomere capping
540	GO:0006196	AMP catabolic process
541	GO:0006621	protein retention in ER lumen
542	GO:0006425	glutaminyl-tRNA aminoacylation
543	GO:0006304	DNA modification
544	GO:0042256	mature ribosome assembly
545	GO:0007600	sensory perception
546	GO:0017006	protein-tetrapyrrole linkage

Continued on next page

Table 1 – continued from previous page

Number	GO term	GO name
547	GO:0018106	peptidyl-histidine phosphorylation
548	GO:0018298	protein-chromophore linkage
549	GO:0050896	response to stimulus
550	GO:0006447	regulation of translational initiation by iron
551	GO:0000076	DNA replication checkpoint
552	GO:0007090	regulation of S phase of mitotic cell cycle
553	GO:0006047	UDP-N-acetylglucosamine metabolic process
554	GO:0016925	protein sumoylation
555	GO:0006102	isocitrate metabolic process
556	GO:0006863	purine nucleobase transport
557	GO:0032238	adenosine transport
558	GO:0006430	lysyl-tRNA aminoacylation
559	GO:0006071	glycerol metabolic process
560	GO:0006167	AMP biosynthetic process
561	GO:0006915	apoptotic process
562	GO:0006561	proline biosynthetic process
563	GO:0008154	actin polymerization or depolymerization
564	GO:0006659	phosphatidylserine biosynthetic process
565	GO:0006419	alanyl-tRNA aminoacylation
566	GO:0006797	polyphosphate metabolic process
567	GO:0031119	tRNA pseudouridine synthesis
568	GO:0006171	cAMP biosynthetic process
569	GO:0006314	intron homing
570	GO:0016574	histone ubiquitination
571	GO:0006206	pyrimidine nucleobase metabolic process
572	GO:0045727	positive regulation of translation
573	GO:0006479	protein methylation
574	GO:0050983	deoxyhypusine biosynthetic process from spermidine
575	GO:0006499	N-terminal protein myristoylation
576	GO:0016049	cell growth
577	GO:0006426	glycyl-tRNA aminoacylation
578	GO:0035434	copper ion transmembrane transport
579	GO:0006635	fatty acid beta-oxidation
580	GO:0009062	fatty acid catabolic process
581	GO:0016571	histone methylation
582	GO:0015977	carbon fixation
583	GO:0007050	cell cycle arrest
584	GO:0030833	regulation of actin filament polymerization
585	GO:0030091	protein repair
586	GO:0006537	glutamate biosynthetic process
587	GO:0006576	cellular biogenic amine metabolic process
588	GO:0042262	DNA protection
589	GO:0006944	cellular membrane fusion
590	GO:0032012	regulation of ARF protein signal transduction
591	GO:0010608	posttranscriptional regulation of gene expression
592	GO:0042594	response to starvation
593	GO:0045595	regulation of cell differentiation
594	GO:0042167	heme catabolic process
595	GO:0006384	transcription initiation from RNA polymerase III promoter
596	GO:0048193	Golgi vesicle transport
		Continued on next page

Table 1 – continued from previous page

Number	GO term	GO name
597	GO:0006006	glucose metabolic process
598	GO:0051156	glucose 6-phosphate metabolic process
599	GO:0006879	cellular iron ion homeostasis
600	GO:0006885	regulation of pH
601	GO:0006662	glycerol ether metabolic process
602	GO:0002474	antigen processing and presentation of peptide antigen via MHC class I
603	GO:0006955	immune response
604	GO:0031952	regulation of protein autophosphorylation
605	GO:0045737	positive regulation of cyclin-dependent protein kinase activity
606	GO:0042255	ribosome assembly
607	GO:0009187	cyclic nucleotide metabolic process
608	GO:0008272	sulfate transport
609	GO:0019932	second-messenger-mediated signaling
610	GO:0033205	cell cycle cytokinesis
611	GO:0006325	chromatin organization
612	GO:0019856	pyrimidine nucleobase biosynthetic process
613	GO:0030497	fatty acid elongation
614	GO:0009060	aerobic respiration
