# Supplementary material for: Integrative omics analysis. A study based on Plasmodium falciparum mRNA and protein data
Source: BMC Syst Biol. 2014 Mar 13;8(Suppl 2):S4. doi: 10.1186/1752-0509-8-S2-S4 (PMC4101701; doi:10.1186/1752-0509-8-S2-S4)
Supplement: Additional file 3 — CIA general GO term associations in gene space. PDF file containing the CIA general GO term associations in protein space. [file 1752-0509-8-S2-S4-S3.pdf]

PDF file containing the CIA general GO term associations in protein space.

Table 1: General CIA associations in protein space.

**Stage**	**GO term**
Gametocyte	1: GO:0009405, 21: GO:0055114, 32: GO:0006816, 39: GO:0007165, 51: GO:0007021, 52: GO:0006474, 58: GO:0006366, 64: GO:0007155, 72: GO:0016117, 78: GO:0006353, 84: GO:0009073, 92: GO:0009790, 93: GO:0030216, 96: GO:0006396, 104: GO:0032889, 109: GO:0006415, 110: GO:0006449, 120: GO:0006267, 122: GO:0006904, 130: GO:0000375, 131: GO:0008380, 136: GO:0018345, 139: GO:0045454, 145: GO:0006072, 146: GO:0006127, 148: GO:0016567, 151: GO:0046823, 152: GO:0060284, 173: GO:0006488, 176: GO:0019432, 183: GO:0006554, 186: GO:0006744, 202: GO:0016480, 203: GO:0017148, 208: GO:0043248, 225: GO:0006437, 226: GO:0000122, 241: GO:0015904, 242: GO:0046677, 244: GO:0051016, 248: GO:0043487, 250: GO:0048034, 256: GO:0006777, 262: GO:0018344, 263: GO:0006207, 264: GO:0006222, 265: GO:0051302, 270: GO:0019368, 273: GO:0006743, 276: GO:0044262, 278: GO:0006357, 290: GO:0042128, 300: GO:0006122, 304: GO:0006401, 309: GO:0001682, 310: GO:0006729, 317: GO:0000105, 319: GO:0009086, 320: GO:0009396, 321: GO:0010501, 325: GO:0007154, 331: GO:0030261, 339: GO:0042779, 343: GO:0007530, 346: GO:0045900, 347: GO:0000256, 348: GO:0006144, 351: GO:0006536, 356: GO:0006761, 357: GO:0042558, 366: GO:0006103, 374: GO:0051205, 375: GO:0042127, 379: GO:0043686, 382: GO:0007219, 386: GO:0046836, 387: GO:0018279, 402: GO:0000184, 408: GO:0006788, 420: GO:0009298, 421: GO:0019307, 430: GO:0045893, 438: GO:0016539, 443: GO:0006271, 445: GO:0000165, 476: GO:0018343, 477: GO:0042776, 481: GO:0006452, 482: GO:0008612, 483: GO:0045901, 484: GO:0045905, 486: GO:0046069, 490: GO:0006108, 493: GO:0030071, 497: GO:0006406, 498: GO:0006611, 499: GO:0006998, 503: GO:0018063, 511: GO:0006481, 513: GO:0006388, 514: GO:0010038, 516: GO:0006233, 517: GO:0006235, 519: GO:0006032, 523: GO:0006402, 526: GO:0031120, 530: GO:0030488, 531: GO:0051298, 535: GO:0006089, 536: GO:0000162, 539: GO:0016233, 550: GO:0006447, 551: GO:0000076, 552: GO:0007090, 555: GO:0006102, 561: GO:0006915, 564: GO:0006659, 567: GO:0031119, 568: GO:0006171, 570: GO:0016574, 571: GO:0006206, 572: GO:0045727, 579: GO:0006635, 580: GO:0009062, 582: GO:0015977, 585: GO:0030091, 586: GO:0006537, 598: GO:0051156, 599: GO:0006879, 604: GO:0031952, 605: GO:0045737, 608: GO:0008272, 610: GO:0033205, 612: GO:0019856
Sporozoite	2: GO:0016337, 3: GO:0020013, 4: GO:0020033, 5: GO:0020035, 10: GO:0006468, 14: GO:0006887, 17: GO:0006817, 18: GO:0006810, 20: GO:0019288, 22: GO:0055085, 23: GO:0007010, 27: GO:0006139, 28: GO:0015986, 29: GO:0015991, 30: GO:0006754, 31: GO:0006812, 36: GO:0006184, 41: GO:0060627, 42: GO:0008299, 43: GO:0007067, 45: GO:0006281, 46: GO:0006302, 48: GO:0006260, 50: GO:0006461, 53: GO:0006355, 54: GO:0035556, 55: GO:0006432, 60: GO:0048015, 62: GO:0006367, 63: GO:0007018, 65: GO:0006221, 66: GO:0006428, 69: GO:0019835, 70: GO:0019836, 73: GO:0006289, 74: GO:0007017, 79: GO:0006397, 80: GO:0008646, 81: GO:0045426, 86: GO:0006163, 87: GO:0006188, 88: GO:0009152, 90: GO:0016226, 94: GO:0006633, 97: GO:0016114, 98: GO:0006836, 101: GO:0015718, 102: GO:0016192, 103: GO:0031338, 107: GO:0001522, 108: GO:0009451, 111: GO:0006855, 115: GO:0006888, 119: GO:0015909, 123: GO:0006869, 124: GO:0006897, 125: GO:0008203, 126: GO:0015992, 127: GO:0046034, 128: GO:0006974, 129: GO:0000027, 133: GO:0000398, 134: GO:0000154, 137: GO:0000226, 153: GO:0006529, 154: GO:0006438, 156: GO:0006487, 160: GO:0032780, 161: GO:0051131, 162: GO:0002312, 163: GO:0006796, 164: GO:0051301, 165: GO:0006378, 166: GO:0006379, 168: GO:0015914, 169: GO:0009966, 171: GO:0006465, 174: GO:0007275, 175: GO:0006370, 177: GO:0032313, 178: GO:0015908, 179: GO:0016579, 180: GO:0000245, 185: GO:0009438, 187: GO:0009234, 188: GO:0006354, 189: GO:0008295, 190: GO:0006261, 191: GO:0051052, 192: GO:0006400, 194: GO:0015684, 196: GO:0006811, 197: GO:0006875, 198: GO:0046685, 200: GO:0006270, 201: GO:0016568, 204: GO:0006231, 205: GO:0006545, 207: GO:0009165, 209: GO:0006182, 210: GO:0018144, 212: GO:0051258, 214: GO:0006429, 216: GO:0045039, 217: GO:0016310, 218: GO:0030036, 219: GO:0060327, 220: GO:0006099, 221: GO:0008643, 227: GO:0030154, 228: GO:0046323, 230: GO:0006360, 232: GO:0046854, 236: GO:0006750, 237: GO:0009116, 238: GO:0000917, 239: GO:0009052, 240: GO:0008033, 245: GO:0000079, 246: GO:0045736, 249: GO:0006818, 251: GO:0009228, 254: GO:0007186, 257: GO:0032312, 258: GO:0043087, 259: GO:0009245, 260: GO:0007169, 266: GO:0006165, 267: GO:0006183, 268: GO:0006228, 269: GO:0006241, 271: GO:0006779, 272: GO:0006783, 275: GO:0030001, 279: GO:0034227, 280: GO:0009306, 281: GO:0006284, 286: GO:0048870, 287: GO:0006090, 288: GO:0006820, 292: GO:0009432, 293: GO:0006097, 298: GO:0007034, 299: GO:0042144, 301: GO:0045836, 302: GO:0006801, 303: GO:0019430, 305: GO:0031123, 306: GO:0043631, 312: GO:0009435, 313: GO:0019357, 314: GO:0019358, 315: GO:0006865, 316: GO:0000082, 318: GO:0006164, 322: GO:0032508, 323: GO:0006814, 326: GO:0015937, 327: GO:0045173, 328: GO:0006825, 329: GO:0006878, 330: GO:0008535, 333: GO:0006534, 334: GO:0008616, 335: GO:0006434, 336: GO:0030522, 338: GO:0000280, 340: GO:0051028, 341: GO:0009607, 342: GO:0042113, 349: GO:0000055, 350: GO:0042273, 352: GO:0009190, 353: GO:0000724, 354: GO:0030259, 355: GO:0006890, 358: GO:0006004, 359: GO:0019673, 360: GO:0006086, 362: GO:0007131, 365: GO:0006655, 367: GO:0009107, 368: GO:0006835, 370: GO:0015742, 371: GO:0015743, 373: GO:0007059, 377: GO:0006525, 378: GO:0031365, 380: GO:0006308, 381: GO:0031167, 383: GO:0018342, 384: GO:0006269, 385: GO:0006420, 389: GO:0006541, 390: GO:0008153, 392: GO:0006807, 393: GO:0006772, 394: GO:0009229, 395: GO:0016575, 396: GO:0007205, 397: GO:0007020, 398: GO:0015074, 399: GO:0032196, 400: GO:0006427, 403: GO:0007266, 404: GO:0017183, 405: GO:0006493, 406: GO:0006665, 412: GO:0009225, 413: GO:0006423, 419: GO:0007030, 423: GO:0009264, 424: GO:0042773, 425: GO:0006101, 426: GO:0006041, 428: GO:0019353, 431: GO:0006596, 432: GO:0006597, 433: GO:0009445, 434: GO:0022900, 437: GO:0022904, 439: GO:0019478, 441: GO:0006104, 442: GO:0042147, 449: GO:0000338, 451: GO:0007076, 457: GO:0046080, 458: GO:0000070, 459: GO:0008202, 467: GO:0051604, 468: GO:0030048, 470: GO:0000902, 471: GO:0008360, 472: GO:0009103, 473: GO:0009252, 478: GO:0001510, 479: GO:0009452, 485: GO:0009117, 487: GO:0033014, 488: GO:0006433, 489: GO:0005978, 491: GO:0006617, 492: GO:0006268, 494: GO:0015717, 495: GO:0000910, 500: GO:0015917, 501: GO:0046654, 502: GO:0016458, 504: GO:0006275, 508: GO:0007015, 515: GO:0006928, 518: GO:0006436, 520: GO:0000278, 521: GO:0006266, 522: GO:0006273, 524: GO:0006538, 525: GO:0001906, 527: GO:0006278, 534: GO:0009056, 537: GO:0006342, 538: GO:0006476, 540: GO:0006196, 541: GO:0006621, 543: GO:0006304, 545: GO:0007600, 546: GO:0017006, 547: GO:0018106, 548: GO:0018298, 553: GO:0006047, 563: GO:0008154, 565: GO:0006419, 566: GO:0006797, 573: GO:0006479, 574: GO:0050983, 575: GO:0006499, 577: GO:0006426, 578: GO:0035434, 584: GO:0030833, 590: GO:0032012, 593: GO:0045595, 594: GO:0042167, 595: GO:0006384, 596: GO:0048193, 606: GO:0042255, 607: GO:0009187, 609: GO:0019932, 613: GO:0030497, 614: GO:0009060
Merozoite	9: GO:0030260, 12: GO:0006418, 24: GO:0006364, 56: GO:0043039, 59: GO:0046488, 71: GO:0006464, 77: GO:0006351, 83: GO:0042176, 89: GO:0016255, 95: GO:0006629, 105: GO:0008610, 117: GO:0044409, 118: GO:0006631, 135: GO:0000059, 140: GO:0043687, 144: GO:0006979, 149: GO:0006470, 150: GO:0016311, 155: GO:0006486, 157: GO:0018105, 182: GO:0016070, 222: GO:0006298, 224: GO:0042787, 231: GO:0006310, 235: GO:0008654, 253: GO:0042493, 261: GO:0016043, 282: GO:0006506, 291: GO:0005975, 294: GO:0006352, 297: GO:0043412, 307: GO:0051289, 308: GO:0006091, 324: GO:0006644, 337: GO:0006458, 345: GO:0006614, 376: GO:0051262, 391: GO:0006542, 410: GO:0006177, 411: GO:0006914, 416: GO:0000045, 417: GO:0002253, 418: GO:0006094, 422: GO:0006471, 427: GO:0016051, 429: GO:0009168, 435: GO:0006898, 436: GO:0007276, 453: GO:0006656, 455: GO:0006435, 456: GO:0006399, 465: GO:0006813, 466: GO:0006505, 469: GO:0042777, 475: GO:0010468, 506: GO:0018055, 507: GO:0020012, 509: GO:0006544, 528: GO:0009249, 529: GO:0006661, 532: GO:0045017, 533: GO:0045047, 542: GO:0006425, 554: GO:0016925, 559: GO:0006071, 560: GO:0006167, 581: GO:0016571, 587: GO:0006576, 589: GO:0006944, 592: GO:0042594, 601: GO:0006662
Trophozoite	7: GO:0006457, 11: GO:0006412, 25: GO:0032259, 34: GO:0006413, 35: GO:0042254, 40: GO:0007264, 44: GO:0006414, 49: GO:0009408, 57: GO:0008283, 67: GO:0006417, 113: GO:0006986, 114: GO:0044267, 121: GO:0006338, 141: GO:0051246, 143: GO:0065002, 170: GO:0043666, 184: GO:0007035, 199: GO:0046939, 211: GO:0009059, 213: GO:0006446, 215: GO:0006626, 277: GO:0006098, 283: GO:0000304, 284: GO:0008614, 285: GO:0042819, 289: GO:0044070, 295: GO:0008615, 296: GO:0042823, 344: GO:0006306, 361: GO:0019538, 369: GO:0006839, 414: GO:0042026, 440: GO:0046777, 444: GO:0015785, 446: GO:0046168, 447: GO:0042540, 450: GO:0006405, 454: GO:0030150, 464: GO:0006546, 496: GO:0001932, 512: GO:0032513, 544: GO:0042256, 549: GO:0050896, 558: GO:0006430, 562: GO:0006561, 583: GO:0007050, 591: GO:0010608

Continued on next page

Table 1 – continued from previous page

Stage	GO term
Ring	6: GO:0042000, 8: GO:0050776, 13: GO:0006422, 15: GO:0015031, 16: GO:0006508, 19: GO:0006511, 26: GO:0007049, 33: GO:0008152, 37: GO:0006886, 38: GO:0006913, 47: GO:0051603, 61: GO:0006334, 68: GO:0006259, 75: GO:0006520, 76: GO:0009058, 82: GO:0044237, 85: GO:0006383, 91: GO:0002377, 99: GO:0006605, 100: GO:0043952, 106: GO:0006421, 112: GO:0030163, 116: GO:0044053, 132: GO:0016042, 138: GO:0006467, 142: GO:0017038, 147: GO:0006950, 158: GO:0010564, 159: GO:0030433, 167: GO:0006096, 172: GO:0006333, 181: GO:0046907, 193: GO:0051276, 195: GO:0006606, 206: GO:0006730, 223: GO:0019941, 229: GO:0006323, 233: GO:0006265, 234: GO:0006021, 243: GO:0009987, 247: GO:0051726, 252: GO:0008104, 255: GO:0006607, 274: GO:0006591, 311: GO:0006424, 332: GO:0006829, 363: GO:0002720, 364: GO:0006359, 372: GO:0015858, 388: GO:0006556, 401: GO:0006431, 407: GO:0043161, 409: GO:0006166, 415: GO:0009186, 448: GO:0034214, 452: GO:0006646, 460: GO:0006833, 461: GO:0009247, 462: GO:0015791, 463: GO:0051475, 474: GO:0006891, 480: GO:0001819, 505: GO:0051259, 510: GO:0006563, 556: GO:0006863, 557: GO:0032238, 569: GO:0006314, 576: GO:0016049, 588: GO:0042262, 597: GO:0006006, 600: GO:0006885, 602: GO:0002474, 603: GO:0006955, 611: GO:0006325
Schizont	6: GO:0042000, 8: GO:0050776, 13: GO:0006422, 15: GO:0015031, 16: GO:0006508, 19: GO:0006511, 26: GO:0007049, 33: GO:0008152, 37: GO:0006886, 38: GO:0006913, 47: GO:0051603, 61: GO:0006334, 68: GO:0006259, 75: GO:0006520, 76: GO:0009058, 82: GO:0044237, 85: GO:0006383, 91: GO:0002377, 99: GO:0006605, 100: GO:0043952, 106: GO:0006421, 112: GO:0030163, 116: GO:0044053, 132: GO:0016042, 138: GO:0006467, 142: GO:0017038, 147: GO:0006950, 158: GO:0010564, 159: GO:0030433, 167: GO:0006096, 172: GO:0006333, 181: GO:0046907, 193: GO:0051276, 195: GO:0006606, 206: GO:0006730, 223: GO:0019941, 229: GO:0006323, 233: GO:0006265, 234: GO:0006021, 243: GO:0009987, 247: GO:0051726, 252: GO:0008104, 255: GO:0006607, 274: GO:0006591, 311: GO:0006424, 332: GO:0006829, 363: GO:0002720, 364: GO:0006359, 372: GO:0015858, 388: GO:0006556, 401: GO:0006431, 407: GO:0043161, 409: GO:0006166, 415: GO:0009186, 448: GO:0034214, 452: GO:0006646, 460: GO:0006833, 461: GO:0009247, 462: GO:0015791, 463: GO:0051475, 474: GO:0006891, 480: GO:0001819, 505: GO:0051259, 510: GO:0006563, 556: GO:0006863, 557: GO:0032238, 569: GO:0006314, 576: GO:0016049, 588: GO:0042262, 597: GO:0006006, 600: GO:0006885, 602: GO:0002474, 603: GO:0006955, 611: GO:0006325
