# Supplementary material for: Integrative omics analysis. A study based on Plasmodium falciparum mRNA and protein data
Source: BMC Syst Biol. 2014 Mar 13;8(Suppl 2):S4. doi: 10.1186/1752-0509-8-S2-S4 (PMC4101701; doi:10.1186/1752-0509-8-S2-S4)
Supplement: Additional file 4 — CIA division limits. PDF file containing the CIA division limits for general (left) and specific (right) associations. The colours of the areas correspond to the colours of the stages they are associated with. [file 1752-0509-8-S2-S4-S4.pdf]

PDF file containing the CIA division limits for the general (left) and specific (right) associations in gene (Figure 1) and protein space (Figure 2). The colors of the areas correspond to the colors of the stages they are associated with.

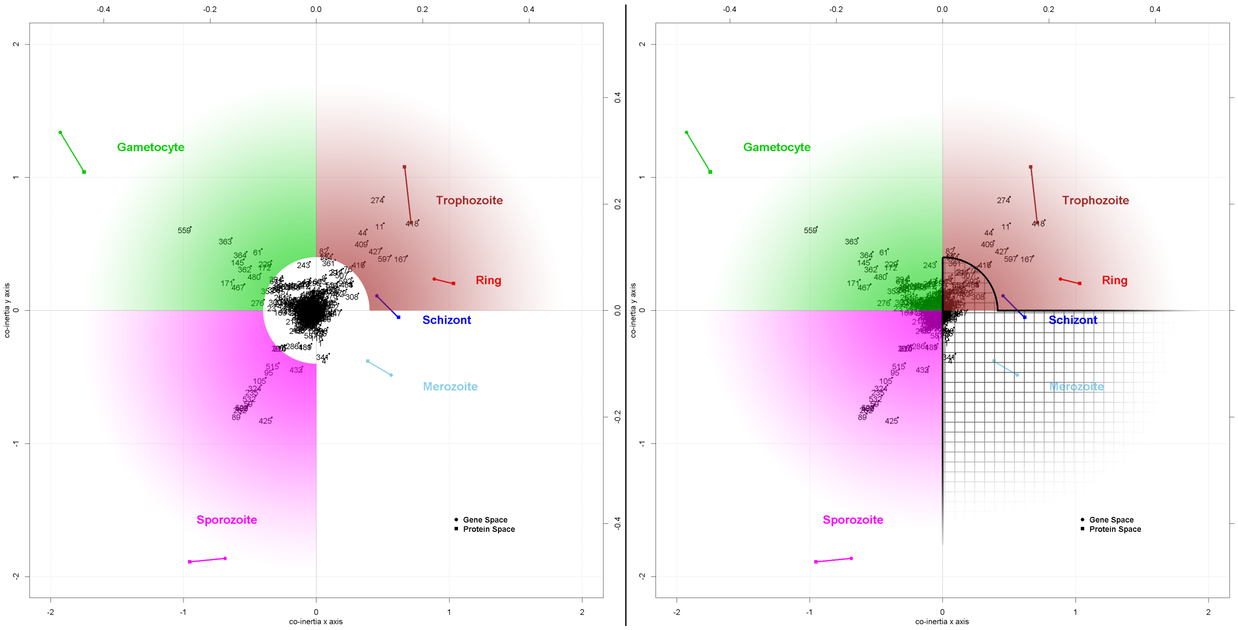

Figure 1: CIA division limits in gene space.

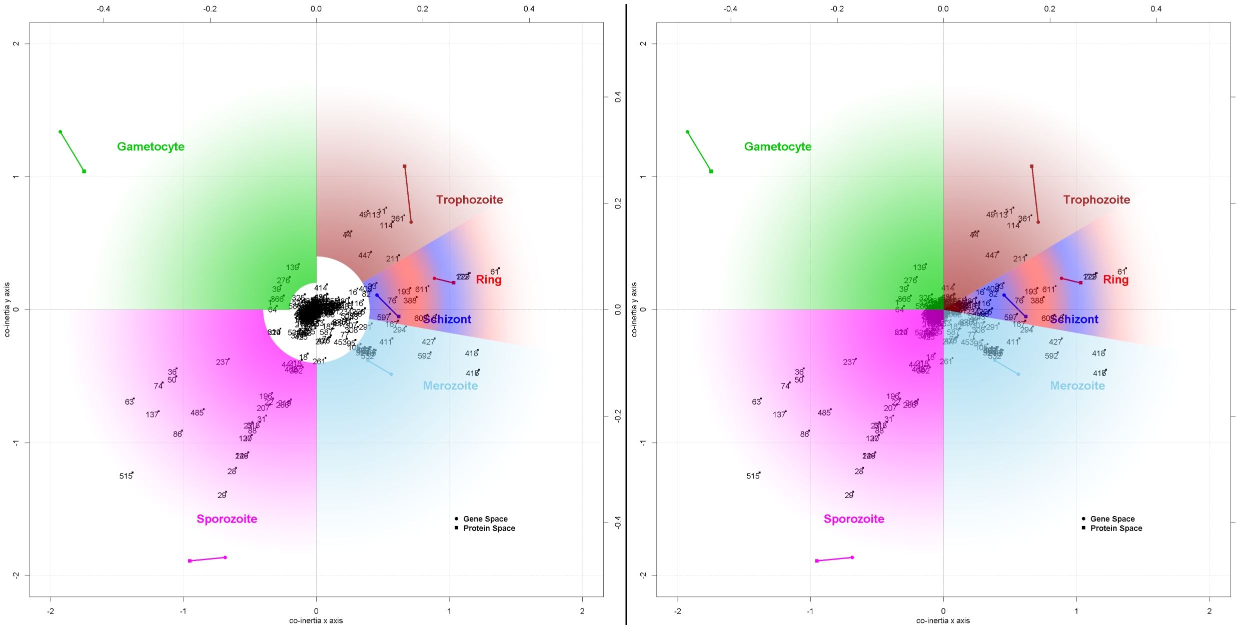

Figure 2: CIA division limits in protein space.
