# Supplementary material for: Integrative omics analysis. A study based on Plasmodium falciparum mRNA and protein data
Source: BMC Syst Biol. 2014 Mar 13;8(Suppl 2):S4. doi: 10.1186/1752-0509-8-S2-S4 (PMC4101701; doi:10.1186/1752-0509-8-S2-S4)
Supplement: Additional file 5 — CIA specific GO term associations in gene space. PDF file containing the CIA specific GO term associations in gene space. [file 1752-0509-8-S2-S4-S5.pdf]

PDF file containing the CIA specific GO term associations in gene space.

Table 1 - CIA specific GO terms association in gene space to the sporozoite stage.

In this table GO term association in gene space to the cell cycle stage sporozoite are presented. The numbers in the left column correspond to the numbers in the left graphic of Figure 1.

CIA: Sporozoite in gene space	
59	GO:0046488: phosphatidylinositol metabolic process
89	GO:0016255: attachment of GPI anchor to protein
95	GO:0006629: lipid metabolic process
105	GO:0008610: lipid biosynthetic process
235	GO:0008654: phospholipid biosynthetic process
282	GO:0006506: GPI anchor biosynthetic process
324	GO:0006644: phospholipid metabolic process
425	GO:0006101: citrate metabolic process
432	GO:0006597: spermine biosynthetic process
433	GO:0009445: putrescine metabolic process
466	GO:0006505: GPI anchor metabolic process
529	GO:0006661: phosphatidylinositol biosynthetic process
532	GO:0045017: glycerolipid biosynthetic process

Table 2 - CIA specific GO terms association in gene space to the trophzoite stage.

In this table GO term association in gene space to the cell cycle stage trophozoite are presented. The numbers in the left column correspond to the numbers in the left graphic of Figure 1.

CIA: Trophozoite in gene space	
11	GO:0006412: translation
33	GO:0008152: metabolic process
44	GO:0006414: translational elongation
82	GO:0044237: cellular metabolic process
114	GO:0044267: cellular protein metabolic process
167	GO:0006096: glycolysis
274	GO:0006591: ornithine metabolic process
409	GO:0006166: purine ribonucleoside salvage
418	GO:0006094: gluconeogenesis
427	GO:0016051: carbohydrate biosynthetic process
597	GO:0006006: glucose metabolic process
