# Supplementary material for: Integrative omics analysis. A study based on Plasmodium falciparum mRNA and protein data
Source: BMC Syst Biol. 2014 Mar 13;8(Suppl 2):S4. doi: 10.1186/1752-0509-8-S2-S4 (PMC4101701; doi:10.1186/1752-0509-8-S2-S4)
Supplement: Additional file 6 — CIA specific GO term associations in protein space. PDF file containing the CIA specific GO term associations in protein space. [file 1752-0509-8-S2-S4-S6.pdf]

PDF file containing the CIA specific GO term associations in protein space.

Table 1 - CIA specific GO terms association in protein space to the sporozoite stage.

In this table GO term association in protein space to the cell cycle stage sporozoite are presented. The numbers in the left column correspond to the numbers in the right graphic of Figure 1.

CIA: Sporozoite in protein space	
22	GO:0055085: transmembrane transport
23	GO:0007010: cytoskeleton organization
27	GO:0006139: nucleobase-containing compound metabolic process
28	GO:0015986: ATP synthesis coupled proton transport
29	GO:0015991: ATP hydrolysis coupled proton transport
30	GO:0006754: ATP biosynthetic process
31	GO:0006812: cation transport
36	GO:0006184: GTP catabolic process
50	GO:0006461: protein complex assembly
63	GO:0007018: microtubule-based movement
74	GO:0007017: microtubule-based process
86	GO:0006163: purine nucleotide metabolic process
88	GO:0009152: purine ribonucleotide biosynthetic process
126	GO:0015992: proton transport
127	GO:0046034: ATP metabolic process
137	GO:0000226: microtubule cytoskeleton organization
196	GO:0006811: ion transport
207	GO:0009165: nucleotide biosynthetic process
212	GO:0051258: protein polymerization
219	GO:0060327: cytoplasmic actin-based contraction involved in cell motility
237	GO:0009116: nucleoside metabolic process
249	GO:0006818: hydrogen transport
286	GO:0048870: cell motility
318	GO:0006164: purine nucleotide biosynthetic process
392	GO:0006807: nitrogen compound metabolic process
441	GO:0006104: succinyl-CoA metabolic process
468	GO:0030048: actin filament-based movement
485	GO:0009117: nucleotide metabolic process
515	GO:0006928: cellular component movement

Table 2 - CIA specific GO terms association in protein space to merozoite stage.

In this table GO term association in protein space to the merozoite stage are presented. The numbers in the left column correspond to the numbers in the right graphic of Figure 1.

CIA: Merozoite in protein space	
59	GO:0046488: phosphatidylinositol metabolic process
89	GO:0016255: attachment of GPI anchor to protein
105	GO:0008610: lipid biosynthetic process
235	GO:0008654: phospholipid biosynthetic process
282	GO:0006506: GPI anchor biosynthetic process
291	GO:0005975: carbohydrate metabolic process
294	GO:0006352: DNA-dependent transcription, initiation
324	GO:0006644: phospholipid metabolic process
411	GO:0006914: autophagy
416	GO:0000045: autophagic vacuole assembly
417	GO:0002253: activation of immune response
418	GO:0006094: gluconeogenesis
427	GO:0016051: carbohydrate biosynthetic process
466	GO:0006505: GPI anchor metabolic process
529	GO:0006661: phosphatidylinositol biosynthetic process
532	GO:0045017: glycerolipid biosynthetic process
592	GO:0042594: response to starvation

Table 3 - CIA specific GO terms association in protein space to the trophzoite stage.

In this table GO term association in protein space to the cell cycle stage trophozoite are presented. The numbers in the left column correspond to the numbers in the right graphic of Figure 1.

CIA: Trophozoite in protein space	
7	GO:0006457: protein folding
11	GO:0006412: translation
44	GO:0006414: translational elongation
49	GO:0009408: response to heat
113	GO:0006986: response to unfolded protein
114	GO:0044267: cellular protein metabolic process
211	GO:0009059: macromolecule biosynthetic process
361	GO:0019538: protein metabolic process
447	GO:0042540: hemoglobin catabolic process

Table 4 - CIA specific GO terms association in protein space to ring and schizont stages.

In this table GO term association in protein space to the cell cycle stages ring and schizont are presented. The numbers in the left column correspond to the numbers in the right graphic of Figure 1.

CIA: Ring and schizont in protein space	
8	GO:0050776: regulation of immune response
33	GO:0008152: metabolic process
61	GO:0006334: nucleosome assembly
76	GO:0009058: biosynthetic process
82	GO:0044237: cellular metabolic process
167	GO:0006096: glycolysis
172	GO:0006333: chromatin assembly or disassembly
193	GO:0051276: chromosome organization
229	GO:0006323: DNA packaging
388	GO:0006556: S-adenosylmethionine biosynthetic process
409	GO:0006166: purine ribonucleoside salvage
597	GO:0006006: glucose metabolic process
603	GO:0006955: immune response
611	GO:0006325: chromatin organization
