# Supplementary material for: Integrative omics analysis. A study based on Plasmodium falciparum mRNA and protein data
Source: BMC Syst Biol. 2014 Mar 13;8(Suppl 2):S4. doi: 10.1186/1752-0509-8-S2-S4 (PMC4101701; doi:10.1186/1752-0509-8-S2-S4)
Supplement: Additional file 7 — GSVD general GO term associations. PDF file containing the GSVD based general associations of GO terms to life cycle stages in common space. [file 1752-0509-8-S2-S4-S7.pdf]

PDF file containing the GSVD based general associations of GO terms to cell cycle stages.

Table 1: GSVD based general GO terms associations to cell cycle stages.

Stage	GO terms
Gametocyte	GO:0044238, GO:0008152, GO:0044237, GO:0045017, GO:0043170, GO:0034645, GO:0046474, GO:0009059, GO:0022613, GO:0044260, GO:0019538, GO:0046486, GO:0042254, GO:0006839, GO:0009987, GO:0071843, GO:0009058, GO:0006497, GO:0042157, GO:0042158, GO:0006650, GO:0016255, GO:0006661, GO:0044249, GO:0006506, GO:0044267, GO:0008654, GO:0006644, GO:0010467, GO:0002682, GO:0006626, GO:0007005, GO:0050776, GO:0070585, GO:0072655, GO:0006323, GO:0046488, GO:0006505, GO:0071841, GO:0019637, GO:0006006, GO:0071840, GO:0002376, GO:0006812, GO:0000041, GO:0002440, GO:0042592, GO:0006007, GO:0019320, GO:0046365, GO:0051246, GO:0006996, GO:0006412, GO:0006955
Sporozoite	GO:0009987, GO:0008152, GO:0019538, GO:0044237, GO:0009056, GO:0019219, GO:0051171, GO:0065007, GO:0044238, GO:0034645, GO:0006457, GO:0009116, GO:0032774, GO:0009059, GO:0006091, GO:0044267, GO:0050789, GO:0055085, GO:0072521, GO:0044270, GO:0006006, GO:0006351, GO:0044249, GO:0055114, GO:0050794, GO:0080090, GO:0046700, GO:0009119, GO:0042278, GO:0046128, GO:0006007, GO:0019320, GO:0046365, GO:0009058, GO:0006355, GO:0051252, GO:2001141, GO:0033365, GO:0006163, GO:0009166, GO:0034655, GO:1901292, GO:0055086, GO:0019222, GO:0006753, GO:0019637, GO:0006098, GO:0006739, GO:0006740, GO:0006195, GO:0072523, GO:0044248, GO:0051701, GO:0031323, GO:0006732, GO:0042221, GO:0009117, GO:0046434, GO:0046039, GO:0009150, GO:0043170, GO:0046907, GO:0051641, GO:0072594, GO:0009144, GO:0009199, GO:0009205, GO:0034613, GO:0070727, GO:0020035, GO:0044406, GO:0051825, GO:0020013, GO:0022407, GO:0030155, GO:0034109, GO:0034110, GO:0034117, GO:0034118, GO:0044068, GO:0006486, GO:0009101, GO:0043413, GO:0006733, GO:0006839, GO:0016052, GO:0009154, GO:0009261
Trophozoite	GO:0044403, GO:0044419, GO:0051704, GO:0009607, GO:0006952, GO:0051707, GO:0051805, GO:0051807, GO:0051832, GO:0051834, GO:0052173, GO:0052564, GO:0020033, GO:0051809, GO:0006091, GO:0050896, GO:0006950, GO:0006096, GO:0015980, GO:0040011, GO:0016485, GO:0045333, GO:0016311, GO:0006006, GO:0016052, GO:0006465, GO:0015931, GO:0030260, GO:0051806, GO:0051701
Ring	GO:0008152, GO:0044238, GO:0044237, GO:0009987, GO:0006091, GO:0006412, GO:0006732, GO:0009058, GO:0055114, GO:0019538, GO:0044249, GO:0051186, GO:0043170, GO:0010467, GO:0045333, GO:0044267, GO:0006955, GO:0006006, GO:0016052, GO:0044260, GO:0006007, GO:0019320, GO:0046365, GO:0005996, GO:0019318, GO:0005975, GO:0051187, GO:0002682, GO:0006334, GO:0031497, GO:0034728, GO:0050776, GO:0071806, GO:0034645, GO:0015980, GO:0009059, GO:0009056, GO:0006325, GO:0006084, GO:0006096, GO:0006099, GO:0009060, GO:0009109, GO:0046356, GO:0002376, GO:0002440, GO:0045454
Schizont	GO:0009607, GO:0006952, GO:0051707, GO:0051805, GO:0051807, GO:0051832, GO:0051834, GO:0052173, GO:0052564, GO:0020033, GO:0051809, GO:0051704, GO:0044403, GO:0044419, GO:0055085, GO:0006325, GO:0080090, GO:0050896, GO:0006950, GO:0019219, GO:0051171, GO:0006355, GO:0051252, GO:2001141, GO:0002682, GO:0050776, GO:0019222, GO:0009166, GO:0034655, GO:1901292, GO:0006084, GO:0006099, GO:0009060, GO:0009109, GO:0046356, GO:0006732, GO:0006195, GO:0072523, GO:0002376, GO:0006366, GO:0009894, GO:0009119, GO:0042278, GO:0046128, GO:0002440, GO:0046434, GO:0009116, GO:0048583, GO:0046039, GO:0065007, GO:0006412, GO:0006955, GO:0009154, GO:0009261, GO:0009117, GO:0031323, GO:0060255, GO:0006753, GO:0072521, GO:0002377, GO:0009889, GO:0010556, GO:0031326, GO:2000112, GO:0045333, GO:0051276, GO:0006163, GO:0006091, GO:1901068

Continued on next page

Table 1 – continued from previous page

Stage	GO terms
Merozoite	GO:0044238, GO:0009987, GO:0008152, GO:0016311, GO:0055086, GO:0019637, GO:0006753, GO:0044237, GO:0019538, GO:0055085, GO:0006006, GO:0006470, GO:0009117, GO:0006007, GO:0019320, GO:0046365, GO:0005996, GO:0019318, GO:0040011, GO:0030260, GO:0051806, GO:0046486, GO:0006464, GO:0036211, GO:0044267, GO:0016052, GO:0045017, GO:0006913, GO:0051169, GO:0006644, GO:0019362, GO:0046496, GO:0072524, GO:0044409, GO:0051828, GO:0052126, GO:0052192, GO:0006650, GO:0019219, GO:0051171, GO:0019222, GO:0046474, GO:0006497, GO:0042157, GO:0042158, GO:0009056, GO:0006810, GO:0051234, GO:0051179, GO:0009058, GO:0006355, GO:0051252, GO:2001141, GO:0006505, GO:0044249, GO:0016255, GO:0010468, GO:0009259, GO:0008654, GO:0009116, GO:0034645, GO:0043170, GO:0060255, GO:0006098, GO:0006576, GO:0006739, GO:0006740, GO:0009308, GO:0044106, GO:0006506, GO:0009150, GO:0009059, GO:0006096, GO:0044255, GO:0044281, GO:0006508, GO:1901135, GO:0031323, GO:0006357, GO:0015931, GO:0080090, GO:0071704, GO:0009119, GO:0042278, GO:0046128, GO:0044270, GO:0046488, GO:0046483, GO:0072521, GO:0005975, GO:0006661, GO:0007264, GO:0006629, GO:0006986, GO:0010033, GO:0035966, GO:0009889, GO:0010556, GO:0031326, GO:2000112, GO:0046700, GO:0009144, GO:0009199, GO:0009205, GO:0007059, GO:0009123, GO:0009124, GO:0043412, GO:0042221

—l—l—
