# Supplementary material for: Integrative omics analysis. A study based on Plasmodium falciparum mRNA and protein data
Source: BMC Syst Biol. 2014 Mar 13;8(Suppl 2):S4. doi: 10.1186/1752-0509-8-S2-S4 (PMC4101701; doi:10.1186/1752-0509-8-S2-S4)
Supplement: Additional file 8 — GSVD specific GO term associations. PDF file containing the GSVD based specific associations of GO terms to life cycle stages in common space. [file 1752-0509-8-S2-S4-S8.pdf]

PDF file containing the GSVD specific GO term associations in common space.

**Table 1 - GSVD specific GO terms association to sporozoite stage in common space.**

In this table GSVD based GO term association in common space to the cell cycle stage sporozoite are presented.

| GSVD: Sporozoite in common space | |
|---|---|
| GO:0009987 | cellular process |
| GO:0008152 | metabolic process |
| GO:0019538 | protein metabolic process |
| GO:0044237 | cellular metabolic process |
| GO:0009056 | catabolic process |
| GO:0019219 | regulation of nucleobase-containing compound metabolic process |
| GO:0051171 | regulation of nitrogen compound metabolic process |
| GO:0065007 | biological regulation |
| GO:0044238 | primary metabolic process |
| GO:0034645 | cellular macromolecule biosynthetic process |
| GO:0006457 | protein folding |
| GO:0009116 | nucleoside metabolic process |
| GO:0032774 | RNA biosynthetic process |
| GO:0009059 | macromolecule biosynthetic process |
| GO:0006091 | generation of precursor metabolites and energy |

**Table 2 - GSVD specific GO terms association to ring stage in common space.**

In this table GSVD based GO term association in common space to the cell cycle stage ring are presented.

| GSVD: Ring in common space | |
|---|---|
| GO:0008152 | metabolic process |
| GO:0044238 | primary metabolic process |
| GO:0044237 | cellular metabolic process |
| GO:0009987 | cellular process |
| GO:0006091 | generation of precursor metabolites and energy |
| GO:0006412 | translation |
| GO:0006732 | coenzyme metabolic process |
| GO:0009058 | biosynthetic process |
| GO:0055114 | oxidation-reduction process |
| GO:0019538 | protein metabolic process |
| GO:0044249 | cellular biosynthetic process |
| GO:0051186 | cofactor metabolic process |
| GO:0043170 | macromolecule metabolic process |
| GO:0010467 | gene expression |
| GO:0045333 | cellular respiration |

**Table 3 - GSVD specific GO terms association to schizont stage in common space.**

In this table GSVD based GO term association in common space to the cell cycle stage schizont are presented.

| GSVD: Schizont in common space | |
|---|---|
| GO:0009607 | response to biotic stimulus |
| GO:0006952 | defense response |
| GO:0051707 | response to other organism |
| GO:0051805 | evasion or tolerance of immune response of other organism involved in symbiotic interaction |
| GO:0051807 | evasion or tolerance of defense response of other organism involved in symbiotic interaction |
| GO:0051832 | avoidance of defenses of other organism involved in symbiotic interaction |
| GO:0051834 | evasion or tolerance of defenses of other organism involved in symbiotic interaction |
| GO:0052173 | response to defenses of other organism involved in symbiotic interaction |
| GO:0052564 | response to immune response of other organism involved in symbiotic interaction |
| GO:0020033 | antigenic variation |
| GO:0051809 | passive evasion of immune response of other organism involved in symbiotic interaction |
| GO:0051704 | multi-organism process |
| GO:0044403 | symbiosis, encompassing mutualism through parasitism |
| GO:0044419 | interspecies interaction between organisms |
| GO:0055085 | transmembrane transport |

**Table 4 - GSVD specific GO terms association to merozoite stage in common space.**

In this table GSVD based GO term association in common space to the cell cycle stage merozoite are presented.

| GSVD: Merozoite in common space | |
|---|---|
| GO:0044238 | primary metabolic process |
| GO:0009987 | cellular process |
| GO:0008152 | metabolic process |
| GO:0016311 | dephosphorylation |
| GO:0055086 | nucleobase-containing small molecule metabolic process |
| GO:0019637 | organophosphate metabolic process |
| GO:0006753 | nucleoside phosphate metabolic process |
| GO:0044237 | cellular metabolic process |
| GO:0019538 | protein metabolic process |
| GO:0055085 | transmembrane transport |
| GO:0006006 | glucose metabolic process |
| GO:0006470 | protein dephosphorylation |
| GO:0009117 | nucleotide metabolic process |
| GO:0006007 | glucose catabolic process |
| GO:0019320 | hexose catabolic process |
