# Supplementary material for: Integrative omics analysis. A study based on Plasmodium falciparum mRNA and protein data
Source: BMC Syst Biol. 2014 Mar 13;8(Suppl 2):S4. doi: 10.1186/1752-0509-8-S2-S4 (PMC4101701; doi:10.1186/1752-0509-8-S2-S4)
Supplement: Additional file 9 — IBC general GO term associations. PDF file containing the IBC based general associations of GO terms to life cycle stages in common space. [file 1752-0509-8-S2-S4-S9.pdf]

PDF file containing the IBC based general associations of GO terms to cell cycle stages.

Table 1: IBC based general GO terms associations to cell cycle stages.

Stage	GO terms
Ring	GO:0009405, GO:0016337, GO:0020013, GO:0020033, GO:0020035, GO:0042000, GO:0006457, GO:0050776, GO:0030260, GO:0006468, GO:0006412, GO:0006887, GO:0015031, GO:0006508, GO:0006817, GO:0006810, GO:0006511, GO:0019288, GO:0055114, GO:0055085, GO:0007010, GO:0006364, GO:0032259, GO:0006139, GO:0015986, GO:0015991, GO:0006754, GO:0006816, GO:0008152, GO:0006413, GO:0042254, GO:0006886, GO:0060627, GO:0007067, GO:0006414, GO:0006281, GO:0006302, GO:0051603, GO:0006260, GO:0009408, GO:0007021, GO:0006474, GO:0006355, GO:0008283, GO:0006366, GO:0046488, GO:0048015, GO:0006334, GO:0006367, GO:0007018, GO:0007155, GO:0006221, GO:0006417, GO:0006259, GO:0019835, GO:0019836, GO:0006464, GO:0016117, GO:0006289, GO:0007017, GO:0006520, GO:0009058, GO:0006351, GO:0006353, GO:0006397, GO:0008646, GO:0045426, GO:0044237, GO:0042176, GO:0009073, GO:0006383, GO:0006163, GO:0006188, GO:0009152, GO:0016255, GO:0016226, GO:0002377, GO:0009790, GO:0030216, GO:0006633, GO:0006629, GO:0006396, GO:0016114, GO:0006836, GO:0043952, GO:0015718, GO:0016192, GO:0031338, GO:0032889, GO:0008610, GO:0001522, GO:0009451, GO:0006415, GO:0006449, GO:0006855, GO:0030163, GO:0006986, GO:0044267, GO:0006888, GO:0044053, GO:0044409, GO:0006631, GO:0015909, GO:0006267, GO:0006338, GO:0006904, GO:0006869, GO:0006897, GO:0008203, GO:0015992, GO:0046034, GO:0006974, GO:0000027, GO:0000375, GO:0008380, GO:0016042, GO:0000398, GO:0000154, GO:0000059, GO:0018345, GO:0000226, GO:0006467, GO:0045454, GO:0043687, GO:0051246, GO:0017038, GO:0065002, GO:0006979, GO:0006072, GO:0006127, GO:0006950, GO:0016567, GO:0006470, GO:0046823, GO:0060284, GO:0006529, GO:0006486, GO:0006487, GO:0018105, GO:0010564, GO:0030433, GO:0032780, GO:0051131, GO:0002312, GO:0006796, GO:0051301, GO:0006378, GO:0006379, GO:0006096, GO:0015914, GO:0006465, GO:0006333, GO:0006488, GO:0007275, GO:0006370, GO:0019432, GO:0032313, GO:0015908, GO:0016579, GO:0000245, GO:0046907, GO:0016070, GO:0006554, GO:0007035, GO:0009438, GO:0006744, GO:0009234, GO:0006354, GO:0008295, GO:0006261, GO:0051052, GO:0006400, GO:0051276, GO:0015684, GO:0006606, GO:0006811, GO:0006875, GO:0046685, GO:0046939, GO:0006270, GO:0016568, GO:0016480, GO:0017148, GO:0006231, GO:0006545, GO:0006730, GO:0009165, GO:0043248, GO:0006182, GO:0018144, GO:0009059, GO:0051258, GO:0006446, GO:0006626, GO:0045039, GO:0016310, GO:0030036, GO:0060327, GO:0006099, GO:0008643, GO:0006298, GO:0019941, GO:0042787, GO:0000122, GO:0030154, GO:0046323, GO:0006323, GO:0006360, GO:0006310, GO:0046854, GO:0006265, GO:0006021, GO:0008654, GO:0006750, GO:0000917, GO:0009052, GO:0015904, GO:0046677, GO:0009987, GO:0051016, GO:0000079, GO:0045736, GO:0043487, GO:0006818, GO:0048034, GO:0009228, GO:0008104, GO:0042493, GO:0007186, GO:0006607, GO:0006777, GO:0032312, GO:0043087, GO:0009245, GO:0007169, GO:0016043, GO:0018344, GO:0006207, GO:0006222, GO:0051302, GO:0006165, GO:0006183, GO:0006228, GO:0006241, GO:0019368, GO:0006779, GO:0006783, GO:0006743, GO:0006591, GO:0006098, GO:0006357, GO:0034227, GO:0009306, GO:0006284, GO:0006506, GO:0000304, GO:0008614, GO:0042819, GO:0048870, GO:0006090, GO:0006820, GO:0044070, GO:0042128, GO:0009432, GO:0006097, GO:0006352, GO:0008615, GO:0042823, GO:0043412, GO:0007034, GO:0042144, GO:0006122, GO:0045836, GO:0006801, GO:0019430, GO:0006401, GO:0031123, GO:0043631, GO:0051289, GO:0006091, GO:0001682, GO:0006729, GO:0009435, GO:0019357, GO:0019358, GO:0006865, GO:0000082, GO:0000105, GO:0006164, GO:0009086, GO:0009396, GO:0010501, GO:0032508, GO:0006814, GO:0006644, GO:0007154, GO:0015937, GO:0045173, GO:0030261, GO:0006829, GO:0006534, GO:0008616, GO:0030522, GO:0006458, GO:0000280, GO:0042779, GO:0009607, GO:0042113, GO:0007530, GO:0006306, GO:0006614, GO:0045900, GO:0000256, GO:0006144, GO:0000055, GO:0042273, GO:0006536, GO:0009190, GO:0000724, GO:0030259, GO:0006890, GO:0006761, GO:0042558, GO:0006004, GO:0019673, GO:0006086, GO:0019538, GO:0007131, GO:0002720, GO:0006359, GO:0006655, GO:0006103, GO:0009107, GO:0006835, GO:0006839, GO:0015742, GO:0015743, GO:0015858, GO:0007059, GO:0051205, GO:0042127, GO:0051262, GO:0006525, GO:0031365, GO:0043686, GO:0006308, GO:0031167, GO:0007219, GO:0018342, GO:0006269, GO:0046836, GO:0018279, GO:0006556, GO:0006541, GO:0008153, GO:0006542, GO:0006807, GO:0006772, GO:0009229, GO:0016575, GO:0007205, GO:0007020, GO:0015074, GO:0032196, GO:0000184, GO:0007266, GO:0017183, GO:0006493, GO:0006665, GO:0043161, GO:0006788, GO:0006166, GO:0006177, GO:0006914, GO:0009225, GO:0042026, GO:0009186, GO:0000045, GO:0002253, GO:0006094, GO:0007030, GO:0009298, GO:0019307, GO:0006471, GO:0009264, GO:0042773, GO:0006101, GO:0006041, GO:0019353, GO:0009168, GO:0045893, GO:0006596, GO:0006597, GO:0009445, GO:0022900, GO:0006898, GO:0007276, GO:0022904, GO:0016539, GO:0019478, GO:0046777, GO:0006104, GO:0042147, GO:0006271, GO:0015785, GO:0000165, GO:0046168, GO:0042540, GO:0034214, GO:0000338, GO:0007076, GO:0006646, GO:0006656, GO:0030150, GO:0046080, GO:0000070, GO:0008202, GO:0006833, GO:0009247, GO:0015791, GO:0051475, GO:0006546, GO:0006813, GO:0006505, GO:0051604, GO:0030048, GO:0042777, GO:0006891, GO:0010468, GO:0018343, GO:0042776, GO:0001510, GO:0009452, GO:0001819, GO:0006452, GO:0008612, GO:0045901, GO:0045905, GO:0009117, GO:0046069, GO:0033014, GO:0005978, GO:0006108, GO:0006617, GO:0006268, GO:0030071, GO:0015717, GO:0000910, GO:0001932, GO:0015917, GO:0046654, GO:0016458, GO:0018063, GO:0006275, GO:0051259, GO:0018055, GO:0020012, GO:0007015, GO:0006544, GO:0006563, GO:0006481, GO:0006388, GO:0010038, GO:0006928, GO:0006233, GO:0006235, GO:0006032, GO:0000278, GO:0006266, GO:0006273, GO:0006402, GO:0006538, GO:0001906, GO:0031120, GO:0006278, GO:0009249, GO:0006661, GO:0030488, GO:0051298, GO:0045017, GO:0045047, GO:0009056, GO:0006089, GO:0000162, GO:0006342, GO:0006476, GO:0016233, GO:0006196, GO:0006621, GO:0006304, GO:0042256, GO:0007600, GO:0017006, GO:0018106, GO:0018298, GO:0050896, GO:0006447, GO:0000076, GO:0007090, GO:0006047, GO:0016925, GO:0006102, GO:0006863, GO:0032238, GO:0006071, GO:0006167, GO:0006915, GO:0006561, GO:0008154, GO:0006659, GO:0006797, GO:0031119, GO:0006171, GO:0006314, GO:0016574, GO:0006206, GO:0045727, GO:0006479, GO:0050983, GO:0006499, GO:0016049, GO:0035434, GO:0006635, GO:0009062, GO:0016571, GO:0015977, GO:0007050, GO:0030833, GO:0030091, GO:0006537, GO:0006576, GO:0042262, GO:0006944, GO:0032012, GO:0010608, GO:0042594, GO:0045595, GO:0042167, GO:0006384, GO:0048193, GO:0006006, GO:0051156, GO:0006879, GO:0006885, GO:0006662, GO:0002474, GO:0006955, GO:0031952, GO:0045737, GO:0042255, GO:0009187, GO:0008272, GO:0019932, GO:0033205, GO:0006325, GO:0019856, GO:0030497, GO:0009060, GO:0006418, GO:0006422, GO:0007049, GO:0006812, GO:0006184, GO:0006913, GO:0008299, GO:0006461, GO:0006432, GO:0043039, GO:0006428, GO:0006421, GO:0016311, GO:0006438, GO:0009966, GO:0043666, GO:0006429, GO:0006437, GO:0008033, GO:0051726, GO:0030001, GO:0044262, GO:0005975, GO:0006424, GO:0006825, GO:0006878, GO:0008535, GO:0006434, GO:0051028, GO:0006420, GO:0006427, GO:0006431, GO:0006423, GO:0016051, GO:0006405, GO:0006435, GO:0006399, GO:0000902, GO:0008360, GO:0009103, GO:0009252, GO:0006433, GO:0006406, GO:0006611, GO:0006998, GO:0032513, GO:0006436, GO:0006425, GO:0006430, GO:0006419, GO:0006426, GO:0007165, GO:0007264, GO:0035556, GO:0006605, GO:0009116

Continued on next page

Table 1 – continued from previous page

Stage	GO terms
Trophozoite	GO:0009405, GO:0016337, GO:0020013, GO:0020033, GO:0020035, GO:0042000, GO:0006457, GO:0050776, GO:0030260, GO:0006468, GO:0006412, GO:0006887, GO:0015031, GO:0006508, GO:0006817, GO:0006810, GO:0006511, GO:0019288, GO:0055114, GO:0055085, GO:0007010, GO:0006364, GO:0032259, GO:0006139, GO:0015986, GO:0015991, GO:0006754, GO:0006816, GO:0008152, GO:0006413, GO:0042254, GO:0006886, GO:0060627, GO:0007067, GO:0006414, GO:0006281, GO:0006302, GO:0051603, GO:0006260, GO:0009408, GO:0007021, GO:0006474, GO:0006355, GO:0008283, GO:0006366, GO:0046488, GO:0048015, GO:0006334, GO:0006367, GO:0007018, GO:0007155, GO:0006221, GO:0006417, GO:0006259, GO:0019835, GO:0019836, GO:0006464, GO:0016117, GO:0006289, GO:0007017, GO:0006520, GO:0009058, GO:0006351, GO:0006353, GO:0006397, GO:0008646, GO:0045426, GO:0044237, GO:0042176, GO:0009073, GO:0006383, GO:0006163, GO:0006188, GO:0009152, GO:0016255, GO:0016226, GO:0002377, GO:0009790, GO:0030216, GO:0006633, GO:0006629, GO:0006396, GO:0016114, GO:0006836, GO:0043952, GO:0015718, GO:0016192, GO:0031338, GO:0032889, GO:0008610, GO:0001522, GO:0009451, GO:0006415, GO:0006449, GO:0006855, GO:0030163, GO:0006986, GO:0044267, GO:0006888, GO:0044053, GO:0044409, GO:0006631, GO:0015909, GO:0006267, GO:0006338, GO:0006904, GO:0006869, GO:0006897, GO:0008203, GO:0015992, GO:0046034, GO:0006974, GO:0000027, GO:0000375, GO:0008380, GO:0016042, GO:0000398, GO:0000154, GO:0000059, GO:0018345, GO:0000226, GO:0006467, GO:0045454, GO:0043687, GO:0051246, GO:0017038, GO:0065002, GO:0006979, GO:0006072, GO:0006127, GO:0006950, GO:0016567, GO:0006470, GO:0046823, GO:0060284, GO:0006529, GO:0006486, GO:0006487, GO:0018105, GO:0010564, GO:0030433, GO:0032780, GO:0051131, GO:0002312, GO:0006796, GO:0051301, GO:0006378, GO:0006379, GO:0006096, GO:0015914, GO:0006465, GO:0006333, GO:0006488, GO:0007275, GO:0006370, GO:0019432, GO:0032313, GO:0015908, GO:0016579, GO:0000245, GO:0046907, GO:0016070, GO:0006554, GO:0007035, GO:0009438, GO:0006744, GO:0009234, GO:0006354, GO:0008295, GO:0006261, GO:0051052, GO:0006400, GO:0051276, GO:0015684, GO:0006606, GO:0006811, GO:0006875, GO:0046685, GO:0046939, GO:0006270, GO:0016568, GO:0016480, GO:0017148, GO:0006231, GO:0006545, GO:0006730, GO:0009165, GO:0043248, GO:0006182, GO:0018144, GO:0009059, GO:0051258, GO:0006446, GO:0006626, GO:0045039, GO:0016310, GO:0030036, GO:0060327, GO:0006099, GO:0008643, GO:0006298, GO:0019941, GO:0042787, GO:0000122, GO:0030154, GO:0046323, GO:0006323, GO:0006360, GO:0006310, GO:0046854, GO:0006265, GO:0006021, GO:0008654, GO:0006750, GO:0000917, GO:0009052, GO:0015904, GO:0046677, GO:0009987, GO:0051016, GO:0000079, GO:0045736, GO:0043487, GO:0006818, GO:0048034, GO:0009228, GO:0008104, GO:0042493, GO:0007186, GO:0006607, GO:0006777, GO:0032312, GO:0043087, GO:0009245, GO:0007169, GO:0016043, GO:0018344, GO:0006207, GO:0006222, GO:0051302, GO:0006165, GO:0006183, GO:0006228, GO:0006241, GO:0019368, GO:0006779, GO:0006783, GO:0006743, GO:0006591, GO:0006098, GO:0006357, GO:0034227, GO:0009306, GO:0006284, GO:0006506, GO:0000304, GO:0008614, GO:0042819, GO:0048870, GO:0006090, GO:0006820, GO:0044070, GO:0042128, GO:0009432, GO:0006097, GO:0006352, GO:0008615, GO:0042823, GO:0043412, GO:0007034, GO:0042144, GO:0006122, GO:0045836, GO:0006801, GO:0019430, GO:0006401, GO:0031123, GO:0043631, GO:0051289, GO:0006091, GO:0001682, GO:0006729, GO:0009435, GO:0019357, GO:0019358, GO:0006865, GO:0000082, GO:0000105, GO:0006164, GO:0009086, GO:0009396, GO:0010501, GO:0032508, GO:0006814, GO:0006644, GO:0007154, GO:0015937, GO:0045173, GO:0030261, GO:0006829, GO:0006534, GO:0008616, GO:0030522, GO:0006458, GO:0000280, GO:0042779, GO:0009607, GO:0042113, GO:0007530, GO:0006306, GO:0006614, GO:0045900, GO:0000256, GO:0006144, GO:0000055, GO:0042273, GO:0006536, GO:0009190, GO:0000724, GO:0030259, GO:0006890, GO:0006761, GO:0042558, GO:0006004, GO:0019673, GO:0006086, GO:0019538, GO:0007131, GO:0002720, GO:0006359, GO:0006655, GO:0006103, GO:0009107, GO:0006835, GO:0006839, GO:0015742, GO:0015743, GO:0015858, GO:0007059, GO:0051205, GO:0042127, GO:0051262, GO:0006525, GO:0031365, GO:0043686, GO:0006308, GO:0031167, GO:0007219, GO:0018342, GO:0006269, GO:0046836, GO:0018279, GO:0006556, GO:0006541, GO:0008153, GO:0006542, GO:0006807, GO:0006772, GO:0009229, GO:0016575, GO:0007205, GO:0007020, GO:0015074, GO:0032196, GO:0000184, GO:0007266, GO:0017183, GO:0006493, GO:0006665, GO:0043161, GO:0006788, GO:0006166, GO:0006177, GO:0006914, GO:0009225, GO:0042026, GO:0009186, GO:0000045, GO:0002253, GO:0006094, GO:0007030, GO:0009298, GO:0019307, GO:0006471, GO:0009264, GO:0042773, GO:0006101, GO:0006041, GO:0019353, GO:0009168, GO:0045893, GO:0006596, GO:0006597, GO:0009445, GO:0022900, GO:0006898, GO:0007276, GO:0022904, GO:0016539, GO:0019478, GO:0046777, GO:0006104, GO:0042147, GO:0006271, GO:0015785, GO:0000165, GO:0046168, GO:0042540, GO:0034214, GO:0000338, GO:0007076, GO:0006646, GO:0006656, GO:0030150, GO:0046080, GO:0000070, GO:0008202, GO:0006833, GO:0009247, GO:0015791, GO:0051475, GO:0006546, GO:0006813, GO:0006505, GO:0051604, GO:0030048, GO:0042777, GO:0006891, GO:0010468, GO:0018343, GO:0042776, GO:0001510, GO:0009452, GO:0001819, GO:0006452, GO:0008612, GO:0045901, GO:0045905, GO:0009117, GO:0046069, GO:0033014, GO:0005978, GO:0006108, GO:0006617, GO:0006268, GO:0030071, GO:0015717, GO:0000910, GO:0001932, GO:0015917, GO:0046654, GO:0016458, GO:0018063, GO:0006275, GO:0051259, GO:0018055, GO:0020012, GO:0007015, GO:0006544, GO:0006563, GO:0006481, GO:0006388, GO:0010038, GO:0006928, GO:0006233, GO:0006235, GO:0006032, GO:0000278, GO:0006266, GO:0006273, GO:0006402, GO:0006538, GO:0001906, GO:0031120, GO:0006278, GO:0009249, GO:0006661, GO:0030488, GO:0051298, GO:0045017, GO:0045047, GO:0009056, GO:0006089, GO:0000162, GO:0006342, GO:0006476, GO:0016233, GO:0006196, GO:0006621, GO:0006304, GO:0042256, GO:0007600, GO:0017006, GO:0018106, GO:0018298, GO:0050896, GO:0006447, GO:0000076, GO:0007090, GO:0006047, GO:0016925, GO:0006102, GO:0006863, GO:0032238, GO:0006071, GO:0006167, GO:0006915, GO:0006561, GO:0008154, GO:0006659, GO:0006797, GO:0031119, GO:0006171, GO:0006314, GO:0016574, GO:0006206, GO:0045727, GO:0006479, GO:0050983, GO:0006499, GO:0016049, GO:0035434, GO:0006635, GO:0009062, GO:0016571, GO:0015977, GO:0007050, GO:0030833, GO:0030091, GO:0006537, GO:0006576, GO:0042262, GO:0006944, GO:0032012, GO:0010608, GO:0042594, GO:0045595, GO:0042167, GO:0006384, GO:0048193, GO:0006006, GO:0051156, GO:0006879, GO:0006885, GO:0006662, GO:0002474, GO:0006955, GO:0031952, GO:0045737, GO:0042255, GO:0009187, GO:0008272, GO:0019932, GO:0033205, GO:0006325, GO:0019856, GO:0030497, GO:0009060, GO:0006418, GO:0006422, GO:0007049, GO:0006184, GO:0006913, GO:0008299, GO:0006432, GO:0043039, GO:0006428, GO:0006605, GO:0006421, GO:0016311, GO:0006438, GO:0009966, GO:0043666, GO:0006429, GO:0006437, GO:0008033, GO:0051726, GO:0044262, GO:0005975, GO:0006424, GO:0006434, GO:0051028, GO:0006420, GO:0006427, GO:0006431, GO:0006423, GO:0016051, GO:0006405, GO:0006435, GO:0006399, GO:0000902, GO:0008360, GO:0009103, GO:0009252, GO:0006433, GO:0006406, GO:0006611, GO:0006998, GO:0032513, GO:0006436, GO:0006425, GO:0006430, GO:0006419, GO:0006426, GO:0007264, GO:0006461, GO:0009116, GO:0030001, GO:0006825, GO:0006878, GO:0008535, GO:0006812, GO:0007165, GO:0035556

Continued on next page

Table 1 – continued from previous page

Stage	GO terms
Schizont	GO:0009405, GO:0016337, GO:0020013, GO:0020033, GO:0020035, GO:0042000, GO:0006457, GO:0050776, GO:0030260, GO:0006468, GO:0006412, GO:0006887, GO:0015031, GO:0006508, GO:0006817, GO:0006810, GO:0006511, GO:0019288, GO:0055114, GO:0055085, GO:0007010, GO:0006364, GO:0032259, GO:0006139, GO:0015986, GO:0015991, GO:0006754, GO:0006816, GO:0008152, GO:0006413, GO:0042254, GO:0006886, GO:0060627, GO:0007067, GO:0006414, GO:0006281, GO:0006302, GO:0051603, GO:0006260, GO:0009408, GO:0007021, GO:0006474, GO:0006355, GO:0008283, GO:0006366, GO:0046488, GO:0048015, GO:0006334, GO:0006367, GO:0007018, GO:0007155, GO:0006221, GO:0006417, GO:0006259, GO:0019835, GO:0019836, GO:0006464, GO:0016117, GO:0006289, GO:0007017, GO:0006520, GO:0009058, GO:0006351, GO:0006353, GO:0006397, GO:0008646, GO:0045426, GO:0044237, GO:0042176, GO:0009073, GO:0006383, GO:0006163, GO:0006188, GO:0009152, GO:0016255, GO:0016226, GO:0002377, GO:0009790, GO:0030216, GO:0006633, GO:0006629, GO:0006396, GO:0016114, GO:0006836, GO:0043952, GO:0015718, GO:0016192, GO:0031338, GO:0032889, GO:0008610, GO:0001522, GO:0009451, GO:0006415, GO:0006449, GO:0006855, GO:0030163, GO:0006986, GO:0044267, GO:0006888, GO:0044053, GO:0044409, GO:0006631, GO:0015909, GO:0006267, GO:0006338, GO:0006904, GO:0006869, GO:0006897, GO:0008203, GO:0015992, GO:0046034, GO:0006974, GO:0000027, GO:0000375, GO:0008380, GO:0016042, GO:0000398, GO:0000154, GO:0000059, GO:0018345, GO:0000226, GO:0006467, GO:0045454, GO:0043687, GO:0051246, GO:0017038, GO:0065002, GO:0006979, GO:0006072, GO:0006127, GO:0006950, GO:0016567, GO:0006470, GO:0046823, GO:0060284, GO:0006529, GO:0006486, GO:0006487, GO:0018105, GO:0010564, GO:0030433, GO:0032780, GO:0051131, GO:0002312, GO:0006796, GO:0051301, GO:0006378, GO:0006379, GO:0006096, GO:0015914, GO:0006465, GO:0006333, GO:0006488, GO:0007275, GO:0006370, GO:0019432, GO:0032313, GO:0015908, GO:0016579, GO:0000245, GO:0046907, GO:0016070, GO:0006554, GO:0007035, GO:0009438, GO:0006744, GO:0009234, GO:0006354, GO:0008295, GO:0006261, GO:0051052, GO:0006400, GO:0051276, GO:0015684, GO:0006606, GO:0006811, GO:0006875, GO:0046685, GO:0046939, GO:0006270, GO:0016568, GO:0016480, GO:0017148, GO:0006231, GO:0006545, GO:0006730, GO:0009165, GO:0043248, GO:0006182, GO:0018144, GO:0009059, GO:0051258, GO:0006446, GO:0006626, GO:0045039, GO:0016310, GO:0030036, GO:0060327, GO:0006099, GO:0008643, GO:0006298, GO:0019941, GO:0042787, GO:0000122, GO:0030154, GO:0046323, GO:0006323, GO:0006360, GO:0006310, GO:0046854, GO:0006265, GO:0006021, GO:0008654, GO:0006750, GO:0000917, GO:0009052, GO:0015904, GO:0046677, GO:0009987, GO:0051016, GO:0000079, GO:0045736, GO:0043487, GO:0006818, GO:0048034, GO:0009228, GO:0008104, GO:0042493, GO:0007186, GO:0006607, GO:0006777, GO:0032312, GO:0043087, GO:0009245, GO:0007169, GO:0016043, GO:0018344, GO:0006207, GO:0006222, GO:0051302, GO:0006165, GO:0006183, GO:0006228, GO:0006241, GO:0019368, GO:0006779, GO:0006783, GO:0006743, GO:0006591, GO:0006098, GO:0006357, GO:0034227, GO:0009306, GO:0006284, GO:0006506, GO:0000304, GO:0008614, GO:0042819, GO:0048870, GO:0006090, GO:0006820, GO:0044070, GO:0042128, GO:0009432, GO:0006097, GO:0006352, GO:0008615, GO:0042823, GO:0043412, GO:0007034, GO:0042144, GO:0006122, GO:0045836, GO:0006801, GO:0019430, GO:0006401, GO:0031123, GO:0043631, GO:0051289, GO:0006091, GO:0001682, GO:0006729, GO:0009435, GO:0019357, GO:0019358, GO:0006865, GO:0000082, GO:0000105, GO:0006164, GO:0009086, GO:0009396, GO:0010501, GO:0032508, GO:0006814, GO:0006644, GO:0007154, GO:0015937, GO:0045173, GO:0030261, GO:0006829, GO:0006534, GO:0008616, GO:0030522, GO:0006458, GO:0000280, GO:0042779, GO:0009607, GO:0042113, GO:0007530, GO:0006306, GO:0006614, GO:0045900, GO:0000256, GO:0006144, GO:0000055, GO:0042273, GO:0006536, GO:0009190, GO:0000724, GO:0030259, GO:0006890, GO:0006761, GO:0042558, GO:0006004, GO:0019673, GO:0006086, GO:0019538, GO:0007131, GO:0002720, GO:0006359, GO:0006655, GO:0006103, GO:0009107, GO:0006835, GO:0006839, GO:0015742, GO:0015743, GO:0015858, GO:0007059, GO:0051205, GO:0042127, GO:0051262, GO:0006525, GO:0031365, GO:0043686, GO:0006308, GO:0031167, GO:0007219, GO:0018342, GO:0006269, GO:0046836, GO:0018279, GO:0006556, GO:0006541, GO:0008153, GO:0006542, GO:0006807, GO:0006772, GO:0009229, GO:0016575, GO:0007205, GO:0007020, GO:0015074, GO:0032196, GO:0000184, GO:0007266, GO:0017183, GO:0006493, GO:0006665, GO:0043161, GO:0006788, GO:0006166, GO:0006177, GO:0006914, GO:0009225, GO:0042026, GO:0009186, GO:0000045, GO:0002253, GO:0006094, GO:0007030, GO:0009298, GO:0019307, GO:0006471, GO:0009264, GO:0042773, GO:0006101, GO:0006041, GO:0019353, GO:0009168, GO:0045893, GO:0006596, GO:0006597, GO:0009445, GO:0022900, GO:0006898, GO:0007276, GO:0022904, GO:0016539, GO:0019478, GO:0046777, GO:0006104, GO:0042147, GO:0006271, GO:0015785, GO:0000165, GO:0046168, GO:0042540, GO:0034214, GO:0000338, GO:0007076, GO:0006646, GO:0006656, GO:0030150, GO:0046080, GO:0000070, GO:0008202, GO:0006833, GO:0009247, GO:0015791, GO:0051475, GO:0006546, GO:0006813, GO:0006505, GO:0051604, GO:0030048, GO:0042777, GO:0006891, GO:0010468, GO:0018343, GO:0042776, GO:0001510, GO:0009452, GO:0001819, GO:0006452, GO:0008612, GO:0045901, GO:0045905, GO:0009117, GO:0046069, GO:0033014, GO:0005978, GO:0006108, GO:0006617, GO:0006268, GO:0030071, GO:0015717, GO:0000910, GO:0001932, GO:0015917, GO:0046654, GO:0016458, GO:0018063, GO:0006275, GO:0051259, GO:0018055, GO:0020012, GO:0007015, GO:0006544, GO:0006563, GO:0006481, GO:0006388, GO:0010038, GO:0006928, GO:0006233, GO:0006235, GO:0006032, GO:0000278, GO:0006266, GO:0006273, GO:0006402, GO:0006538, GO:0001906, GO:0031120, GO:0006278, GO:0009249, GO:0006661, GO:0030488, GO:0051298, GO:0045017, GO:0045047, GO:0009056, GO:0006089, GO:0000162, GO:0006342, GO:0006476, GO:0016233, GO:0006196, GO:0006621, GO:0006304, GO:0042256, GO:0007600, GO:0017006, GO:0018106, GO:0018298, GO:0050896, GO:0006447, GO:0000076, GO:0007090, GO:0006047, GO:0016925, GO:0006102, GO:0006863, GO:0032238, GO:0006071, GO:0006167, GO:0006915, GO:0006561, GO:0008154, GO:0006659, GO:0006797, GO:0031119, GO:0006171, GO:0006314, GO:0016574, GO:0006206, GO:0045727, GO:0006479, GO:0050983, GO:0006499, GO:0016049, GO:0035434, GO:0006635, GO:0009062, GO:0016571, GO:0015977, GO:0007050, GO:0030833, GO:0030091, GO:0006537, GO:0006576, GO:0042262, GO:0006944, GO:0032012, GO:0010608, GO:0042594, GO:0045595, GO:0042167, GO:0006384, GO:0048193, GO:0006006, GO:0051156, GO:0006879, GO:0006885, GO:0006662, GO:0002474, GO:0006955, GO:0031952, GO:0045737, GO:0042255, GO:0009187, GO:0008272, GO:0019932, GO:0033205, GO:0006325, GO:0019856, GO:0030497, GO:0009060, GO:0006418, GO:0006422, GO:0007049, GO:0006184, GO:0006913, GO:0008299, GO:0006432, GO:0043039, GO:0006428, GO:0006605, GO:0006421, GO:0016311, GO:0006438, GO:0009966, GO:0043666, GO:0006429, GO:0006437, GO:0008033, GO:0051726, GO:0044262, GO:0005975, GO:0006424, GO:0006434, GO:0051028, GO:0006420, GO:0006427, GO:0006431, GO:0006423, GO:0016051, GO:0006405, GO:0006435, GO:0006399, GO:0000902, GO:0008360, GO:0009103, GO:0009252, GO:0006433, GO:0006406, GO:0006611, GO:0006998, GO:0032513, GO:0006436, GO:0006425, GO:0006430, GO:0006419, GO:0006426, GO:0007264, GO:0006461, GO:0009116, GO:0030001, GO:0006825, GO:0006878, GO:0008535, GO:0006812, GO:0007165, GO:0035556

Continued on next page

Table 1 – continued from previous page

Stage	GO terms
Merozoite	GO:0009405, GO:0016337, GO:0020013, GO:0020033, GO:0020035, GO:0042000, GO:0006457, GO:0050776, GO:0030260, GO:0006468, GO:0006412, GO:0006887, GO:0015031, GO:0006508, GO:0006817, GO:0006810, GO:0006511, GO:0019288, GO:0055114, GO:0055085, GO:0007010, GO:0006364, GO:0032259, GO:0006139, GO:0015986, GO:0015991, GO:0006754, GO:0006816, GO:0008152, GO:0006413, GO:0042254, GO:0006886, GO:0060627, GO:0007067, GO:0006414, GO:0006281, GO:0006302, GO:0051603, GO:0006260, GO:0009408, GO:0007021, GO:0006474, GO:0006355, GO:0008283, GO:0006366, GO:0046488, GO:0048015, GO:0006334, GO:0006367, GO:0007018, GO:0007155, GO:0006221, GO:0006417, GO:0006259, GO:0019835, GO:0019836, GO:0006464, GO:0016117, GO:0006289, GO:0007017, GO:0006520, GO:0009058, GO:0006351, GO:0006353, GO:0006397, GO:0008646, GO:0045426, GO:0044237, GO:0042176, GO:0009073, GO:0006383, GO:0006163, GO:0006188, GO:0009152, GO:0016255, GO:0016226, GO:0002377, GO:0009790, GO:0030216, GO:0006633, GO:0006629, GO:0006396, GO:0016114, GO:0006836, GO:0043952, GO:0015718, GO:0016192, GO:0031338, GO:0032889, GO:0008610, GO:0001522, GO:0009451, GO:0006415, GO:0006449, GO:0006855, GO:0030163, GO:0006986, GO:0044267, GO:0006888, GO:0044053, GO:0044409, GO:0006631, GO:0015909, GO:0006267, GO:0006338, GO:0006904, GO:0006869, GO:0006897, GO:0008203, GO:0015992, GO:0046034, GO:0006974, GO:0000027, GO:0000375, GO:0008380, GO:0016042, GO:0000398, GO:0000154, GO:0000059, GO:0018345, GO:0000226, GO:0006467, GO:0045454, GO:0043687, GO:0051246, GO:0017038, GO:0065002, GO:0006979, GO:0006072, GO:0006127, GO:0006950, GO:0016567, GO:0006470, GO:0046823, GO:0060284, GO:0006529, GO:0006486, GO:0006487, GO:0018105, GO:0010564, GO:0030433, GO:0032780, GO:0051131, GO:0002312, GO:0006796, GO:0051301, GO:0006378, GO:0006379, GO:0006096, GO:0015914, GO:0006465, GO:0006333, GO:0006488, GO:0007275, GO:0006370, GO:0019432, GO:0032313, GO:0015908, GO:0016579, GO:0000245, GO:0046907, GO:0016070, GO:0006554, GO:0007035, GO:0009438, GO:0006744, GO:0009234, GO:0006354, GO:0008295, GO:0006261, GO:0051052, GO:0006400, GO:0051276, GO:0015684, GO:0006606, GO:0006811, GO:0006875, GO:0046685, GO:0046939, GO:0006270, GO:0016568, GO:0016480, GO:0017148, GO:0006231, GO:0006545, GO:0006730, GO:0009165, GO:0043248, GO:0006182, GO:0018144, GO:0009059, GO:0051258, GO:0006446, GO:0006626, GO:0045039, GO:0016310, GO:0030036, GO:0060327, GO:0006099, GO:0008643, GO:0006298, GO:0019941, GO:0042787, GO:0000122, GO:0030154, GO:0046323, GO:0006323, GO:0006360, GO:0006310, GO:0046854, GO:0006265, GO:0006021, GO:0008654, GO:0006750, GO:0000917, GO:0009052, GO:0015904, GO:0046677, GO:0009987, GO:0051016, GO:0000079, GO:0045736, GO:0043487, GO:0006818, GO:0048034, GO:0009228, GO:0008104, GO:0042493, GO:0007186, GO:0006607, GO:0006777, GO:0032312, GO:0043087, GO:0009245, GO:0007169, GO:0016043, GO:0018344, GO:0006207, GO:0006222, GO:0051302, GO:0006165, GO:0006183, GO:0006228, GO:0006241, GO:0019368, GO:0006779, GO:0006783, GO:0006743, GO:0006591, GO:0006098, GO:0006357, GO:0034227, GO:0009306, GO:0006284, GO:0006506, GO:0000304, GO:0008614, GO:0042819, GO:0048870, GO:0006090, GO:0006820, GO:0044070, GO:0042128, GO:0009432, GO:0006097, GO:0006352, GO:0008615, GO:0042823, GO:0043412, GO:0007034, GO:0042144, GO:0006122, GO:0045836, GO:0006801, GO:0019430, GO:0006401, GO:0031123, GO:0043631, GO:0051289, GO:0006091, GO:0001682, GO:0006729, GO:0009435, GO:0019357, GO:0019358, GO:0006865, GO:0000082, GO:0000105, GO:0006164, GO:0009086, GO:0009396, GO:0010501, GO:0032508, GO:0006814, GO:0006644, GO:0007154, GO:0015937, GO:0045173, GO:0030261, GO:0006829, GO:0006534, GO:0008616, GO:0030522, GO:0006458, GO:0000280, GO:0042779, GO:0009607, GO:0042113, GO:0007530, GO:0006306, GO:0006614, GO:0045900, GO:0000256, GO:0006144, GO:0000055, GO:0042273, GO:0006536, GO:0009190, GO:0000724, GO:0030259, GO:0006890, GO:0006761, GO:0042558, GO:0006004, GO:0019673, GO:0006086, GO:0019538, GO:0007131, GO:0002720, GO:0006359, GO:0006655, GO:0006103, GO:0009107, GO:0006835, GO:0006839, GO:0015742, GO:0015743, GO:0015858, GO:0007059, GO:0051205, GO:0042127, GO:0051262, GO:0006525, GO:0031365, GO:0043686, GO:0006308, GO:0031167, GO:0007219, GO:0018342, GO:0006269, GO:0046836, GO:0018279, GO:0006556, GO:0006541, GO:0008153, GO:0006542, GO:0006807, GO:0006772, GO:0009229, GO:0016575, GO:0007205, GO:0007020, GO:0015074, GO:0032196, GO:0000184, GO:0007266, GO:0017183, GO:0006493, GO:0006665, GO:0043161, GO:0006788, GO:0006166, GO:0006177, GO:0006914, GO:0009225, GO:0042026, GO:0009186, GO:0000045, GO:0002253, GO:0006094, GO:0007030, GO:0009298, GO:0019307, GO:0006471, GO:0009264, GO:0042773, GO:0006101, GO:0006041, GO:0019353, GO:0009168, GO:0045893, GO:0006596, GO:0006597, GO:0009445, GO:0022900, GO:0006898, GO:0007276, GO:0022904, GO:0016539, GO:0019478, GO:0046777, GO:0006104, GO:0042147, GO:0006271, GO:0015785, GO:0000165, GO:0046168, GO:0042540, GO:0034214, GO:0000338, GO:0007076, GO:0006646, GO:0006656, GO:0030150, GO:0046080, GO:0000070, GO:0008202, GO:0006833, GO:0009247, GO:0015791, GO:0051475, GO:0006546, GO:0006813, GO:0006505, GO:0051604, GO:0030048, GO:0042777, GO:0006891, GO:0010468, GO:0018343, GO:0042776, GO:0001510, GO:0009452, GO:0001819, GO:0006452, GO:0008612, GO:0045901, GO:0045905, GO:0009117, GO:0046069, GO:0033014, GO:0005978, GO:0006108, GO:0006617, GO:0006268, GO:0030071, GO:0015717, GO:0000910, GO:0001932, GO:0015917, GO:0046654, GO:0016458, GO:0018063, GO:0006275, GO:0051259, GO:0018055, GO:0020012, GO:0007015, GO:0006544, GO:0006563, GO:0006481, GO:0006388, GO:0010038, GO:0006928, GO:0006233, GO:0006235, GO:0006032, GO:0000278, GO:0006266, GO:0006273, GO:0006402, GO:0006538, GO:0001906, GO:0031120, GO:0006278, GO:0009249, GO:0006661, GO:0030488, GO:0051298, GO:0045017, GO:0045047, GO:0009056, GO:0006089, GO:0000162, GO:0006342, GO:0006476, GO:0016233, GO:0006196, GO:0006621, GO:0006304, GO:0042256, GO:0007600, GO:0017006, GO:0018106, GO:0018298, GO:0050896, GO:0006447, GO:0000076, GO:0007090, GO:0006047, GO:0016925, GO:0006102, GO:0006863, GO:0032238, GO:0006071, GO:0006167, GO:0006915, GO:0006561, GO:0008154, GO:0006659, GO:0006797, GO:0031119, GO:0006171, GO:0006314, GO:0016574, GO:0006206, GO:0045727, GO:0006479, GO:0050983, GO:0006499, GO:0016049, GO:0035434, GO:0006635, GO:0009062, GO:0016571, GO:0015977, GO:0007050, GO:0030833, GO:0030091, GO:0006537, GO:0006576, GO:0042262, GO:0006944, GO:0032012, GO:0010608, GO:0042594, GO:0045595, GO:0042167, GO:0006384, GO:0048193, GO:0006006, GO:0051156, GO:0006879, GO:0006885, GO:0006662, GO:0002474, GO:0006955, GO:0031952, GO:0045737, GO:0042255, GO:0009187, GO:0008272, GO:0019932, GO:0033205, GO:0006325, GO:0019856, GO:0030497, GO:0009060, GO:0006418, GO:0006422, GO:0007049, GO:0006812, GO:0006184, GO:0006913, GO:0008299, GO:0006461, GO:0006432, GO:0043039, GO:0006428, GO:0006421, GO:0016311, GO:0006438, GO:0009966, GO:0043666, GO:0006429, GO:0006437, GO:0008033, GO:0051726, GO:0030001, GO:0044262, GO:0005975, GO:0006424, GO:0006825, GO:0006878, GO:0008535, GO:0006434, GO:0051028, GO:0006420, GO:0006427, GO:0006431, GO:0006423, GO:0016051, GO:0006405, GO:0006435, GO:0006399, GO:0000902, GO:0008360, GO:0009103, GO:0009252, GO:0006433, GO:0006406, GO:0006611, GO:0006998, GO:0032513, GO:0006436, GO:0006425, GO:0006430, GO:0006419, GO:0006426, GO:0007165, GO:0007264, GO:0035556, GO:0006605, GO:0009116

Continued on next page

Table 1 – continued from previous page

Stage	GO terms
Gametocyte	GO:0009405, GO:0016337, GO:0020013, GO:0020033, GO:0020035, GO:0042000, GO:0006457, GO:0050776, GO:0030260, GO:0006468, GO:0006412, GO:0006887, GO:0015031, GO:0006508, GO:0006817, GO:0006810, GO:0006511, GO:0019288, GO:0055114, GO:0055085, GO:0007010, GO:0006364, GO:0032259, GO:0006139, GO:0015986, GO:0015991, GO:0006754, GO:0006816, GO:0008152, GO:0006413, GO:0042254, GO:0006886, GO:0060627, GO:0007067, GO:0006414, GO:0006281, GO:0006302, GO:0051603, GO:0006260, GO:0009408, GO:0007021, GO:0006474, GO:0006355, GO:0008283, GO:0006366, GO:0046488, GO:0048015, GO:0006334, GO:0006367, GO:0007018, GO:0007155, GO:0006221, GO:0006417, GO:0006259, GO:0019835, GO:0019836, GO:0006464, GO:0016117, GO:0006289, GO:0007017, GO:0006520, GO:0009058, GO:0006351, GO:0006353, GO:0006397, GO:0008646, GO:0045426, GO:0044237, GO:0042176, GO:0009073, GO:0006383, GO:0006163, GO:0006188, GO:0009152, GO:0016255, GO:0016226, GO:0002377, GO:0009790, GO:0030216, GO:0006633, GO:0006629, GO:0006396, GO:0016114, GO:0006836, GO:0043952, GO:0015718, GO:0016192, GO:0031338, GO:0032889, GO:0008610, GO:0001522, GO:0009451, GO:0006415, GO:0006449, GO:0006855, GO:0030163, GO:0006986, GO:0044267, GO:0006888, GO:0044053, GO:0044409, GO:0006631, GO:0015909, GO:0006267, GO:0006338, GO:0006904, GO:0006869, GO:0006897, GO:0008203, GO:0015992, GO:0046034, GO:0006974, GO:0000027, GO:0000375, GO:0008380, GO:0016042, GO:0000398, GO:0000154, GO:0000059, GO:0018345, GO:0000226, GO:0006467, GO:0045454, GO:0043687, GO:0051246, GO:0017038, GO:0065002, GO:0006979, GO:0006072, GO:0006127, GO:0006950, GO:0016567, GO:0006470, GO:0046823, GO:0060284, GO:0006529, GO:0006486, GO:0006487, GO:0018105, GO:0010564, GO:0030433, GO:0032780, GO:0051131, GO:0002312, GO:0006796, GO:0051301, GO:0006378, GO:0006379, GO:0006096, GO:0015914, GO:0006465, GO:0006333, GO:0006488, GO:0007275, GO:0006370, GO:0019432, GO:0032313, GO:0015908, GO:0016579, GO:0000245, GO:0046907, GO:0016070, GO:0006554, GO:0007035, GO:0009438, GO:0006744, GO:0009234, GO:0006354, GO:0008295, GO:0006261, GO:0051052, GO:0006400, GO:0051276, GO:0015684, GO:0006606, GO:0006811, GO:0006875, GO:0046685, GO:0046939, GO:0006270, GO:0016568, GO:0016480, GO:0017148, GO:0006231, GO:0006545, GO:0006730, GO:0009165, GO:0043248, GO:0006182, GO:0018144, GO:0009059, GO:0051258, GO:0006446, GO:0006626, GO:0045039, GO:0016310, GO:0030036, GO:0060327, GO:0006099, GO:0008643, GO:0006298, GO:0019941, GO:0042787, GO:0000122, GO:0030154, GO:0046323, GO:0006323, GO:0006360, GO:0006310, GO:0046854, GO:0006265, GO:0006021, GO:0008654, GO:0006750, GO:0000917, GO:0009052, GO:0015904, GO:0046677, GO:0009987, GO:0051016, GO:0000079, GO:0045736, GO:0043487, GO:0006818, GO:0048034, GO:0009228, GO:0008104, GO:0042493, GO:0007186, GO:0006607, GO:0006777, GO:0032312, GO:0043087, GO:0009245, GO:0007169, GO:0016043, GO:0018344, GO:0006207, GO:0006222, GO:0051302, GO:0006165, GO:0006183, GO:0006228, GO:0006241, GO:0019368, GO:0006779, GO:0006783, GO:0006743, GO:0006591, GO:0006098, GO:0006357, GO:0034227, GO:0009306, GO:0006284, GO:0006506, GO:0000304, GO:0008614, GO:0042819, GO:0048870, GO:0006090, GO:0006820, GO:0044070, GO:0042128, GO:0009432, GO:0006097, GO:0006352, GO:0008615, GO:0042823, GO:0043412, GO:0007034, GO:0042144, GO:0006122, GO:0045836, GO:0006801, GO:0019430, GO:0006401, GO:0031123, GO:0043631, GO:0051289, GO:0006091, GO:0001682, GO:0006729, GO:0009435, GO:0019357, GO:0019358, GO:0006865, GO:0000082, GO:0000105, GO:0006164, GO:0009086, GO:0009396, GO:0010501, GO:0032508, GO:0006814, GO:0006644, GO:0007154, GO:0015937, GO:0045173, GO:0030261, GO:0006829, GO:0006534, GO:0008616, GO:0030522, GO:0006458, GO:0000280, GO:0042779, GO:0009607, GO:0042113, GO:0007530, GO:0006306, GO:0006614, GO:0045900, GO:0000256, GO:0006144, GO:0000055, GO:0042273, GO:0006536, GO:0009190, GO:0000724, GO:0030259, GO:0006890, GO:0006761, GO:0042558, GO:0006004, GO:0019673, GO:0006086, GO:0019538, GO:0007131, GO:0002720, GO:0006359, GO:0006655, GO:0006103, GO:0009107, GO:0006835, GO:0006839, GO:0015742, GO:0015743, GO:0015858, GO:0007059, GO:0051205, GO:0042127, GO:0051262, GO:0006525, GO:0031365, GO:0043686, GO:0006308, GO:0031167, GO:0007219, GO:0018342, GO:0006269, GO:0046836, GO:0018279, GO:0006556, GO:0006541, GO:0008153, GO:0006542, GO:0006807, GO:0006772, GO:0009229, GO:0016575, GO:0007205, GO:0007020, GO:0015074, GO:0032196, GO:0000184, GO:0007266, GO:0017183, GO:0006493, GO:0006665, GO:0043161, GO:0006788, GO:0006166, GO:0006177, GO:0006914, GO:0009225, GO:0042026, GO:0009186, GO:0000045, GO:0002253, GO:0006094, GO:0007030, GO:0009298, GO:0019307, GO:0006471, GO:0009264, GO:0042773, GO:0006101, GO:0006041, GO:0019353, GO:0009168, GO:0045893, GO:0006596, GO:0006597, GO:0009445, GO:0022900, GO:0006898, GO:0007276, GO:0022904, GO:0016539, GO:0019478, GO:0046777, GO:0006104, GO:0042147, GO:0006271, GO:0015785, GO:0000165, GO:0046168, GO:0042540, GO:0034214, GO:0000338, GO:0007076, GO:0006646, GO:0006656, GO:0030150, GO:0046080, GO:0000070, GO:0008202, GO:0006833, GO:0009247, GO:0015791, GO:0051475, GO:0006546, GO:0006813, GO:0006505, GO:0051604, GO:0030048, GO:0042777, GO:0006891, GO:0010468, GO:0018343, GO:0042776, GO:0001510, GO:0009452, GO:0001819, GO:0006452, GO:0008612, GO:0045901, GO:0045905, GO:0009117, GO:0046069, GO:0033014, GO:0005978, GO:0006108, GO:0006617, GO:0006268, GO:0030071, GO:0015717, GO:0000910, GO:0001932, GO:0015917, GO:0046654, GO:0016458, GO:0018063, GO:0006275, GO:0051259, GO:0018055, GO:0020012, GO:0007015, GO:0006544, GO:0006563, GO:0006481, GO:0006388, GO:0010038, GO:0006928, GO:0006233, GO:0006235, GO:0006032, GO:0000278, GO:0006266, GO:0006273, GO:0006402, GO:0006538, GO:0001906, GO:0031120, GO:0006278, GO:0009249, GO:0006661, GO:0030488, GO:0051298, GO:0045017, GO:0045047, GO:0009056, GO:0006089, GO:0000162, GO:0006342, GO:0006476, GO:0016233, GO:0006196, GO:0006621, GO:0006304, GO:0042256, GO:0007600, GO:0017006, GO:0018106, GO:0018298, GO:0050896, GO:0006447, GO:0000076, GO:0007090, GO:0006047, GO:0016925, GO:0006102, GO:0006863, GO:0032238, GO:0006071, GO:0006167, GO:0006915, GO:0006561, GO:0008154, GO:0006659, GO:0006797, GO:0031119, GO:0006171, GO:0006314, GO:0016574, GO:0006206, GO:0045727, GO:0006479, GO:0050983, GO:0006499, GO:0016049, GO:0035434, GO:0006635, GO:0009062, GO:0016571, GO:0015977, GO:0007050, GO:0030833, GO:0030091, GO:0006537, GO:0006576, GO:0042262, GO:0006944, GO:0032012, GO:0010608, GO:0042594, GO:0045595, GO:0042167, GO:0006384, GO:0048193, GO:0006006, GO:0051156, GO:0006879, GO:0006885, GO:0006662, GO:0002474, GO:0006955, GO:0031952, GO:0045737, GO:0042255, GO:0009187, GO:0008272, GO:0019932, GO:0033205, GO:0006325, GO:0019856, GO:0030497, GO:0009060, GO:0006418, GO:0006422, GO:0007049, GO:0006812, GO:0006184, GO:0006913, GO:0008299, GO:0006461, GO:0006432, GO:0043039, GO:0006428, GO:0006421, GO:0016311, GO:0006438, GO:0009966, GO:0043666, GO:0006429, GO:0006437, GO:0008033, GO:0051726, GO:0030001, GO:0044262, GO:0005975, GO:0006424, GO:0006825, GO:0006878, GO:0008535, GO:0006434, GO:0051028, GO:0006420, GO:0006427, GO:0006431, GO:0006423, GO:0016051, GO:0006405, GO:0006435, GO:0006399, GO:0000902, GO:0008360, GO:0009103, GO:0009252, GO:0006433, GO:0006406, GO:0006611, GO:0006998, GO:0032513, GO:0006436, GO:0006425, GO:0006430, GO:0006419, GO:0006426, GO:0007165, GO:0007264, GO:0035556, GO:0006605, GO:0009116

Continued on next page

Table 1 – continued from previous page

Stage	GO terms
Sporozoite	GO:0009405, GO:0016337, GO:0020013, GO:0020033, GO:0020035, GO:0042000, GO:0006457, GO:0050776, GO:0030260, GO:0006468, GO:0006412, GO:0006887, GO:0015031, GO:0006508, GO:0006817, GO:0006810, GO:0006511, GO:0019288, GO:0055114, GO:0055085, GO:0007010, GO:0006364, GO:0032259, GO:0006139, GO:0015986, GO:0015991, GO:0006754, GO:0006816, GO:0008152, GO:0006413, GO:0042254, GO:0006886, GO:0060627, GO:0007067, GO:0006414, GO:0006281, GO:0006302, GO:0051603, GO:0006260, GO:0009408, GO:0007021, GO:0006474, GO:0006355, GO:0008283, GO:0006366, GO:0046488, GO:0048015, GO:0006334, GO:0006367, GO:0007018, GO:0007155, GO:0006221, GO:0006417, GO:0006259, GO:0019835, GO:0019836, GO:0006464, GO:0016117, GO:0006289, GO:0007017, GO:0006520, GO:0009058, GO:0006351, GO:0006353, GO:0006397, GO:0008646, GO:0045426, GO:0044237, GO:0042176, GO:0009073, GO:0006383, GO:0006163, GO:0006188, GO:0009152, GO:0016255, GO:0016226, GO:0002377, GO:0009790, GO:0030216, GO:0006633, GO:0006629, GO:0006396, GO:0016114, GO:0006836, GO:0043952, GO:0015718, GO:0016192, GO:0031338, GO:0032889, GO:0008610, GO:0001522, GO:0009451, GO:0006415, GO:0006449, GO:0006855, GO:0030163, GO:0006986, GO:0044267, GO:0006888, GO:0044053, GO:0044409, GO:0006631, GO:0015909, GO:0006267, GO:0006338, GO:0006904, GO:0006869, GO:0006897, GO:0008203, GO:0015992, GO:0046034, GO:0006974, GO:0000027, GO:0000375, GO:0008380, GO:0016042, GO:0000398, GO:0000154, GO:0000059, GO:0018345, GO:0000226, GO:0006467, GO:0045454, GO:0043687, GO:0051246, GO:0017038, GO:0065002, GO:0006979, GO:0006072, GO:0006127, GO:0006950, GO:0016567, GO:0006470, GO:0046823, GO:0060284, GO:0006529, GO:0006486, GO:0006487, GO:0018105, GO:0010564, GO:0030433, GO:0032780, GO:0051131, GO:0002312, GO:0006796, GO:0051301, GO:0006378, GO:0006379, GO:0006096, GO:0015914, GO:0006465, GO:0006333, GO:0006488, GO:0007275, GO:0006370, GO:0019432, GO:0032313, GO:0015908, GO:0016579, GO:0000245, GO:0046907, GO:0016070, GO:0006554, GO:0007035, GO:0009438, GO:0006744, GO:0009234, GO:0006354, GO:0008295, GO:0006261, GO:0051052, GO:0006400, GO:0051276, GO:0015684, GO:0006606, GO:0006811, GO:0006875, GO:0046685, GO:0046939, GO:0006270, GO:0016568, GO:0016480, GO:0017148, GO:0006231, GO:0006545, GO:0006730, GO:0009165, GO:0043248, GO:0006182, GO:0018144, GO:0009059, GO:0051258, GO:0006446, GO:0006626, GO:0045039, GO:0016310, GO:0030036, GO:0060327, GO:0006099, GO:0008643, GO:0006298, GO:0019941, GO:0042787, GO:0000122, GO:0030154, GO:0046323, GO:0006323, GO:0006360, GO:0006310, GO:0046854, GO:0006265, GO:0006021, GO:0008654, GO:0006750, GO:0000917, GO:0009052, GO:0015904, GO:0046677, GO:0009987, GO:0051016, GO:0000079, GO:0045736, GO:0043487, GO:0006818, GO:0048034, GO:0009228, GO:0008104, GO:0042493, GO:0007186, GO:0006607, GO:0006777, GO:0032312, GO:0043087, GO:0009245, GO:0007169, GO:0016043, GO:0018344, GO:0006207, GO:0006222, GO:0051302, GO:0006165, GO:0006183, GO:0006228, GO:0006241, GO:0019368, GO:0006779, GO:0006783, GO:0006743, GO:0006591, GO:0006098, GO:0006357, GO:0034227, GO:0009306, GO:0006284, GO:0006506, GO:0000304, GO:0008614, GO:0042819, GO:0048870, GO:0006090, GO:0006820, GO:0044070, GO:0042128, GO:0009432, GO:0006097, GO:0006352, GO:0008615, GO:0042823, GO:0043412, GO:0007034, GO:0042144, GO:0006122, GO:0045836, GO:0006801, GO:0019430, GO:0006401, GO:0031123, GO:0043631, GO:0051289, GO:0006091, GO:0001682, GO:0006729, GO:0009435, GO:0019357, GO:0019358, GO:0006865, GO:0000082, GO:0000105, GO:0006164, GO:0009086, GO:0009396, GO:0010501, GO:0032508, GO:0006814, GO:0006644, GO:0007154, GO:0015937, GO:0045173, GO:0030261, GO:0006829, GO:0006534, GO:0008616, GO:0030522, GO:0006458, GO:0000280, GO:0042779, GO:0009607, GO:0042113, GO:0007530, GO:0006306, GO:0006614, GO:0045900, GO:0000256, GO:0006144, GO:0000055, GO:0042273, GO:0006536, GO:0009190, GO:0000724, GO:0030259, GO:0006890, GO:0006761, GO:0042558, GO:0006004, GO:0019673, GO:0006086, GO:0019538, GO:0007131, GO:0002720, GO:0006359, GO:0006655, GO:0006103, GO:0009107, GO:0006835, GO:0006839, GO:0015742, GO:0015743, GO:0015858, GO:0007059, GO:0051205, GO:0042127, GO:0051262, GO:0006525, GO:0031365, GO:0043686, GO:0006308, GO:0031167, GO:0007219, GO:0018342, GO:0006269, GO:0046836, GO:0018279, GO:0006556, GO:0006541, GO:0008153, GO:0006542, GO:0006807, GO:0006772, GO:0009229, GO:0016575, GO:0007205, GO:0007020, GO:0015074, GO:0032196, GO:0000184, GO:0007266, GO:0017183, GO:0006493, GO:0006665, GO:0043161, GO:0006788, GO:0006166, GO:0006177, GO:0006914, GO:0009225, GO:0042026, GO:0009186, GO:0000045, GO:0002253, GO:0006094, GO:0007030, GO:0009298, GO:0019307, GO:0006471, GO:0009264, GO:0042773, GO:0006101, GO:0006041, GO:0019353, GO:0009168, GO:0045893, GO:0006596, GO:0006597, GO:0009445, GO:0022900, GO:0006898, GO:0007276, GO:0022904, GO:0016539, GO:0019478, GO:0046777, GO:0006104, GO:0042147, GO:0006271, GO:0015785, GO:0000165, GO:0046168, GO:0042540, GO:0034214, GO:0000338, GO:0007076, GO:0006646, GO:0006656, GO:0030150, GO:0046080, GO:0000070, GO:0008202, GO:0006833, GO:0009247, GO:0015791, GO:0051475, GO:0006546, GO:0006813, GO:0006505, GO:0051604, GO:0030048, GO:0042777, GO:0006891, GO:0010468, GO:0018343, GO:0042776, GO:0001510, GO:0009452, GO:0001819, GO:0006452, GO:0008612, GO:0045901, GO:0045905, GO:0009117, GO:0046069, GO:0033014, GO:0005978, GO:0006108, GO:0006617, GO:0006268, GO:0030071, GO:0015717, GO:0000910, GO:0001932, GO:0015917, GO:0046654, GO:0016458, GO:0018063, GO:0006275, GO:0051259, GO:0018055, GO:0020012, GO:0007015, GO:0006544, GO:0006563, GO:0006481, GO:0006388, GO:0010038, GO:0006928, GO:0006233, GO:0006235, GO:0006032, GO:0000278, GO:0006266, GO:0006273, GO:0006402, GO:0006538, GO:0001906, GO:0031120, GO:0006278, GO:0009249, GO:0006661, GO:0030488, GO:0051298, GO:0045017, GO:0045047, GO:0009056, GO:0006089, GO:0000162, GO:0006342, GO:0006476, GO:0016233, GO:0006196, GO:0006621, GO:0006304, GO:0042256, GO:0007600, GO:0017006, GO:0018106, GO:0018298, GO:0050896, GO:0006447, GO:0000076, GO:0007090, GO:0006047, GO:0016925, GO:0006102, GO:0006863, GO:0032238, GO:0006071, GO:0006167, GO:0006915, GO:0006561, GO:0008154, GO:0006659, GO:0006797, GO:0031119, GO:0006171, GO:0006314, GO:0016574, GO:0006206, GO:0045727, GO:0006479, GO:0050983, GO:0006499, GO:0016049, GO:0035434, GO:0006635, GO:0009062, GO:0016571, GO:0015977, GO:0007050, GO:0030833, GO:0030091, GO:0006537, GO:0006576, GO:0042262, GO:0006944, GO:0032012, GO:0010608, GO:0042594, GO:0045595, GO:0042167, GO:0006384, GO:0048193, GO:0006006, GO:0051156, GO:0006879, GO:0006885, GO:0006662, GO:0002474, GO:0006955, GO:0031952, GO:0045737, GO:0042255, GO:0009187, GO:0008272, GO:0019932, GO:0033205, GO:0006325, GO:0019856, GO:0030497, GO:0009060, GO:0006418, GO:0006422, GO:0007049, GO:0006184, GO:0006913, GO:0008299, GO:0006432, GO:0043039, GO:0006428, GO:0006605, GO:0006421, GO:0016311, GO:0006438, GO:0009966, GO:0043666, GO:0006429, GO:0006437, GO:0008033, GO:0051726, GO:0044262, GO:0005975, GO:0006424, GO:0006434, GO:0051028, GO:0006420, GO:0006427, GO:0006431, GO:0006423, GO:0016051, GO:0006405, GO:0006435, GO:0006399, GO:0000902, GO:0008360, GO:0009103, GO:0009252, GO:0006433, GO:0006406, GO:0006611, GO:0006998, GO:0032513, GO:0006436, GO:0006425, GO:0006430, GO:0006419, GO:0006426, GO:0007264, GO:0006461, GO:0009116, GO:0030001, GO:0006825, GO:0006878, GO:0008535, GO:0006812, GO:0007165, GO:0035556

—l—l—
